# MF-PCBA: Multifidelity High-Throughput Screening Benchmarks for Drug Discovery and Machine Learning

**DOI:** 10.1021/acs.jcim.2c01569

**Published:** 2023-04-14

**Authors:** David Buterez, Jon Paul Janet, Steven J. Kiddle, Pietro Liò

**Affiliations:** † Department of Computer Science and Technology, University of Cambridge, Cambridge CB3 0FD, U.K.; ‡ Molecular AI, Discovery Sciences, R&D, AstraZeneca, 431 50 Gothenburg, Sweden; § Data Science & Advanced Analytics, Data Science & Artificial Intelligence, R&D, AstraZeneca, Cambridge CB2 8PA, U.K.

## Abstract

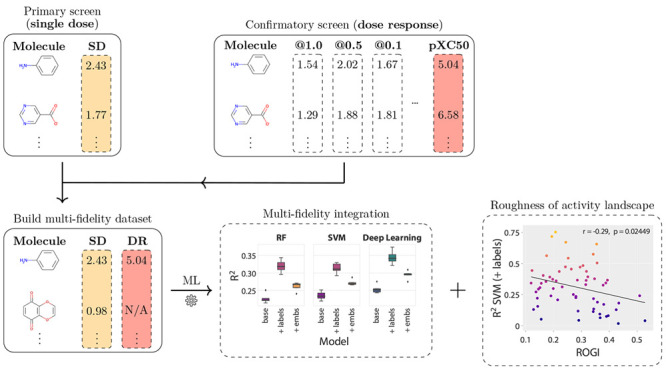

High-throughput screening (HTS), as one of the key techniques in drug discovery, is frequently used to identify promising drug candidates in a largely automated and cost-effective way. One of the necessary conditions for successful HTS campaigns is a large and diverse compound library, enabling hundreds of thousands of activity measurements per project. Such collections of data hold great promise for computational and experimental drug discovery efforts, especially when leveraged in combination with modern deep learning techniques, and can potentially lead to improved drug activity predictions and cheaper and more effective experimental design. However, existing collections of machine-learning-ready public datasets do not exploit the multiple data modalities present in real-world HTS projects. Thus, the largest fraction of experimental measurements, corresponding to hundreds of thousands of “noisy” activity values from primary screening, are effectively ignored in the majority of machine learning models of HTS data. To address these limitations, we introduce Multifidelity PubChem BioAssay (MF-PCBA), a curated collection of 60 datasets that includes two data modalities for each dataset, corresponding to primary and confirmatory screening, an aspect that we call *multifidelity*. Multifidelity data accurately reflect real-world HTS conventions and present a new, challenging task for machine learning: the integration of low- and high-fidelity measurements through molecular representation learning, taking into account the orders-of-magnitude difference in size between the primary and confirmatory screens. Here we detail the steps taken to assemble MF-PCBA in terms of data acquisition from PubChem and the filtering steps required to curate the raw data. We also provide an evaluation of a recent deep-learning-based method for multifidelity integration across the introduced datasets, demonstrating the benefit of leveraging all HTS modalities, and a discussion in terms of the roughness of the molecular activity landscape. In total, MF-PCBA contains over 16.6 million unique molecule–protein interactions. The datasets can be easily assembled by using the source code available at https://github.com/davidbuterez/mf-pcba.

## Introduction

Machine learning (ML) techniques have enabled remarkable progress in the chemical and physical sciences, particularly in terms of fast and precise modeling of computationally expensive processes. Graph neural networks (GNNs), a class of geometric deep learning algorithms, have recently emerged as one of the leading ML paradigms for learning directly on the data types occurring in the life sciences. Thanks to their ability to naturally learn from non-Euclidean data structures, represented as objects (nodes) and their connections (edges), GNNs have the potential to model complex relationships and dependencies between nodes. Successful examples include but are not limited to tasks from particle physics,^1^ simulations of fluid dynamics and other physical systems,^2^ quantum chemistry,^3^ and drug discovery.^4^ However, such data-driven efforts are highly dependent on the quality, quantity, and availability of suitable data. Thus, a significant amount of research has been devoted to the development of high-quality datasets that support the rapidly advancing field of graph representation learning. In particular, challenging computational chemistry tasks such as quantum property prediction, drug-like molecule generation, and few-shot learning have put molecular data at the forefront of geometric deep learning research.

Publicly available molecular benchmarks span several areas of chemistry and are often tailored to domain-specific needs. For example, MoleculeNet is a suite of 16 molecular datasets and their variations, with tasks from quantum mechanics, physical chemistry, biophysics, and physiology.^5^ Consequently, there is a large variability in terms of the supervised learning task (regression or classification), number of output predictions/classes (multitask learning), dataset size (ranging from under 1000 compounds to over 430,000), and ML-specific concerns such as the node featurization strategy (atomic coordinates, atom type, etc.) and the random split strategy. A similarly diverse effort is Atom3D, a collection of eight tasks formulated specifically for learning based on three-dimensional molecular structures, for example, protein-interface prediction and prediction of ligand binding affinity and small-molecule properties.^6^

Experimental and computational advances paved the way to increasingly large, diverse, and challenging datasets. For example, although QM9 is one of the most well-known quantum mechanics benchmarks, concerns regarding its size (around 130,000 compounds) and composition (only five atom types and a maximum of nine heavy atoms per molecule) led to projects such as Alchemy^7^ (up to 14 heavy atoms), PubChemQC^8^ (quantum properties for up to 3 million molecules), QMugs^9^ (over 665,000 diverse molecules, up to 100 heavy atoms per molecule, and quantum properties at multiple levels of theory), and ANI-1x and ANI-1ccx^10^ (quantum properties at different levels of theory for 5 million and 500,000 molecules, respectively). For drug discovery, the well-known PCBA dataset^11^ is a collection of high-quality dose response data, formulated as a multitask learning benchmark from 128 high-throughput screening (HTS) assays. Models trained on experimental data of this nature are often used in a virtual screening setting, with the goal of identifying novel compounds of therapeutic interest. For the specific task of virtual screening, an alternative benchmark named LIT-PCBA was developed,^12^ addressing limitations of existing datasets such as hidden molecular biases.

We identified a lack of machine learning benchmarks that accurately reflect the practical aspects of drug discovery based on HTS, thus also limiting the potential for innovation in early-stage drug discovery projects. HTS follows a multitiered approach consisting of successive screens of drastically varying size and fidelity, most commonly a low-fidelity primary screen consisting of up to 2 million molecules in industrial settings and a high-fidelity confirmatory screen of up to 10,000 compounds. Usually, the screens are used as successive filters to select the most promising molecules for further optimization. Despite the success of HTS in identifying clinical candidates and FDA-approved drugs,^13−15^ computational methods have traditionally neglected the multitiered design of HTS. Existing datasets and applications, such as PCBA, LIT-PCBA, and approaches based on HTS fingerprints, are formulated on single-fidelity data.^11,12,16−19^ The single-fidelity measurements generally correspond to the highest-fidelity measurements available (dose response), which leads either to extremely small datasets for machine learning (<10,000 compounds) or to highly sparse representations extracted from hundreds of assays.

Neglecting the multiple modalities of HTS experiments has the major downside of discarding millions of lower-fidelity measurements that cover an orders-of-magnitude larger and more diverse chemical space. Integrating all of the available data has the potential to improve drug potency predictions, help guide the experimental design, save costs associated with multiple expensive experiments, and ultimately lead to the identification of new drugs. Indeed, we have recently demonstrated considerable uplifts in predictive performance when multifidelity data are incorporated through a novel, specifically designed GNN approach.^20^ We have also demonstrated transfer learning capabilities between the different data modalities, an area that has also recently received interest in quantum machine learning.^3,21^

In this work, we expand upon our initially reported collection of public multifidelity HTS datasets, reporting a total of 60 datasets with over 16.6 million unique protein–molecule interactions extracted from PubChem. We cover in detail the search, selection, and filtering steps and also provide a performance evaluation based on the methodology proposed by Buterez et al.^20^ We envision that the provided datasets will motivate the development of new graph representation learning methods capable of multifidelity modeling and transfer learning, thus advancing the state of the art in computational drug discovery projects. At the same time, we believe that the challenging properties of multifidelity HTS data will provide a new benchmark for evaluating machine learning algorithms, particularly in terms of molecule-level regression tasks, which are not well represented at this scale in existing work. It is important to note that the MF-PCBA datasets are representation-agnostic, as the molecular information is presented in the form of SMILES strings, such that a variety of machine learning techniques are applicable (not exclusively deep learning). Ultimately, we hope that the MF-PCBA datasets will motivate further research and competition, thus advancing the field and enabling new possibilities for drug discovery and molecular modeling. The datasets can easily be assembled using the provided scripts (https://github.com/davidbuterez/mf-pcba).

## Methods

### Data Acquisition

We manually searched the PubChem BioAssays database using the terms “high-throughput screening”, “HTS”, “primary”, “confirmatory”, “single dose”, “dose response”, “SD”, and “DR”. We selected the latest version (at the time of writing) for each assay. The date range of the assays is 2008–2018 (the exact versions, revisions, and dates are provided in the Supporting Information (SI)). The manual search step is necessary due to the nature of reporting HTS results. Primary and confirmatory screening results might be reported in the same bioassay, e.g., assay identifier (AID) 1445, or as separate bioassays that are part of the same project, e.g., primary AID 602261 and confirmatory AID 624326. Furthermore, different primary and confirmatory projects use different formats and conventions when reporting measurements. Among the selected bioassays, primary screens might be reported at different concentrations and with multiple replicates. Similarly, confirmatory screens can be reported in different formats (usually IC_50_, AC_50_, or EC_50_), but the units (e.g., μM or μg/mL) are not always consistent. The smaller collection of 23 datasets previously discussed by Buterez et al. focused on a diverse but restricted number of assays to allow for an in-depth analysis of both PubChem and proprietary data while also emphasizing certain dataset attributes such as the (linear) correlation between the single dose (SD) and dose response (DR) values. The updated collection presented in this work further increases the diversity of the assays and the number of curated datasets, allowing a more comprehensive view of HTS modeling strategies and challenging current methods.

Once our selection of 60 datasets was finished, we could automate the remaining steps. To this end, we used PubChem’s representational state transfer application programming interface (REST API) to retrieve each assay as a comma-separated values (CSV) file based on its AID, downloading at most 10,000 compound rows at a time. The raw CSV files contain the compound ID (CID) for each molecular entry but no molecular structure information. To address this, we again used the REST API to retrieve the Simplified Molecular-Input Line-Entry System (SMILES) string representation for each entry based on the CID. Any replicate measurements present for the primary screens are aggregated by taking the mean. In the associated source code, the option of using the median is also provided. Only 23 out of the 60 primary screens have more than two replicates, and even in these cases only a fraction of the entire compound library is present in the replicates, leading to a small difference between the mean and median (Table S3 and section SI 2). The resulting SD measurements are not further processed. In contrast, for the DR measurements the aggregation step is not necessary, as generally there are no replicates in the same bioassay. However, the DR values, which are often reported as “XC_50_” (IC_50_, AC_50_, or EC_50_) values or variations involving the logarithm, are always converted to the corresponding pXC_50_ values (pXC_50_ = −log XC_50_). The entire collection of 60 multifidelity datasets is summarized in Table 1 along with details specific to each dataset. These datasets cover a wide spectrum of assay technologies and end points, from biochemical inhibition data to phenotypic experiments on whole organisms.

**Table 1:** Summary of the 60 Multifidelity HTS Datasets, Including the PubChem AIDs, Assay Types, SD and DR Measurement Types, Dataset Sizes (Denoted by #), Numbers of DR Compounds Lacking SD Measurements (Denoted by “# DR (no SD)”), the Pearson Correlation Coefficients (*r*) for the Paired SD/DR Measurements, and the Associated *p* Values^a^

SD AID	DR AID	Assay	SD type	DR type	# SD (unf.)	# DR (unf.)	# DR (no SD)	SD/DR *r*	*p* value
1619	–	chem.	inh. @ 30 μM	IC_50_	217,147	827	0	0.47	7.51 × 10^–45^
488975	504840	cell	B-score @ 10 μM	IC_50_	306,595	1544	15	–0.10	5.17 × 10^–3^
2097	434954	chem.	act. @ 10 μM	log EC_50_	302,503	2198	13	–0.35	3.10 × 10^–18^
624330	–	chem.	inh. @ 30 μM	IC_50_	342,291	2057	0	0.66	2.30 × 10^–198^
504558	588343	cell	act. @ 12.5 μM	pAC_50_	345,298	1241	0	0.08	4.75 × 10^–2^
2221	449749	chem.	act. @ 7.5 nL	log AC_50_	293,466	2133	8	0.44	1.42 × 10^–76^
1259416	1259418	cell	act. @ 100 nL	pAC_50_	69,082	3560	189	–0.37	1.97 × 10^–24^
1979	2423	org.	act. @ 7.5 nL	EC_50_	302,509	1838	20	–0.07	7.26 × 10^–3^
2732	504313	cell	inh. @ 10 μM	IC_50_	219,164	940	39	–0.09	5.84 × 10^–3^
2216	435026	chem.	act. @ 100 nL	log EC_50_	302,453	1016	11	0.26	2.04 × 10^–7^
2553	2696	cell	B-score @ 10 μM	EC_50_	305,679	900	3	–0.22	2.07 × 10^–3^
651710	652116	chem.	act. @ 18.71 μM	pAC_50_	355,860	996	4	0.49	5.44 × 10^–23^
652162	720512	chem.	act. @ 9.99 μM	pAC_50_	352,852	931	18	0.62	9.55 × 10^–13^
1903	–	chem.	inh. @ 20 μM	IC_50_	306,015	1203	0	0.26	2.50 × 10^–18^
2099	488835	chem.	act. @ 2.35 μM	log AC_50_	328,736	1413	4	0.26	2.26 × 10^–3^
489030	588524	chem.	act. @ 20 μM	IC_50_	331,760	476	1	0.32	6.73 × 10^–11^
1662	1914	cell	act. @ 7.5 μM	EC_50_	303,545	3266	4	0.09	8.11 × 10^–6^
743445	1053173	chem.	act. @ 12.48 μM	pAC_50_	309,831	1503	2	0.48	1.83 × 10^–30^
2227	434941	cell	B-score @ 10 μM	EC_50_	305,669	2267	11	–0.22	1.11 × 10^–1^
435005	449756	cell	act. @ 100 nL	log AC_50_	303,588	2288	3	0.25	3.59 × 10^–27^
2098	2382	cell	act. @ 7.5 μM	EC_50_	301,406	2448	25	–0.24	1.29 × 10^–29^
2650	463203	chem.	act. @ 10 μM	log AC_50_	315,508	2352	172	0.42	1.83 × 10^–31^
686996	720632	chem.	act. @ 12.48 μM	pAC_50_	347,992	962	46	0.45	1.73 × 10^–21^
873	1431	chem.	inh. @ 5 μM	IC_50_	214,261	1260	0	0.08	8.22 × 10^–3^
652115	720591	chem.	act. @ 14.98 μM	pAC_50_	326,679	1194	65	0.20	8.96 × 10^–3^
504582	540271	org.	act. @ 12.5 μM	pAbsAC_1000_	336,846	826	1	0.19	2.02 × 10^–5^
1259416	1259420	cell	act. @ 100 nL	pAC_50_	69,082	1220	191	–0.28	1.86 × 10^–4^
1117319	1117362	chem.	inh.	IC_50_	262,345	3634	3	–0.10	3.99 × 10^–1^
1445	–	chem.	inh. @ 30 μM	IC_50_	217,157	673	0	0.78	6.06 × 10^–137^
504621	540268	org.	act. @ 9.4 μM	pAC_50_	307,324	930	10	0.10	3.25 × 10^–3^
504408	435004	cell	act. @ 9 μM	log EC_50_	301,246	1953	2	0.34	7.88 × 10^–17^
624304	624474	org.	inh. @ 21.8 μM	IC_50_	364,167	1381	3	0.58	1.43 × 10^–121^
652154	687027	cell	act. @ 12.62 μM	pAC_50_	356,670	1810	109	0.10	1.72 × 10^–3^
720511	743267	cell	act. @ 7.58 μM	pAC_50_	347,956	1170	12	0.01	7.67 × 10^–1^
602261	624326	chem.	act. @ 15 μM	IC_50_	362,387	1011	2	0.68	1.03 × 10^–133^
1224905	1259350	chem.	Z-score (FI @ 535 nm)	FP (mP)	206,863	579	0	0.41	2.11 × 10^–24^
652115	720597	chem.	act. @ 14.98 μM	pAC_50_	326,679	964	103	–0.01	9.49 × 10^–1^
488895	504941	org.	act. @ 7.5 nL	pAC_50_	337,881	1215	2	0.63	4.15 × 10^–19^
493091	540297	chem.	act. @ 20 μM	IC_50_	340,929	1011	3	0.23	1.08 × 10^–12^
2237	434937	cell	B-score @ 10 μM	EC_50_	305,669	2267	5	0.03	4.85 × 10^–1^
504329	–	chem.	inh. @ 12.5 μM	IC_50_	335,445	1010	0	0.79	7.85 × 10^–192^
2221	449750	chem.	act. @ 7.5 nL	log AC_50_	293,466	2133	10	0.29	4.45 × 10^–29^
588489	602259	chem.	act. @ 20 μM	IC_50_	359,520	1186	1	0.37	2.74 × 10^–38^
485317	493248	chem.	act. @ 7.5 nL	pAC_50_	288,803	2345	4	0.25	1.46 × 10^–20^
588549	624273	chem.	act. @ 12.48 μM	pAC_50_	355,325	1047	3	0.70	1.55 × 10^–54^
2247	434942	cell	B-score @ 10 μM	EC_50_	304,070	2267	16	0.01	7.38 × 10^–1^
504558	588398	cell	act. @ 12.5 μM	pAC_50_	345,298	1241	0	0.23	2.29 × 10^–2^
651658	687022	chem.	act. @ 9.99 μM	pAbsAC1	343,072	1025	29	0.02	7.05 × 10^–1^
1832	1960	chem.	act. @ 10 μM	EC_50_	301,856	1691	12	–0.47	2.45 × 10^–89^
2629	435023	chem.	act. @ 7.5 nL	log EC_50_	323,875	1430	1	0.34	1.17 × 10^–3^
1832	1964	chem.	act. @ 10 μM	EC_50_	301,856	1691	10	–0.45	9.01 × 10^–82^
485273	493155	chem.	inh. @ 20 μM	IC_50_	330,481	1210	5	0.58	3.80 × 10^–88^
588689	–	chem.	inh. @ 25 μM	IC_50_	338,853	1013	0	0.51	6.07 × 10^–65^
449762	–	cell	inh. @ 25 μM	IC_50_	327,669	1938	0	0.20	6.04 × 10^–18^
488899	493073	cell	act. @ 100 nL	pAC_50_	331,578	1241	17	0.23	5.40 × 10^–10^
2221	435010	chem.	act. @ 7.5 nL	log EC_50_	293,466	2133	9	0.56	1.27 × 10^–149^
1465	–	org.	fold ind. @ 50 μM	EC_50_	215,402	159	0	0.05	5.42 × 10^–1^
1949	–	cell	inh. @ 10 μg/mL	IC_50_	100,697	1688	5	–0.06	2.20 × 10^–2^
449739	489005	cell	B-score @ 10 μM	log EC_50_	104,742	895	1	–0.30	1.80 × 10^–15^
1259374	1259375	chem.	inh. @ 2.6 μM	log IC_50_	646,073	474	10	0.10	6.89 × 10^–2^

a If the confirmatory data are available separately, both AID columns are populated; otherwise, the SD dataset includes the DR data. Abbreviations: inh., inhibition; act., activation; ind., induction; FP, fluorescence polarization; FI, fluorescence intensity; unf., unfiltered.; chem., chemical; org., organism.

### Filtering Steps

**Figure 1:**
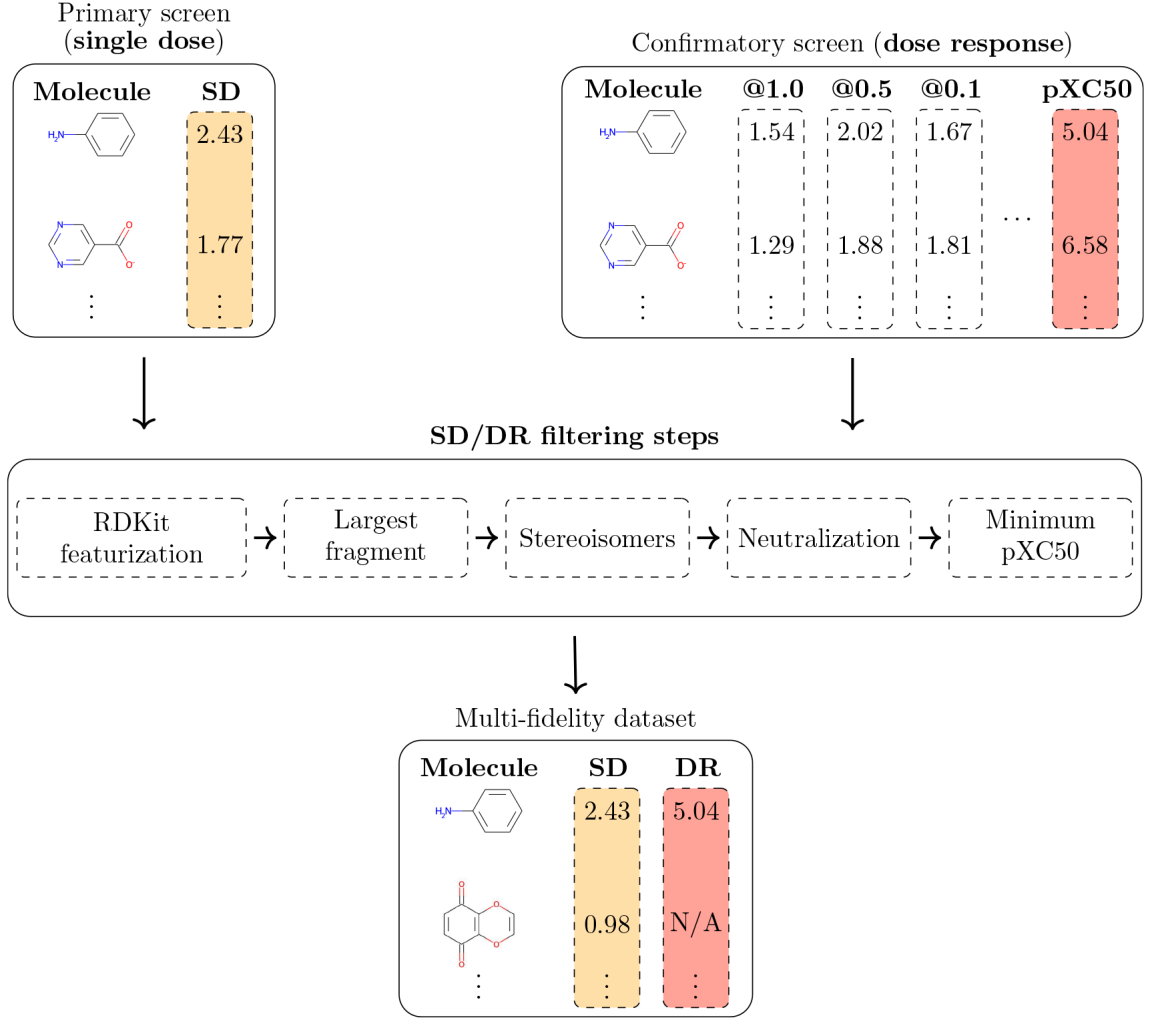
Main steps of assembling a multifidelity HTS dataset from the corresponding primary and confirmatory screens, which might be reported in the same or different PubChem assays. First, the SMILES string for each molecule is loaded into RDKit, which removes invalid structures. This procedure is followed by a number of filtering steps that ensure a high-quality data collection for drug discovery and machine learning projects, including selection of only the largest fragments, removal of stereoisomers, and removal of electric charges. Finally, missing DR values in the confirmatory assay are set to a default value corresponding to the minimum activity (“pXC_50_”) observed in the project.

The raw molecular information is subjected to a number of filtering steps that ensure a high-quality, consistent representation suitable for various machine learning algorithms. In the majority of cases, the primary and confirmatory screens are reported as separate bioassays in PubChem. Thus, the filtering steps are applied independently on the SD and DR datasets. In the case where the primary and confirmatory data are reported in a single bioassay, the same filtering steps are applied on the single data table. Filtering is always applied after the preprocessing steps described above. The filtering pipeline consists of four major steps implemented using the open-source library RDKit^22^ (also illustrated in Figure 1):

1. **Sanitization with RDKit:** The function Chem.MolFromSmiles from RDKit was used to construct a molecular object, represented by the Mol class. This representation provides access to the molecular structure and to other filtering-relevant functions regarding stereoisomers, removal of charges, etc. Importantly, loading the SMILES string into RDKit acts as an initial filtering step by itself, as RDKit uses a sanitization procedure internally. This resulted in a number of entries (molecules) being removed, most commonly due to valence errors or SMILES strings consisting only of hydrogen atoms.
2. **Largest fragment selection:** Next, we used RDKit’s rdMolStandardize.LargestFragmentChooser function to remove molecules where the CID contained a combination of compounds, most commonly a counterion. The option to remove smaller fragments but keep the largest one is also provided in the source code. There is also an option to report small fragments that are not encountered in the MF-PCBA collection when assembling data from different assays. For MF-PCBA, almost all encountered small fragments are counterions or solvents.
3. **Stereoisomer removal:** The function rdmolops.RemoveStereochemistry was used to remove all of the stereochemistry information contained within the molecules, followed by generation of new SMILES representations for all of the resulting molecular objects. Molecules that were previously differentiated only by the stereochemical information now have identical representations. Thus, we select only the unique molecules based on the SMILES representation and consider the task of modeling the activity of racemic mixtures. It should be noted that many GNNs operate on molecules only at the level of “2D” graphs, such that the stereochemical information is not considered. An option to keep the molecules that would be removed by this step is also provided, as well as an option to record the number of stereocenters for each molecule.
4. **Neutralization (removal of electric charges):** This step is achieved by adding or removing hydrogens for charged atoms. Only compounds that can be recorded with a formal charge of 0 are retained. Generally, compounds that cannot pass this threshold are difficult to transform into safe drugs, motivating our filtering procedure. The calculations are performed using atom-level RDKit functions such as GetFormalCharge, GetTotalNumHs, SetFormalCharge, SetNumExplicitHs, and the filtering of molecules is based on rdmolops.GetFormalCharge.

We report the average number of compounds removed by each step in the Results. The numbers of SD and DR compounds after each filtering step are reported for all datasets in Tables S1 and S2, respectively. Generally, the filtering steps that removed the most compounds are largest fragment selection and stereoisomer removal. With a few exceptions, the filtering pipeline did not radically change the size of the dataset. However, one important issue was observed for the DR datasets that originated in different PubChem bioassays. Although the confirmatory bioassays contain partial experimental measurements for all of the compounds listed in Table S2, actual DR values such as the IC_50_ are often reported only for a fraction of the dataset, for example, only for active compounds. Although some DR datasets do not suffer from this issue (e.g., AID 1445), others are at the other extreme of the spectrum, with up to 97.8% of filtered compounds lacking explicit XC_50_ measurements (AID 1117362). On average, 35.57% of compounds do not possess experimentally derived XC_50_ labels because they were insufficiently active at the top concentration. To address this limitation, we associated the compounds missing this information with the lowest activity value recorded in each dataset (“Minimum pXC_50_” in Figure 1), providing a training target for machine learning models. This is done to provide a consistent value for inactive molecules and should be interpreted as a lower bound on the pXC_50_ value. We maintain a flag for each molecule indicating whether its confirmatory activity was experimentally derived or set to a default value.

### Implementation

The data acquisition and filtering steps are implemented as a Python script (pubchem_retrieve.py) with a simple command-line user interface. The program allows downloading and filtering all 60 MF-PCBA datasets as well as any other PubChem assays with similar formats. The main arguments are --AID, indicating the AID of a PubChem assay corresponding to an SD experiment; --AID_DR, optionally indicating the AID of a PubChem assay corresponding to a DR experiment that is separate from the SD dataset; --list_of_sd_cols, indicating the names of the SD activity value columns (including replicates) as displayed on PubChem; --list_of_dr_cols, indicating the names of the DR activity value columns; and transform_dr, allowing the conversion to the corresponding pXC_50_ value. More advanced filtering options are documented in the source code. The GitHub repository contains customized scripts (i.e., with all of the arguments already set) for all 60 MF-PCBA datasets, such that they can be simply downloaded and assembled with a single call to the intended script. Once downloaded and filtered, the DR datasets can be split into train, validation, and test sets according to the provided five random seeds, enabling easy comparison to the results obtained by previous work and in this article. Splitting is demonstrated with an interactive notebook that is part of the source code.

### MF-PCBA Format and Problem Specification

After filtering, each of the 60 multifidelity datasets contains the PubChem CID for each molecule, the corresponding molecular structure (SMILES), and the SD and DR activity values. If the SD and DR data originated from the same bioassay, both the SD and DR modalities are provided in the same CSV file. In this case, the name of the multifidelity dataset is given by the original assay name, e.g., AID 1445. Otherwise, separate SD and DR files are provided, and the naming scheme reflects the two data sources, with the DR dataset first (e.g., AID 624326 – 602261).

One of the intended purposes of MF-PCBA is to enable high-quality confirmatory-level predictions by modeling of DR data. Thus, the prediction problem can be naturally formulated as a supervised regression task. At the same time, the binary activity labels from PubChem are included for each dataset. Large amounts of SD activity values are available to support the goal of multifidelity integration for improving the quality of confirmatory-level predictions. Furthermore, we provide five random splits per dataset (80%/10%/10% split ratios for the train/validation/test sets) for the DR data. The accompanying source code allows easy conversion of the CSV files obtained previously into individual train/validation/test CSV files corresponding to each random split seed. A wide range of machine learning techniques are applicable on MF-PCBA, including any algorithm that operates on fixed-dimension vector representations (molecular fingerprints, physical and chemical descriptors, atom and bond encodings, etc.).

As the MF-PCBA collection is based entirely on real-world data generated by HTS experiments, it enables the design and evaluation of new machine learning architectures specifically for drug discovery. Additionally, MF-PCBA can be used as a suite of benchmarks for evaluating the performance of newly developed machine learning applications. For example, MF-PCBA can support the rapidly developing field of representation learning with graph neural networks, particularly in terms of graph-level regression tasks, which are currently underrepresented. At the same time, the collection offers a unique challenge in the form of multifidelity (multimodality) integration. This is a characteristic of drug discovery by HTS that presents new opportunities for computational modeling and ultimately for improved experimental and hybrid wet-lab and in silico workflows. We also envision that the data curated here can be used for other modern machine learning techniques such as generative approaches and few-shot learning.

### Assay Overlap and Size Considerations

Since PubChem and ChEMBL^23,24^ are frequently used as data sources for machine learning, it is important to fully characterize MF-PCBA in terms of size, scope, and intended applications. Although many previous studies considered drug bioaffinity data (the ChEMBL 20-derived subset of Mayr et al.,^25^ PCBA,^11^ LIT-PCBA,^12^ and FS-Mol^26^ are just a few examples), to the best of our knowledge no previous work explicitly considered HTS data of different fidelities or proposed transfer learning in this context. Despite not being purposefully designed to be orthogonal to existing datasets, MF-PCBA shares no overlap in terms of common assays with the 128 assays of PCBA. Twenty-seven of the confirmatory assays that we use are also present in the 1310 assays reported by Mayr et al. There is no overlap in terms of the primary screening assays. We would like to note that a small level of overlap is expected considering that Mayr et al. focused on a large number of generally confirmatory assays extracted from the ChEMBL 20 database. Furthermore, our motivation and our preprocessing and evaluation pipelines are different.

In terms of size, MF-PCBA consists of over 16.6 million unique compound–target interactions, with over 1.1 million unique compounds. In contrast, the largest dataset based on the number of compounds that is reported in the comparison provided by the FS-Mol study has 955,386 compounds. The number of measurements is also larger than in FS-Mol and LSC.^27^ Moreover, rigorously tagged primary and confirmatory data are more limited compared to other data types. An example search through PubChem returns 407 confirmatory DR datasets (the search steps are described in section SI 10). However, not all of them qualify for MF-PCBA. For example, some assays study toxicity effects or are missing an associated primary screen. As such, some degree of manual selection is required to satisfy all of the previously defined criteria. Overall, the MF-PCBA collection aims to provide a diverse set of multifidelity datasets that is easily extensible through the open source implementation.

Finally, we note that we currently only consider single-task learning, such that a separate model is trained for each of the datasets present in MF-PCBA. This also has the advantage of enabling the use of a wide range of machine learning algorithms other than deep learning. While multitask learning is a common occurrence in chemical modeling projects (e.g., PCBA, FS-Mol), it is not one of the main objectives for MF-PCBA. Since the data uploaded to PubChem originate from various groups using different compound libraries and protocols, the overlap between the chosen assays in terms of common compounds is limited. If we associate all of the unique compounds of MF-PCBA with their activities in the primary screens (or NaN if a compound is not screened in the assay), we find that over 75.6% of values are NaNs. For confirmatory screens, this grows to over 97.7%. Previous work on HTS fingerprints assigned a default activity value (usually 0) to all missing values. Since the true activity is not known, this would lead to a multitask learning scenario where over two-thirds of the data have false values. Sturm et al.^19^ succeeded in assembling a collection of only 57,124 molecular fingerprints with reduced sparsity, despite starting with an industrial collection of 1250 HTS assays that are generally expected to use the same or similar compound libraries. However, multitask learning for a subset of MF-PCBA with a larger overlap could be an interesting direction for future research.

### Evaluation Strategies and Multifidelity Modeling

**Figure 2:**
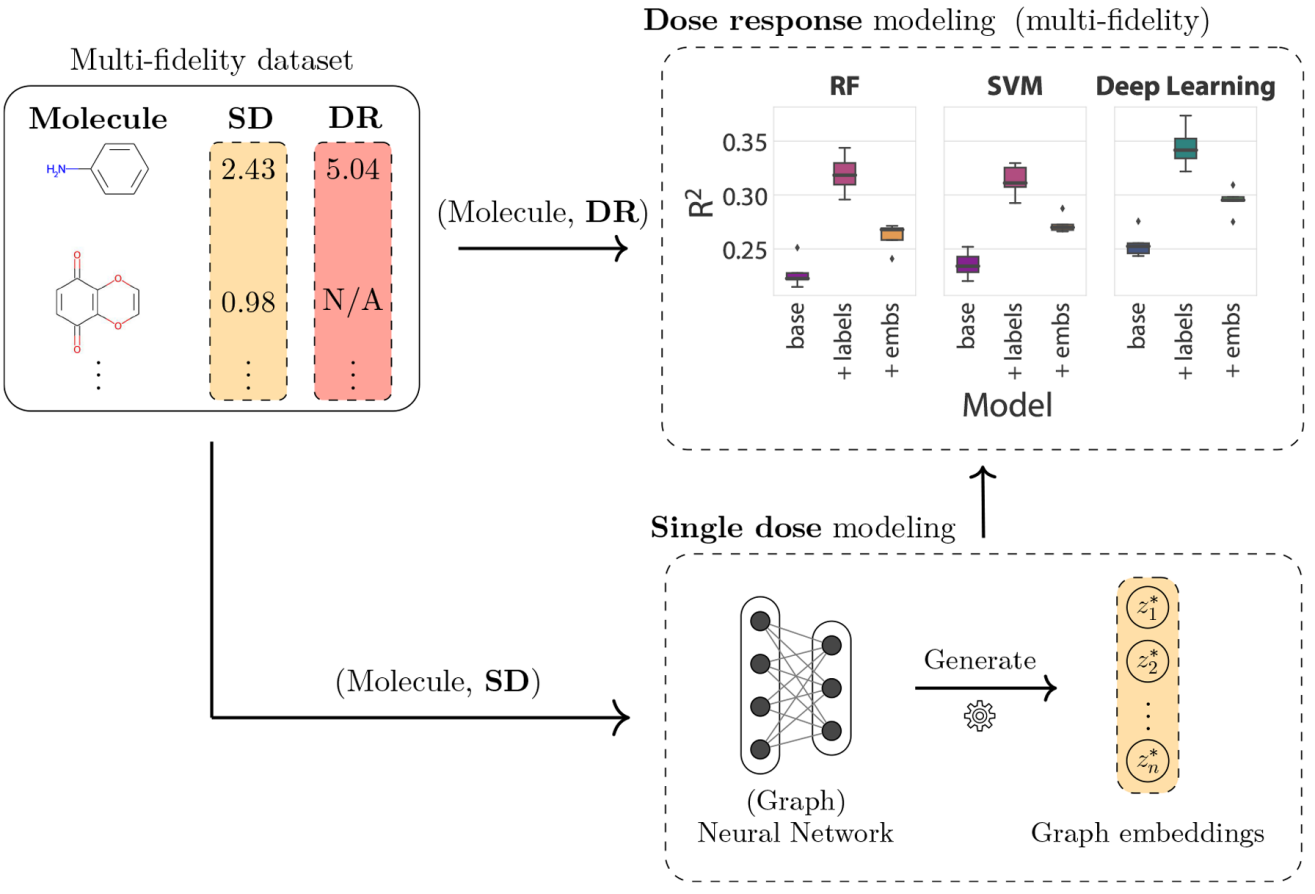
A multifidelity integration workflow illustrated with three high-level steps. Confirmatory-level data (DR) can be modeled directly using various machine learning techniques such as RF, SVM, or GNN. Despite not leveraging hundreds of thousands of bioaffinity measurements from the primary screens, this is the prevalent type of modeling in early-stage drug discovery. Instead, an alternative is to model the primary screening (SD) separately and incorporate the learned information through techniques such as transfer learning. Here, this is achieved through *graph* or *molecular* embeddings. The embeddings can be included (by concatenation) in the DR modeling step, a procedure we denote as *augmentation*. We also consider the addition of the SD label to the DR model as an alternative augmentation strategy.

As a first step, we provide an evaluation of the multifidelity integration performance for all 60 datasets based on the multifidelity modeling workflow recently proposed by Buterez et al.^20,28^ (Figure 2). In this context, the machine learning task is formulated as a supervised learning problem on confirmatory data, i.e., molecules with DR measurements. Thus, the simplest task is training and evaluating models exclusively on data points with DR labels, corresponding to training sets with typically less than 2000 instances, and ignoring the orders-of-magnitude larger SD datasets. Multifidelity modeling is achieved through two *augmentation* strategies that integrate information derived from the SD data. The first augmentation corresponds to the inclusion of the experimentally determined SD activity value in the internal representation (fixed-dimension vector) of each compound in a machine learning model, corresponding to an append/concatenation operation. All compounds that possess DR activity values typically have an associated SD value, making this strategy generally applicable to HTS datasets. The second augmentation requires a separate machine learning model (here, a graph neural network) to be trained on the entirety of the available SD data in a supervised fashion. In this process, the GNN learns to produce SD *embeddings*, low-dimensional vector representations that carry the molecular information and its SD activity. The resulting SD embeddings can be appended to the internal representation of a machine learning model exactly as for the first augmentation, with the important advantage that the embeddings can be produced for molecules that were not seen during training. Both augmentations are applicable to any machine learning method operating on fixed-dimension vectors.

The evaluation includes three machine learning algorithms for DR modeling: random forests (RF), support vector machines (SVM), and deep learning (GNNs). Separately, we use GNNs for the SD modeling step. The same hyperparameter optimization strategy and the same steps for determining the most suitable deep learning architecture as in the aforementioned study were are also applied. When designing models for MF-PCBA, we recommend evaluating in terms of “DR only” models, which are trained exclusively on confirmatory activity data, and “SD + DR” models that integrate the two modalities. In terms of performance metrics, we recommend the use of *R*^2^ coupled with an error metric such as the mean absolute error (MAE) or root-mean-square error (RMSE) for regression tasks and the Matthews correlation coefficient (MCC) coupled with the area under the receiver operating characteristic curve (AUROC) for classification. The choice is based on both recent recommendations from the literature^29,30^ and the nature of the data.

## Results

In this section, we first report the effect of the filtering steps with regard to the number of removed compounds. We then discuss one of the intended uses of the MF-PCBA datasets by evaluating multifidelity integration machine learning techniques. We use the same modeling techniques previously proposed by Buterez et al., focusing on the analysis of the additional 37 datasets that complete the collection of 60 datasets, as the remaining 23 have already been extensively investigated. In order to highlight meaningful differences between the initial collection of 23 multifidelity datasets and MF-PCBA, we introduce the following naming conventions: the terms MF-PCBA and MF-PCBA-60 are used interchangeably to refer to the entire collection of 60 multifidelity datasets; MF-PCBA-23 refers to the previously analyzed subset;^20^ and MF-PCBA-37 refers to the remaining 37 datasets that complete the collection of 60 datasets. Thus, MF-PCBA-23 and MF-PCBA-37 are disjoint.

### Effects of Filtering on Dataset Size

1. **Sanitization with RDKit:** On average, this step led to the removal of 863 ± 1313.8 (average ± standard deviation) compounds for the SD datasets (0.33% of the unfiltered size) and 8.7 ± 42.7 for the DR datasets (0.46% of the unfiltered size). For the DR datasets, more than 10 compounds were removed only for two datasets.
2. **Largest fragment selection:** After this step, 11207.9 ± 3594.3 compounds were removed for the SD datasets (3.62% of the quantity at the previous filtering step) and 115.3 ± 110.3 for the DR datasets (7.83% compared to the previous step).
3. **Stereoisomer removal:** After this step, 11396.3 ± 23859.4 compounds were removed for the SD datasets (3.78% of the quantity at the previous filtering step) and 30.7 ± 92.3 for the DR datasets (2.49% compared to the previous step). We choose to remove this information because stereochemical annotations are not consistently recorded and not effectively exploited by many baseline methods.
4. **Neutralization (removal of electric charges):** After this step, 396.6 ± 94.4 compounds were removed for the SD datasets (0.15% of the quantity at the previous filtering step) and 3.9 ± 5.1 for the DR datasets (0.31% compared to the previous step).

### Integration of SD Information Increases Predictive Performance

Previously it was shown that the integration of primary screening information leads to increased performance for the *augmented* machine learning models. At the same time, many of the datasets that exhibited considerable improvements by multifidelity integration also possessed certain qualities such as high correlation between the SD and DR experimental measurements.

**Figure 3:**
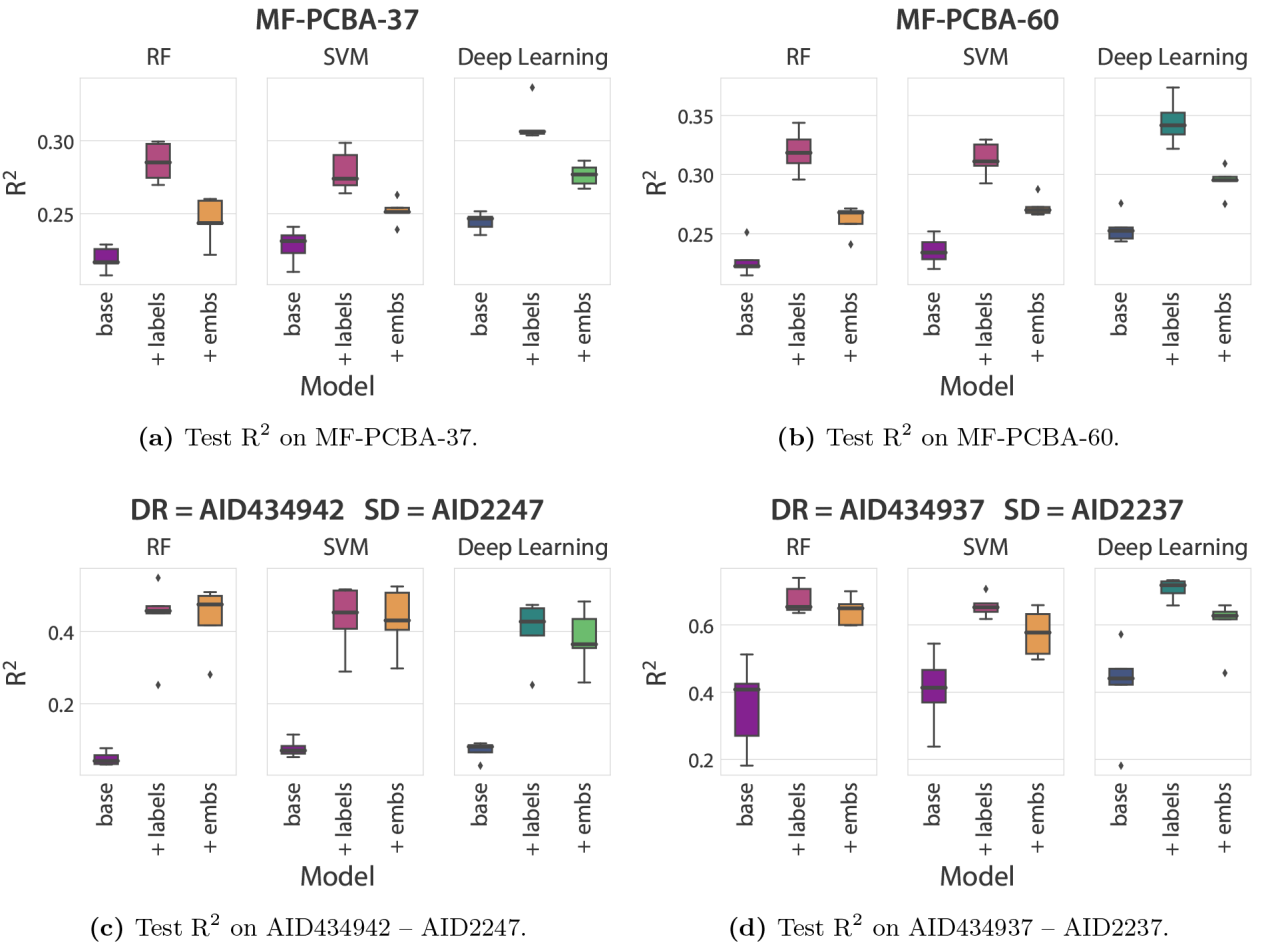
Predictive performance, as measured by *R*^2^, on (a) MF-PCBA-37 (the subset of 37 multifidelity datasets not discussed by Buterez et al.), (b) MF-PCBA-60 (the entire collection of 60 multifidelity datasets), and (c, d) two examples of individual multifidelity datasets from the MF-PCBA-37 subset. The results in (a) and (b) aggregate metrics from each individual dataset. For each dataset, results are reported for augmented and nonaugmented RF, SVM, and deep learning models. Each model was trained multiple times based on the five different random splits for each dataset. For (a) and (b), the means across five different random splits for each dataset are used to generate box plots.

It is thus encouraging to observe that we are able to obtain similar uplifts in predictive performance for the augmented models on the previously unanalyzed 37 multifidelity datasets (Figure 3a). More specifically, the augmentation with SD labels leads to uplifts in *R*^2^, on average, from 0.219 to 0.285 for RF, from 0.228 to 0.279 for SVM, and from 0.244 to 0.312 for deep learning. Similarly, for the SD embeddings augmentation, we observe uplifts in *R*^2^, on average, from 0.219 to 0.245 for RF, from 0.228 to 0.252 for SVM, and from 0.244 to 0.277 deep learning. This lower performance compared to the SD labels augmentation should be balanced against its unique ability among these methods to produce predictions for unobserved molecules.

As for MF-PCBA-23, we encounter several datasets that benefit drastically from the augmentation strategies. For example, on AID2247 – AID434942 (Figure 3c), an uplift from 0.048 to 0.435 in average *R*^2^ is seen for RF with the SD labels augmentation, and an almost identical uplift to 0.436 is achieved for the SD embeddings augmentation. In this particular case, the SD embeddings augmentation is also substantially more effective in reducing the MAE compared to the nonaugmented and SD-labels-augmented models, by almost halving it (section SI 2). Large uplifts are also observed for other datasets such as AID2237 – AID434937 (Figure 3). We also provide an alternative metric in the form of the “unexplained” variance (1 – *R*^2^) in section SI 3.

On average, and compared to the previously studied 23 multifidelity datasets, we generally observe both lower nonaugmented and augmented performance. In particular, the average *R*^2^ for the nonaugmented models on MF-PCBA-23 is 0.253, while for MF-PCBA-37 the observed value is 0.230. The average *R*^2^ achieved by augmenting with SD labels on MF-PCBA-23 was 0.380, compared with 0.291 on MF-PCBA-37. Finally, augmenting with SD embeddings led to an average *R*^2^ of 0.306 on MF-PCBA-23 and 0.257 on MF-PCBA-37. The lower overall performance on MF-PCBA-37 could be explained by lower SD/DR correlation or a notion of dataset difficulty, ideas that are explored further below.

We also studied a variation of the SD labels augmentation where for each compound we computed an “SD fingerprint” (SD FP), a vector consisting of all primary screening activities or 0 if such data are unavailable. The SD fingerprint is used in place of the SD label, similar to an SD embedding. We evaluated this variation using deep learning models on MF-PCBA-60. On the whole, the SD FP augmentation only marginally improved upon the baseline models and severely underperformed compared to the SD labels augmentation (section SI 11). This is not unexpected considering the low compound overlap between assays in MF-PCBA and the low chance of the assay targets being related. When considering individual dataset performance, the SD FP augmentation often performed worse than the baseline, alternatively matching the SD labels performance and rarely improving upon it (section SI 12). We did notice that in seven out of 60 cases the SD-FP augmentation outperformed the SD labels augmentation: AID720632 – AID686996 (Figure S53), AID1465 (Figure S67), AID2382 – AID2098 (Figure S77), AID434954 – AID2097 (Figure S82), AID743267 – AID720511 (Figure S84), AID435004 – AID504408 (Figure S92), and AID463203 – AID2650 (Figure S99). Although this augmentation does not offer consistent performance, it is an interesting alternative for poorly performing datasets like AID1465.

To validate the choice of using average *R*^2^ scores as part of the evaluation procedure (e.g., as the basis of Figure 3), we computed the average *R*^2^ across the five random splits for each dataset and considered nine groups of 60 scores each (corresponding to the 60 datasets). The nine different groups correspond to the three different ML algorithms (RF, SVM, and deep learning) and the three different augmentations (base models, augmented with SD labels, and augmented with SD embeddings). We performed paired *t* tests between each group of augmented scores and the baseline scores (for each ML algorithm), for six tests in total. The tests indicated statistically significant results for all SD label augmentation groups (*p* < 0.0001) as well as for the SD embeddings (*p* < 0.01).

### Relationship between the Predictive Performance and Dataset Attributes

Previous work on multifidelity modeling found the correlation between the SD/DR measurements to be a good indicator of model performance and of possible uplifts.^20^ Here we also performed an analysis of Δ*R*^2^ (the difference in performance between augmented models that perform multifidelity integration of SD and DR and nonaugmented DR-only models) as an indicator of the gains possible through augmentation, using both Pearson’s correlation coefficient and multiple linear regression models, for MF-PCBA-37 and MF-PCBA-60.

**Figure 4:**
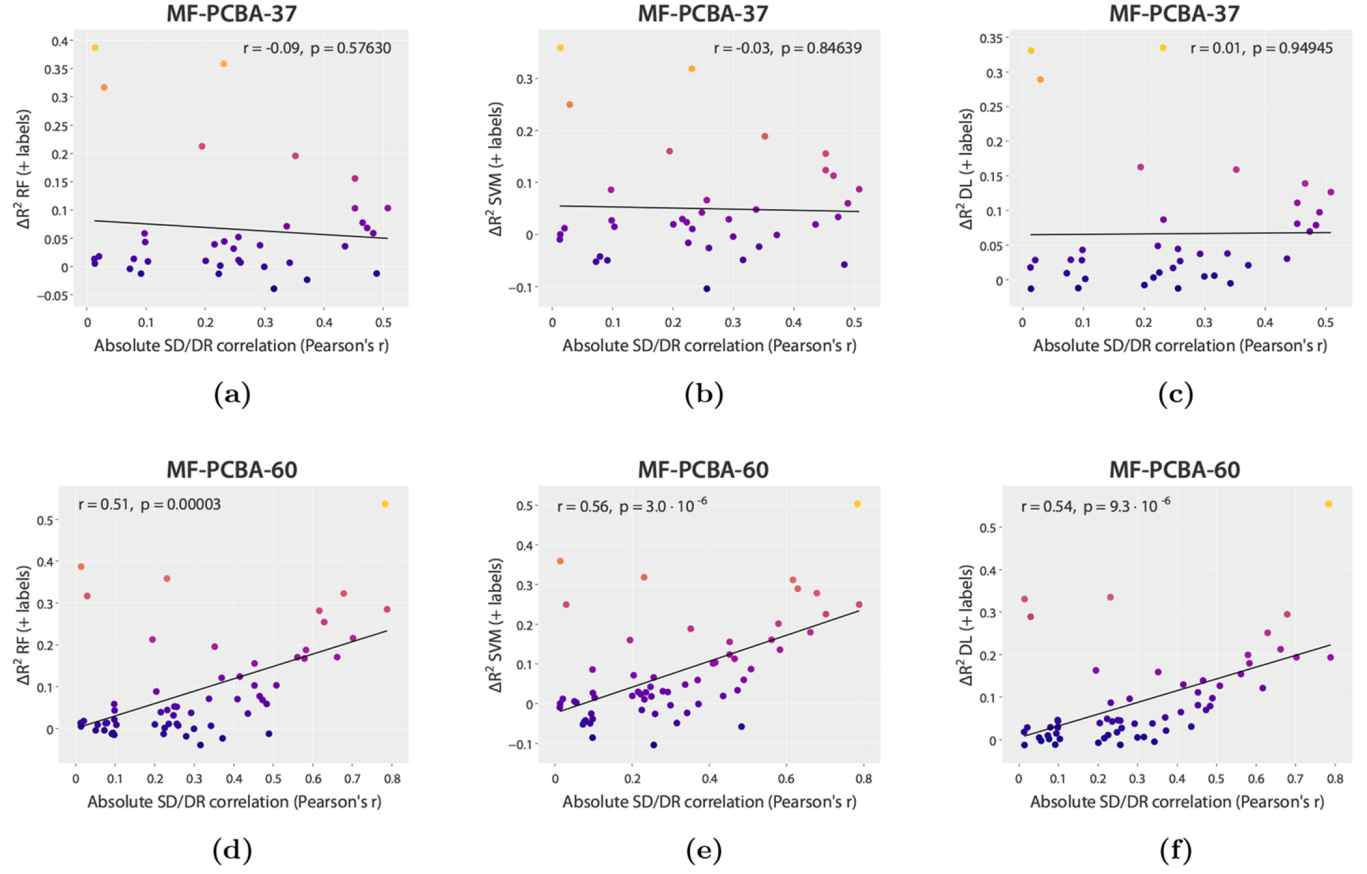
Plots of the absolute SD/DR correlation (*x* axis) against Δ*R*^2^ (*y* axis) for RF, SVM, and deep learning models augmented with SD labels on MF-PCBA-37 and MF-PCBA-60, with the Pearson correlation coefficient (*r*). Brighter colors correspond to higher Δ*R*^2^ values. Plots for the SD embeddings augmentation are available in section SI 4.

On MF-PCBA-37, no linear correlation is observed between Δ*R*^2^ and the SD/DR correlation for all three machine learning algorithms when augmenting with SD labels (Figure 4a–c) and when augmenting with SD embeddings (Figures S45a–c). However, on MF-PCBA-60 a moderate positive relationship is still present (Figures 4d–f and S45d–f). Interestingly, this indicates that the level of SD/DR correlation is not always indicative of the performance uplift, as some of the highest gains seen on MF-PCBA-37 were for datasets with extremely low correlation (e.g., AID434942 – AID2247 and AID434937 – AID2237). Multiple linear regression models did not indicate significant contributions from any other dataset attribute other than the SD/DR correlation, such as the number of DR compounds or the number of SD compounds for MF-PCBA-60 (Tables S7–S12).

### Evaluating the Difficulty of Learning with the Roughness Index

The “roughness” of the molecular activity landscape can provide insights into the level of difficulty that machine learning models are expected to encounter and also reveal challenging dataset characteristics such as activity cliffs. To quantify this property, we use the recently introduced roughness index (ROGI),^31^ which can be applied only to the DR datasets because its computation scales quadratically with the dataset size. Intuitively, the ROGI works by coarse-graining a molecular dataset at different levels and quantifying the change in dispersion, which might be affected rapidly or slowly. The notion of dispersion is quantified using the standard deviation of the molecular property. This is related to the idea that similar molecules with extremely different properties (e.g., activity) lead to rapidly changing dispersion.

We computed the ROGI based on the 208 PhysChem descriptors available in RDKit as of October 2022 (the rdkit.ML.Descriptors.MoleculeDescriptors.MolecularDescriptorCalculator function). The alternative, based on Morgan fingerprints also computed by RDKit, led to small ROGI values for all MF-PCBA datasets. Furthermore, the ROGI computation is applied to the filtered datasets *before* setting missing DR values to the minimum pXC_50_ value. This prevents artificially modifying the ROGI, for example by “flattening” the activity space. In contrast, all of the trained machine learning models use the entire set of DR values, including the minimum defaults, to exploit the entire amount of data available.

**Figure 5:**
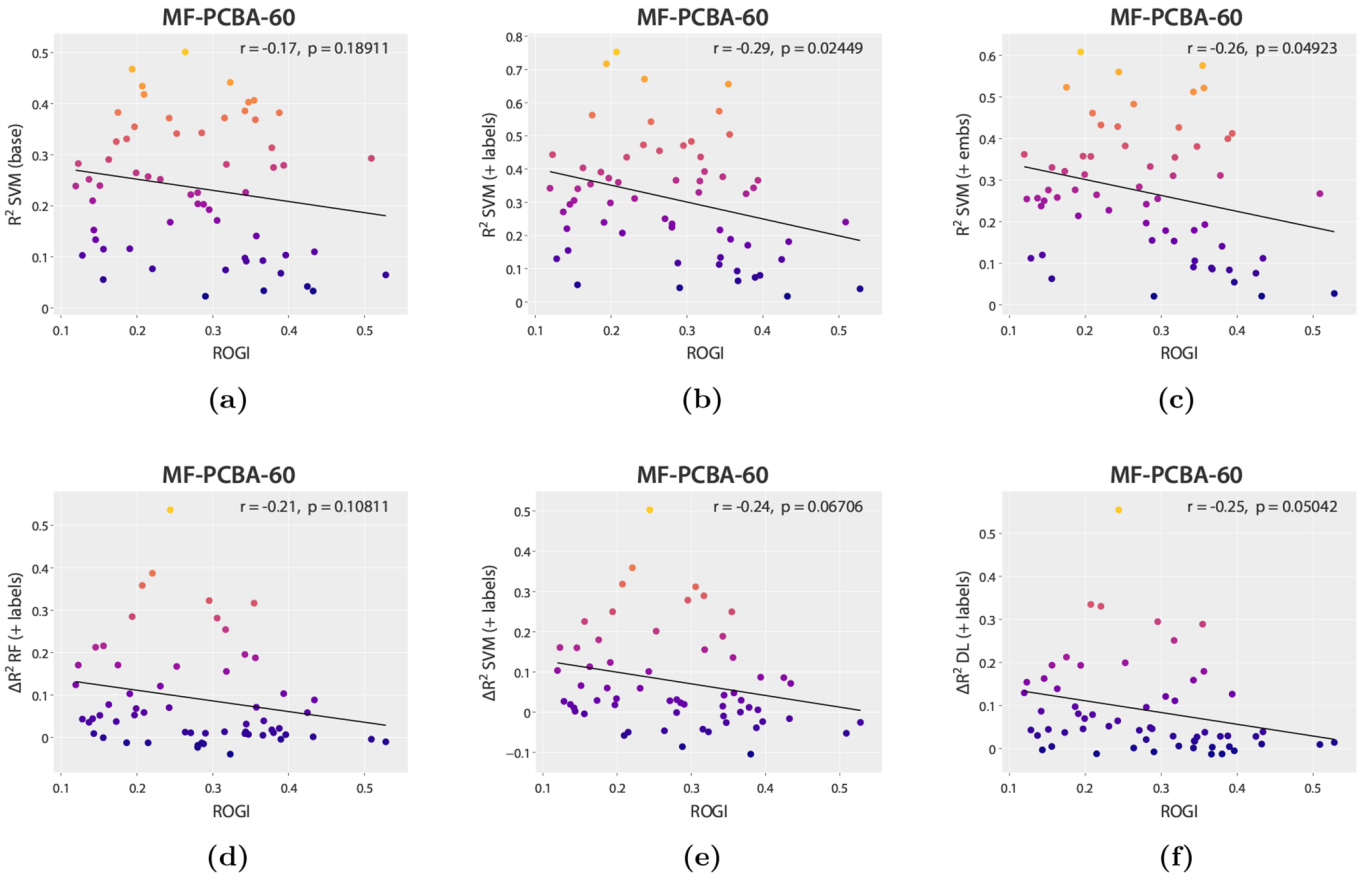
(a–c) Plots of the ROGI (*x* axis) against *R*^2^ (*y* axis) for nonaugmented (base), SD-labels-augmented, and SD-embeddings-augmented SVM models. (d–f) Plots of the ROGI (*x* axis) against the Δ*R*^2^ (*y* axis) for RF, SVM, and deep learning models augmented with SD labels. All panels include the Pearson correlation coefficient (*r*). Brighter colors correspond to higher *R*^2^ or Δ*R*^2^ values. Plots for the rest of the models are available in sections SI 5 and SI 6.

In general, the datasets introduced in the MF-PCBA-60 collection do not appear difficult in terms of the roughness of the chemical space, with ROGI values ranging from 0.014 to 0.571, with an average ± standard deviation of 0.167 ± 0.135 and most of the datasets scoring below 0.4 (see Figure S48 for a histogram). The performance metric (*R*^2^) is not correlated with the ROGI for the RF, SVM, and deep learning nonaugmented (base) models (Figures 5a and S46a,c). However, it is possible to observe statistically significant weak, negative linear correlations for the augmented RF and SVM models (Figures 5b,c and S46d,g). The relationship between the ROGI and Δ*R*^2^ is not significant for any model at a significance level of 0.05 (Figures 5d,f and S47d–f), but in the case of SVM and deep learning the *p* values are close, especially in the case of deep learning (*p* = 0.05042). The highest uplifts in performance are observed for datasets with relatively low ROGI (<0.3), with extremely small or even negative performance differences for the highest difficulty datasets (ROGI > 0.5). Finally, multiple linear regression models did not indicate significant contributions from the ROGI (section SI 9).

## Discussion

We have presented MF-PCBA, a new collection of 60 multifidelity molecular datasets that reflect the real-world nature of HTS projects in drug discovery. With over 16.6 million unique protein–molecule interactions in total, MF-PCBA represents one of the largest collections of graph-level regression datasets, with the possibility to easily adapt to classification. The multifidelity aspect, enabled by the inclusion of primary and confirmatory screening data, is underrepresented in existing molecular modeling efforts, most likely due to uncertainties regarding the noisiness of the data and the vastly different numbers of compounds between the primary and confirmatory screens. Here we have further validated the value of utilizing multifidelity bioactivity measurements, for example by observing uplifts in *R*^2^, on average, between 33% and 40% when using SD labels and depending on the algorithm, further supporting the need for an effective and public multifidelity benchmark, as provided here. Moreover, we have shown that while the SD/DR correlation is a factor in the level of benefit gained by using multifidelity modeling, it is not as strong a factor as had previously been thought.

We showed some tentative signs that the roughness of the activity landscape, as measured by the ROGI, might be another factor, but further work is needed to clarify this and identify additional factors in similar or larger collections. As shown previously, multifidelity modeling can also lead to other desirable properties such as more selective models and new candidate selection workflows. Thus, we envision multifidelity data and models to be a natural step forward for early-stage drug discovery projects based on high-throughput screening. To support this vision, the 60 MF-PCBA datasets are available publicly, aiming to capture the heterogeneity of HTS campaigns in terms of, for example, assay type, targets, screening technologies, primary and confirmatory screen sizes, concentrations, and scoring metrics. Moreover, due to the challenging nature of multifidelity integration, MF-PCBA can act as a representation learning benchmark for future machine learning research and, in particular, for the rapidly advancing area of graph neural networks. At the same time, MF-PCBA is not limited to deep learning, as a multitude of existing machine learning techniques can be applied, enabling more varied comparisons. Overall, we believe that the presented datasets can be used as a basis for advancing both computational drug discovery efforts and molecular machine learning techniques.

## Data Availability

The source code to download, filter, and assemble all 60 MF-PCBA datasets (individually) is available at https://github.com/davidbuterez/mf-pcba. The run time for AID 504329 (335,445 SD molecules) is 554.52 s (9 min 15 s) on a workstation equipped with an AMD Ryzen 5950X processor with 16 cores and 64 GB of DDR4 RAM running Ubuntu 21.10 with an Internet connection speed of 85.81 Mbps (download).

## Supporting Information Available

Molecule counts after each filtering step and distributions of primary screens for each dataset in Table 1, extended version of Figure 3 including metrics for all datasets, Pearson correlation between evaluation metrics and dataset attributes, multiple linear regression results between evaluation metrics and dataset attributes, and augmentation results for primary screening fingerprints (PDF)
