# Supplementary material for: MF-PCBA: Multifidelity High-Throughput Screening Benchmarks for Drug Discovery and Machine Learning
Source: J Chem Inf Model. 2023 Apr 14;63(9):2667–78. doi: 10.1021/acs.jcim.2c01569 (PMC10170507; doi:10.1021/acs.jcim.2c01569)
Supplement: Supplementary file 1 — ci2c01569_si_001.pdf [file ci2c01569_si_001.pdf]

MF-PCBA: Multi-fidelity high-throughput screening benchmarks for drug discovery and machine learning – Supporting Information

David Buterez[1], Jon Paul Janet[2], Steven J. Kiddle[3], and Pietro Liò[1]

[1]Department of Computer Science and Technology, University of Cambridge, Cambridge, CB3 0FD, UK
[2]Molecular AI, Discovery Sciences, R&D, AstraZeneca, Gothenburg, 431 50, Sweden
[3]Data Science & Advanced Analytics, Data Science & Artificial Intelligence, R&D, AstraZeneca, Cambridge, CB2 8PA, UK

SI 1 Molecule count after each filtering step (SD and DR) and SD replicate counts

The dataset size (molecule count) after each of the four major filtering steps described in *Methods – Filtering steps* is listed in Tables S1 and S2 for the SD and DR datasets, respectively. The number of compounds in each primary screening replicate, if available, is displayed in Table S3.

Table S1. Number of SD molecules after each filtering step (*Methods – Filtering steps*). 'Unf.' stands for unfiltered.

SD AID	# SD (unf.)	# SD step 1	# SD step 2	# SD step 3	# SD step 4	SD % filtered
1619	217,147	216,988	208,445	207,510	207,097	4.63
488975	306,595	306,502	294,182	292,685	292,251	4.68
2097	302,503	302,435	290,529	289,100	288,660	4.58
624330	342,291	342,059	328,593	325,396	324,979	5.06
504558	345,298	345,064	331,433	328,215	327,791	5.07
2221	293,466	293,376	281,810	280,435	280,006	4.59
1259416	69,082	67,275	66,823	59,553	59,447	13.95
1979	302,509	302,451	290,885	289,510	289,073	4.44
2732	219,164	218,654	209,533	208,542	208,123	5.04
2216	302,453	302,387	290,489	289,060	288,624	4.57
2553	305,679	305,610	293,627	292,189	291,749	4.56
651710	355,860	352,074	340,855	266,378	266,045	25.24
652162	352,852	349,124	337,953	265,305	264,972	24.91
1903	306,015	305,355	292,949	291,466	291,022	4.90
2099	328,736	326,066	313,544	311,519	311,075	5.37
489030	331,760	331,671	318,624	316,389	315,950	4.77
1662	303,545	303,477	291,564	290,134	289,697	4.56
743445	309,831	309,684	299,069	298,190	297,773	3.89
2227	305,669	305,600	293,617	292,179	291,739	4.56
435005	303,588	303,500	291,778	289,862	289,447	4.66
2098	301,406	301,324	289,497	288,068	287,633	4.57
2650	315,508	315,412	302,985	300,984	300,560	4.74
686996	347,992	344,318	333,429	261,697	261,370	24.89
873	214,261	214,187	205,699	204,774	204,361	4.62
652115	326,679	323,507	313,454	241,766	241,469	26.08
504582	336,846	336,519	323,169	320,733	320,309	4.91
1259416	69,082	67,275	66,823	59,553	59,447	13.95
1117319	262,345	258,750	248,950	247,605	247,230	5.76
1445	217,157	216,987	208,444	207,509	207,096	4.63
504621	307,324	307,132	294,564	292,392	292,004	4.98
504408	301,246	301,170	289,345	287,916	287,481	4.57
624304	364,167	363,840	349,806	346,019	345,553	5.11
652154	356,670	353,950	342,279	281,453	281,074	21.19
720511	347,956	343,786	333,297	252,700	252,387	27.47
602261	362,387	362,063	348,055	344,277	343,811	5.13
1224905	206,863	204,125	203,220	203,134	202,486	2.12
652115	326,679	323,507	313,454	241,766	241,469	26.08
488895	337,881	337,500	324,110	321,666	321,242	4.92
493091	340,929	340,696	327,127	324,616	324,171	4.92
2237	305,669	305,600	293,617	292,179	291,739	4.56
504329	335,445	335,224	321,919	319,496	319,080	4.88
2221	293,466	293,376	281,810	280,435	280,006	4.59
588489	359,520	359,244	345,412	341,943	341,503	5.01
485317	288,803	288,728	277,502	275,780	275,394	4.64
588549	355,325	355,054	341,361	337,922	337,483	5.02
2247	304,070	304,001	292,056	290,633	290,197	4.56
504558	345,298	345,064	331,433	328,215	327,791	5.07
651658	343,072	339,650	328,816	256,826	256,499	25.23
1832	301,856	301,788	289,931	288,510	288,074	4.57
2629	323,875	323,564	310,829	308,722	308,289	4.81
1832	301,856	301,788	289,931	288,510	288,074	4.57
485273	330,481	330,393	317,445	315,230	314,791	4.75
588689	338,853	338,623	325,300	322,116	321,708	5.06
449762	327,669	327,558	314,535	312,348	311,910	4.81
488899	331,578	331,360	318,101	315,719	315,297	4.91
2221	293,466	293,376	281,810	280,435	280,006	4.59
1465	215,402	215,289	206,686	205,603	205,193	4.74
1949	100,697	100,685	98,518	98,518	98,477	2.20
449739	104,742	104,728	100,946	100,333	100,197	4.34
1259374	646,073	642,362	618,414	614,888	614,427	4.90

Table S2. Number of DR molecules after each filtering step (*Methods – Filtering steps*). 'Unf.' stands for unfiltered.

SD AID	DR AID	DR (unf.)	# DR step 1	# DR step 2	# DR step 3	# DR step 4	DR % filtered	Filtered DR (not NaN)	DR % filtered (not NaN)
1619	-	827	827	813	812	811	1.93	811	1.93
488975	504840	1,544	1,544	1,422	1,416	1,416	8.29	805	47.86
2097	434954	2,198	2,198	2,106	2,103	2,101	4.41	576	73.79
624330	-	2,057	2,057	1,579	1,571	1,570	23.68	1,570	23.68
504558	588343	1,241	1,240	1,153	1,153	1,153	7.09	626	49.56
2221	449749	2,133	2,128	2,029	2,021	2,002	6.14	1,632	23.49
1259416	1259418	3,560	3,288	3,286	3,032	3,027	14.97	711	80.03
1979	2423	1,838	1,836	1,648	1,645	1,641	10.72	1,363	25.84
2732	504313	940	940	898	895	894	4.89	855	9.04
2216	435026	1,016	1,016	967	964	961	5.41	390	61.61
2553	2696	900	900	618	618	612	32.00	189	79.00
651710	652116	996	996	612	610	604	39.36	359	63.96
652162	720512	931	925	893	852	850	8.70	109	88.29
1903	-	1,203	1,203	1,136	1,132	1,129	6.15	1,129	6.15
2099	488835	1,413	1,413	1,342	1,337	1,335	5.52	140	90.09
489030	588524	476	474	422	421	409	14.08	408	14.29
1662	1914	3,266	3,264	3,116	3,114	3,105	4.93	2,374	27.31
743445	1053173	1,503	1,503	1,400	1,399	1,393	7.32	497	66.93
2227	434941	2,267	2,267	1,882	1,880	1,879	17.12	56	97.53
435005	449756	2,288	2,282	1,948	1,941	1,920	16.08	1,811	20.85
2098	2382	2,448	2,448	2,342	2,342	2,337	4.53	2,239	8.54
2650	463203	2,352	2,352	2,249	2,238	2,235	4.97	721	69.35
686996	720632	962	962	912	852	852	11.43	398	58.63
873	1431	1,260	1,260	1,217	1,217	1,215	3.57	1,215	3.57
652115	720591	1,194	1,194	1,138	1,136	1,135	4.94	169	85.85
504582	540271	826	826	789	788	788	4.60	474	42.62
1259416	1259420	1,220	1,021	1,019	717	712	41.64	174	85.74
1117319	1117362	3,634	3,629	3,506	3,501	3,493	3.88	77	97.88
1445	-	673	673	659	655	655	2.67	655	2.67
504621	540268	930	930	862	856	856	7.96	814	12.47
504408	435004	1,953	1,953	1,867	1,867	1,865	4.51	577	70.46
624304	624474	1,381	1,380	1,334	1,330	1,330	3.69	1,327	3.91
652154	687027	1,810	1,807	1,746	1,277	1,277	29.45	1,024	43.43
720511	743267	1,170	1,170	1,101	1,066	1,066	8.89	549	53.08
602261	624326	1,011	1,011	992	987	987	2.37	985	2.57
1224905	1259350	579	579	575	574	569	1.73	569	1.73
652115	720597	964	963	963	551	551	42.84	25	97.41
488895	504941	1,215	1,215	1,135	1,134	1,134	6.67	161	86.75
493091	540297	1,011	1,011	927	926	922	8.80	919	9.10
2237	434937	2,267	2,267	1,882	1,880	1,879	17.12	573	74.72
504329	-	1,010	1,010	908	903	902	10.69	902	10.69
2221	449750	2,133	2,128	2,029	2,021	2,002	6.14	1,405	34.13
588489	602259	1,186	1,186	1,139	1,139	1,129	4.81	1,128	4.89
485317	493248	2,345	2,345	2,207	2,207	2,202	6.10	1,367	41.71
588549	624273	1,047	1,047	1,020	1,019	1,016	2.96	359	65.71
2247	434942	2,267	2,267	1,882	1,880	1,879	17.12	584	74.24
504558	588398	1,241	1,240	1,153	1,153	1,153	7.09	102	91.78
651658	687022	1,025	1,025	1,005	922	922	10.05	344	66.44
1832	1960	1,691	1,690	1,630	1,625	1,615	4.49	1,588	6.09
2629	435023	1,430	1,430	1,381	1,381	1,378	3.64	87	93.92
1832	1964	1,691	1,690	1,630	1,625	1,615	4.49	1,604	5.14
485273	493155	1,210	1,210	989	980	978	19.17	973	19.59
588689	-	1,013	1,013	987	975	973	3.95	973	3.95
449762	-	1,938	1,938	1,763	1,758	1,754	9.49	1,754	9.49
488899	493073	1,241	1,241	1,133	1,129	1,124	9.43	704	43.27
2221	435010	2,133	2,128	2,029	2,021	2,002	6.14	1,797	15.75
1465	-	159	159	152	147	147	7.55	147	7.55
1949	-	1,782	1,782	1,694	1,694	1,693	4.99	1,688	5.27
449739	489005	895	895	860	860	860	3.91	859	4.02
1259374	1259375	474	470	372	358	358	24.47	348	26.58

Table S3. Number of compounds in each of the primary screening replicates for each SD dataset. Dataset with low number of replicates have empty values.

Dataset	# replicates 1	# replicates 2	# replicates 3	# replicates 4	# replicates 5
AID1619	217,147	61			
AID488975	306,595				
AID2097	302,503	12,803			
AID624330	342,291	3,835	2,560		
AID504558	345,298	340,565	320	320	
AID2221	293,466	2,002	1,999	1,851	
AID1259416	69,082	68,762			
AID1979	302,509	187,149	23,915	8,579	
AID2732	219,164				
AID2216	302,453	302,133	11,203	8,323	
AID2553	305,679				
AID651710	355,860				
AID652162	352,852				
AID1903	306,015	44,705	6,511	48	
AID2099	328,736	3,366	4		
AID489030	331,760				
AID1662	285,555	283,634	41,921	40,321	
AID743445	309,831				
AID2227	305,669				
AID435005	303,588	302,169	3,144	2,884	
AID2098	301,406	301,086	7,043	7,043	
AID1259374	646,073				
AID2650	315,508	644			
AID686996	347,992				
AID873	214,261				
AID1949	100,691	3,200	3,189		
AID652115	326,679				
AID504582	336,846	336,205			
AID1259416	69,082	68,762			
AID1117319	262,344	45,176	12,784	12,783	
AID1445	217,146	10,300	10,239		
AID504621	307,324	306,594			
AID504408	301,246	300,926	7,039	7,039	
AID624304	364,167				
AID652154	356,670	354,112			
AID720511	347,956	148,907			
AID602261	362,387				
AID1224905	206,505	205,449			
AID652115	326,679				
AID488895	337,881	322,743			
AID493091	340,929				
AID449739	104,742				
AID2237	305,669				
AID504329	335,445	11,011	11,011		
AID2221	293,466	2,002	1,999	1,851	
AID588489	359,520				
AID485317	288,803	268,709			
AID588549	355,325	350,623			
AID2247	304,070				
AID504558	345,298	340,565	320	320	
AID651658	343,072				
AID1832	301,856	298,958	1,283	1,283	
AID2629	323,875	295,475	1,901	1,311	
AID1832	301,856	298,958	1,283	1,283	
AID485273	330,481				
AID588689	338,853	10,240	10,240		
AID449762	327,669	6,133			
AID488899	331,578	329,574			
AID2221	293,466	2,002	1,999	1,851	
AID1465	215,398	11,943	9,445	1,602	12

SI 2 Distributions of primary screening replicates

We preferred kernel density estimation over traditional histograms to increase readability. Only primary screening datasets with more than 2 replicates are illustrated.

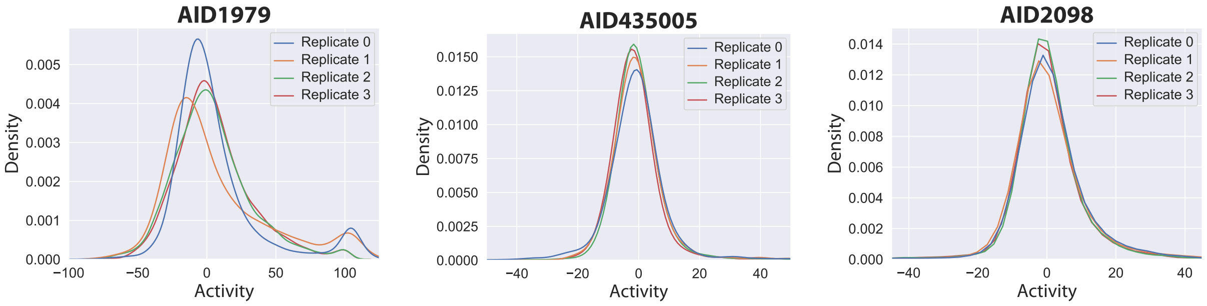

Figure S1. Kernel density estimation plots for AID1979, AID435005, AID2098.

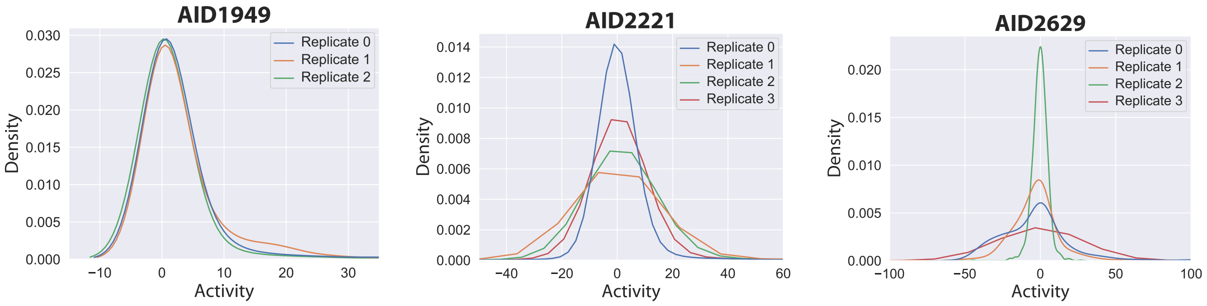

Figure S2. Kernel density estimation plots for AID1949, AID2221, AID2629.

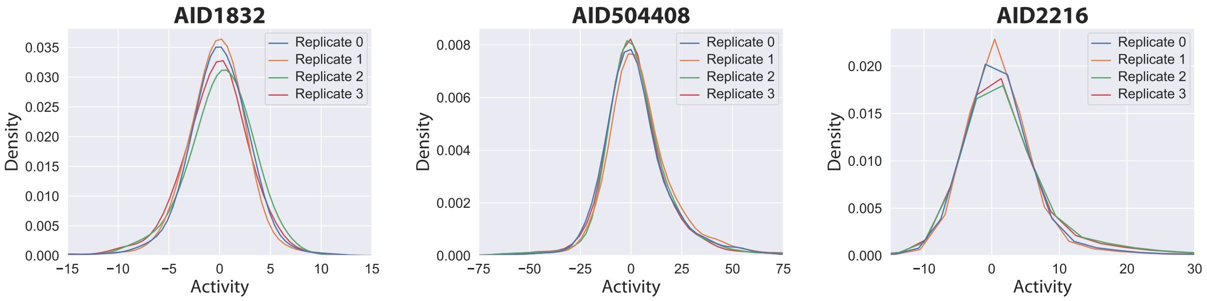

Figure S3. Kernel density estimation plots for AID1832, AID504408, AID2216.

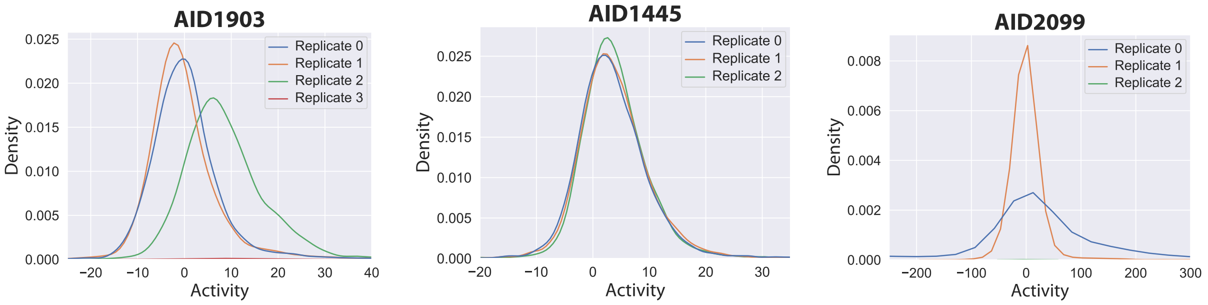

Figure S4. Kernel density estimation plots for AID1903, AID1445, AID2099.

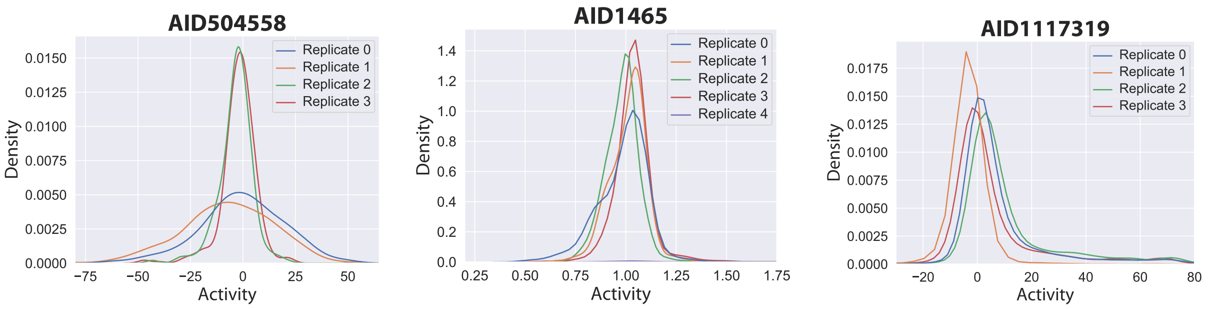

Figure S5. Kernel density estimation plots for AID504558, AID1465, AID1117319.

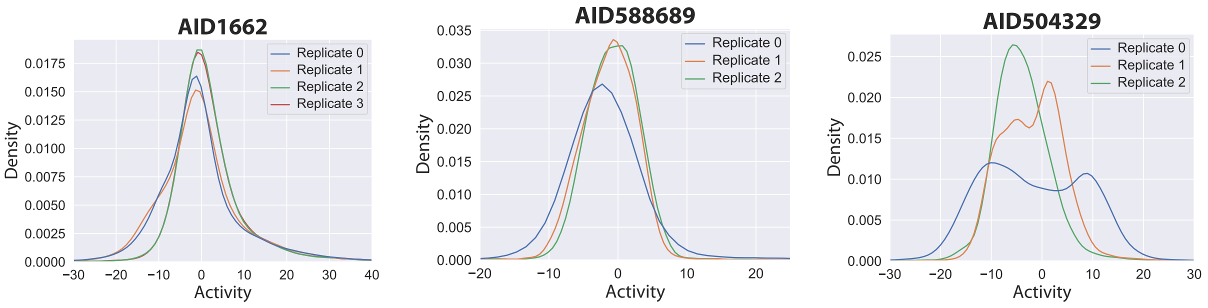

Figure S6. Kernel density estimation plots for AID1662, AID588689, AID504329.

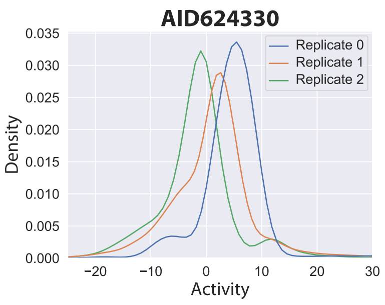

Figure S7. Kernel density estimation plots for AID624330.

Table S4. Table containing the primary screening datasets with more than 2 replicates and the number of compounds having measurements in all replicates, alongside the mean absolute error computed between the mean and median for the replicates. For datasets where the last replicate had less than 50 measurements, this last replicate column was discarded from the calculations. For example, AID1903 uses its first 3 replicates and AID2099 is dropped completely due to having only 4 measurements for the third replicate, resulting in equal mean and median. With the exception of a few datasets (e.g. AID1979, AID2629), the difference between the two is within the margin of error for the respective datasets.

Dataset	# replicates	Mean – Median MAE
AID624330	2,559	1.56
AID504558	320	2.69
AID2221	1,851	1.35
AID1979	8,577	5.21
AID2216	8,323	1.57
AID1903	6,510	2.34
AID1662	24,240	0.84
AID435005	2,884	0.90
AID2098	7,043	0.80
AID1949	3,189	1.83
AID1117319	4,630	2.09
AID1445	10,239	0.90
AID504408	7,039	1.06
AID504329	11,007	1.64
AID2221	1,851	1.35
AID504558	320	2.69
AID1832	1,283	0.48
AID2629	1,311	4.05
AID1832	1,283	0.48
AID588689	10,236	1.14
AID2221	1,851	1.35
AID1465	1,602	0.02

MAE, RMSE, Maximum error, and R^2 plots across MF-PCBA-37 datasets

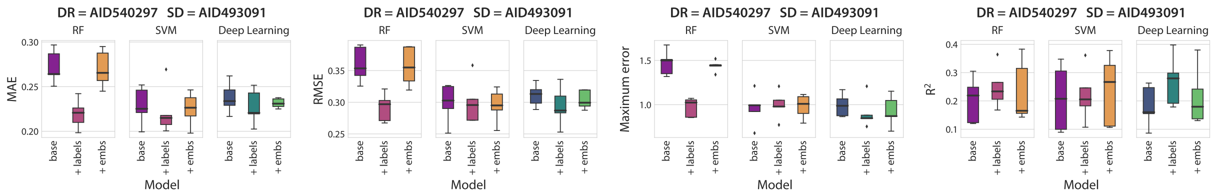

Figure S8. Test MAE, RMSE, maximum error, and R^2 for AID540297 – AID493091.

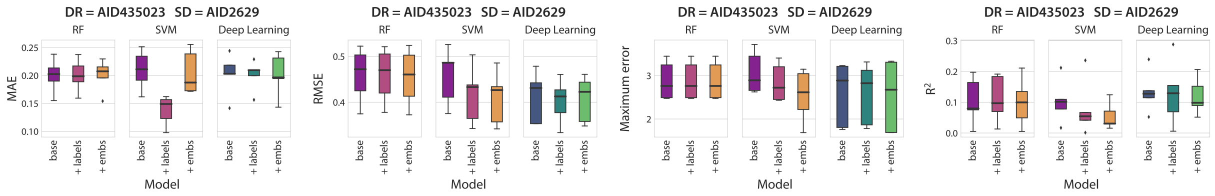

Figure S9. Test MAE, RMSE, maximum error, and R^2 for AID435023 – AID2629.

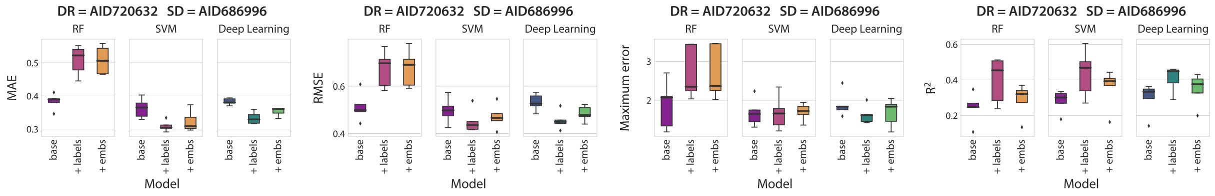

Figure S10. Test MAE, RMSE, maximum error, and R^2 for AID720632 – AID686996.

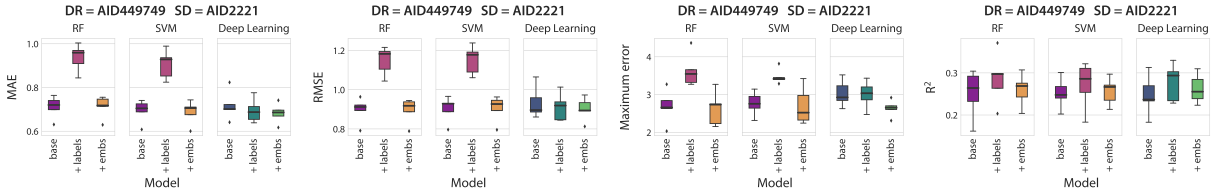

Figure S11. Test MAE, RMSE, maximum error, and R^2 for AID449749 – AID2221.

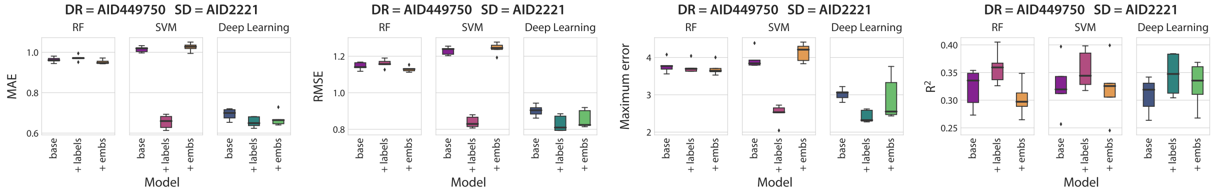

Figure S12. Test MAE, RMSE, maximum error, and R^2 for AID449750 – AID2221.

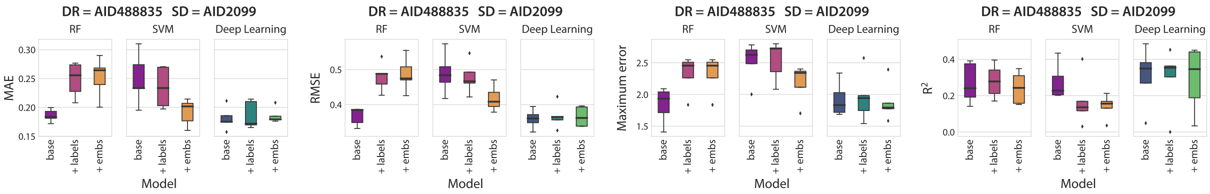

Figure S13. Test MAE, RMSE, maximum error, and R^2 for AID488835 – AID2099.

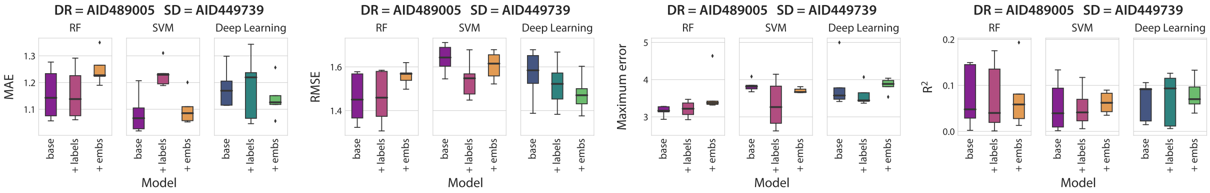

Figure S14. Test MAE, RMSE, maximum error, and R^2 for AID489005 – AID449739.

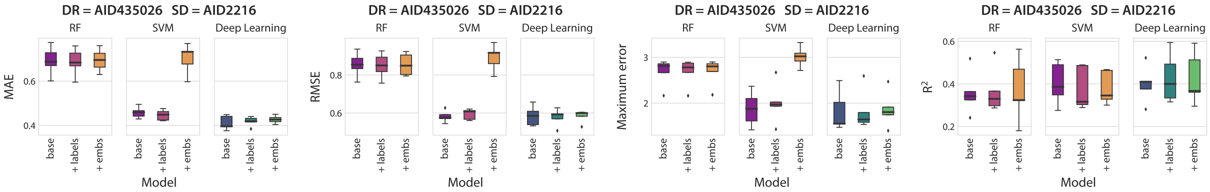

Figure S15. Test MAE, RMSE, maximum error, and R^2 for AID435026 – AID2216.

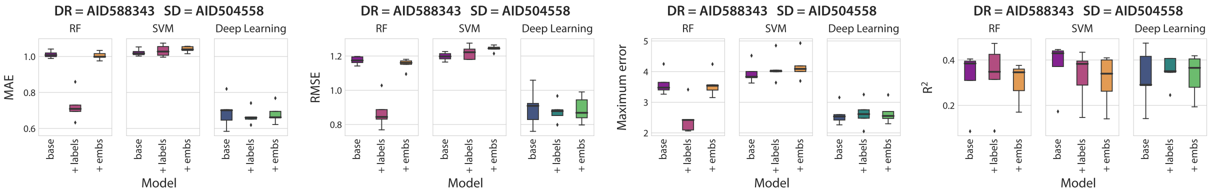

Figure S16. Test MAE, RMSE, maximum error, and R^2 for AID588343 – AID504558.

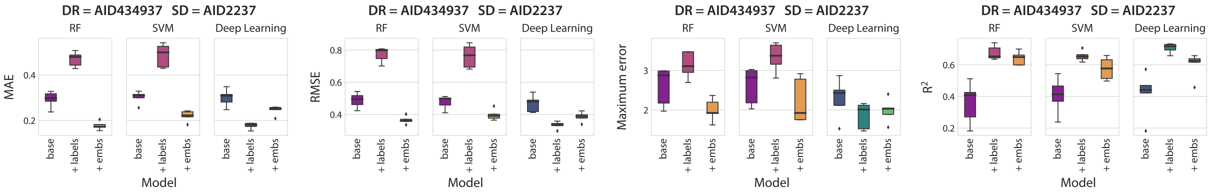

Figure S17. Test MAE, RMSE, maximum error, and R^2 for AID434937 – AID2237.

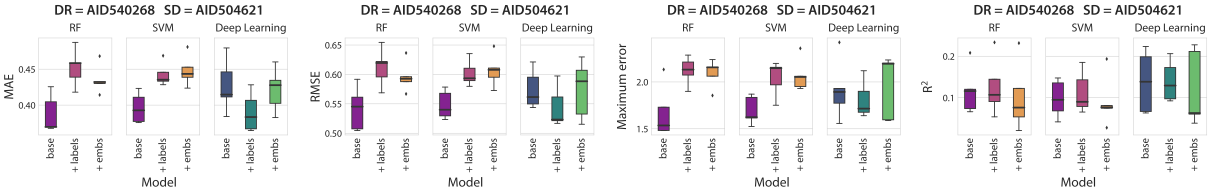

Figure S18. Test MAE, RMSE, maximum error, and R^2 for AID540268 – AID504621.

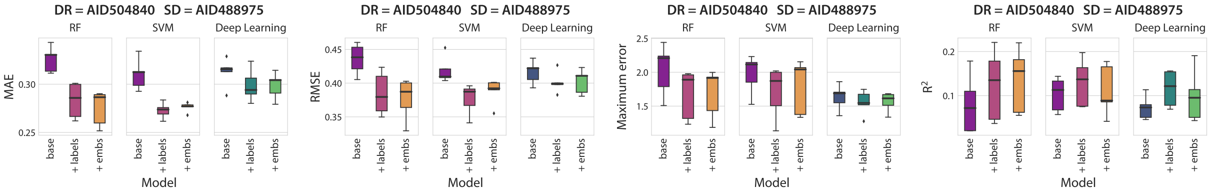

Figure S19. Test MAE, RMSE, maximum error, and R^2 for AID504840 – AID488975.

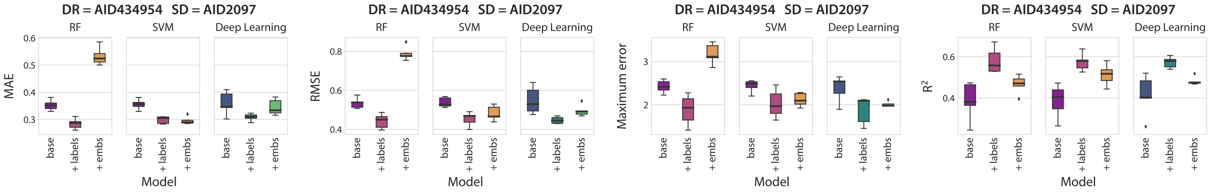

Figure S20. Test MAE, RMSE, maximum error, and R^2 for AID434954 – AID2097.

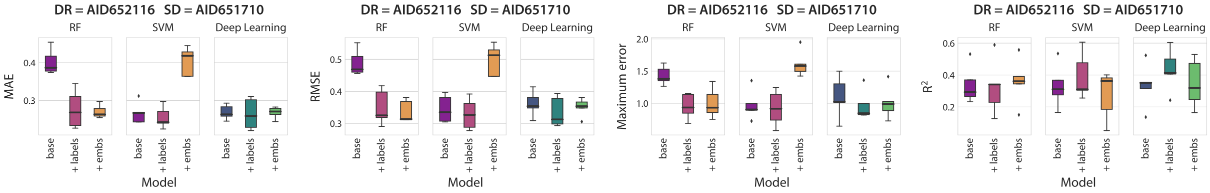

Figure S21. Test MAE, RMSE, maximum error, and R^2 for AID652116 – AID651710.

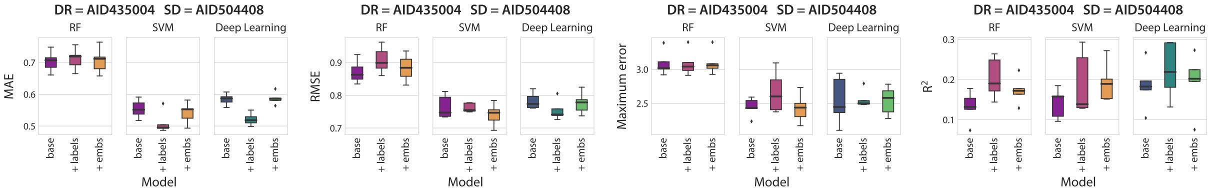

Figure S22. Test MAE, RMSE, maximum error, and R^2 for AID435004 – AID504408.

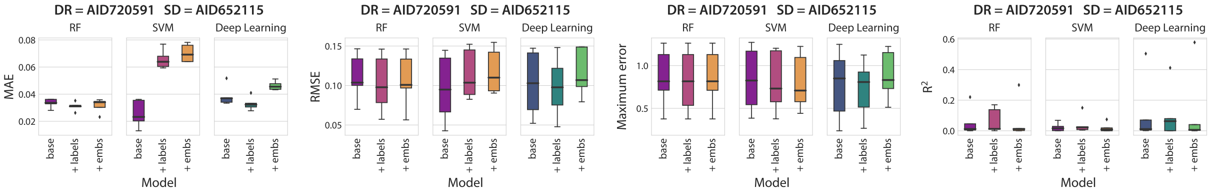

Figure S23. Test MAE, RMSE, maximum error, and R^2 for AID720591 – AID652115.

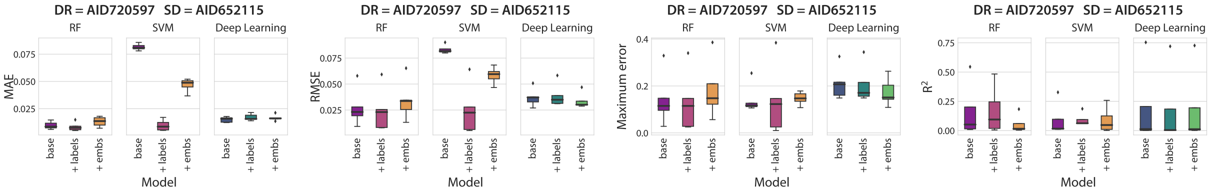

Figure S24. Test MAE, RMSE, maximum error, and R^2 for AID720597 – AID652115.

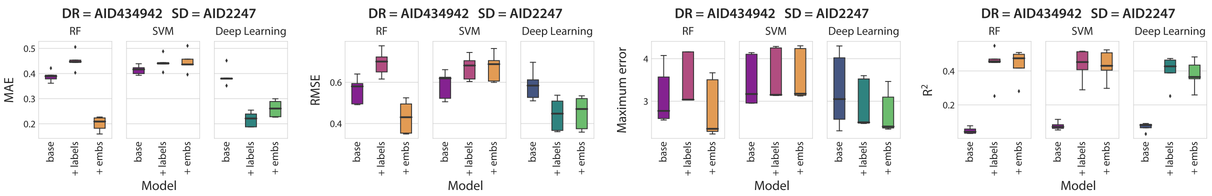

Figure S25. Test MAE, RMSE, maximum error, and R^2 for AID434942 – AID2247.

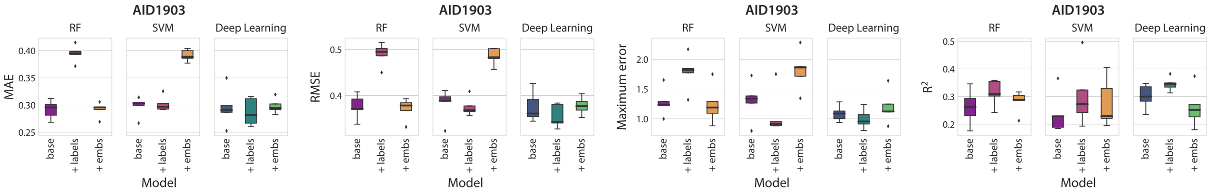

Figure S26. Test MAE, RMSE, maximum error, and R^2 for AID1903.

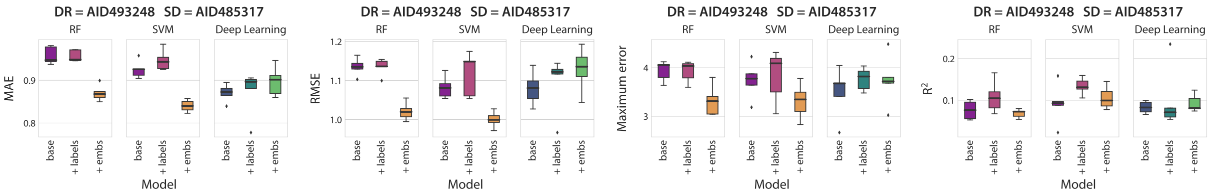

Figure S27. Test MAE, RMSE, maximum error, and R^2 for AID493248 – AID485317.

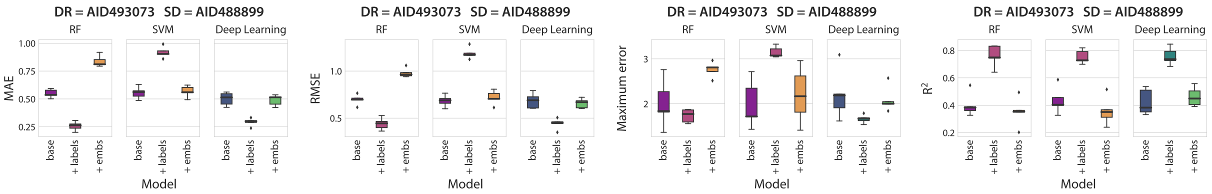

Figure S28. Test MAE, RMSE, maximum error, and R^2 for AID493073 – AID488899.

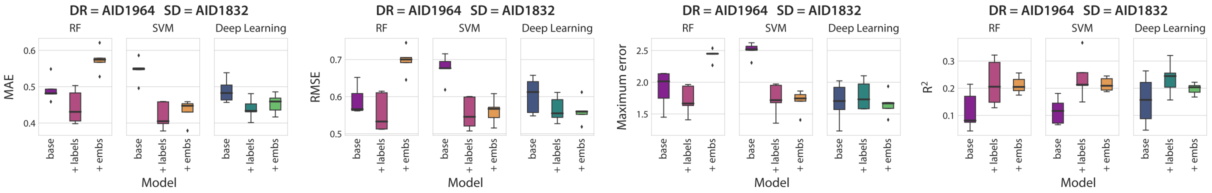

Figure S29. Test MAE, RMSE, maximum error, and R^2 for AID1964 – AID1832.

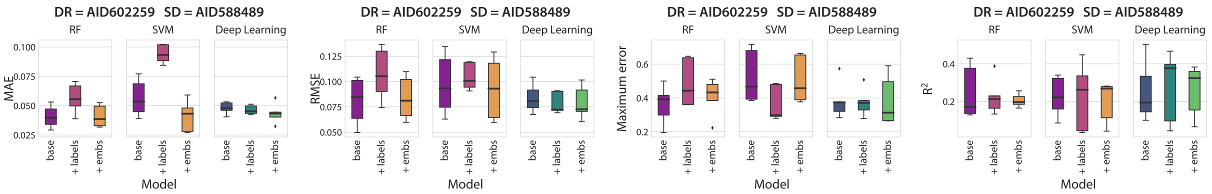

Figure S30. Test MAE, RMSE, maximum error, and R^2 for AID602259 – AID588489.

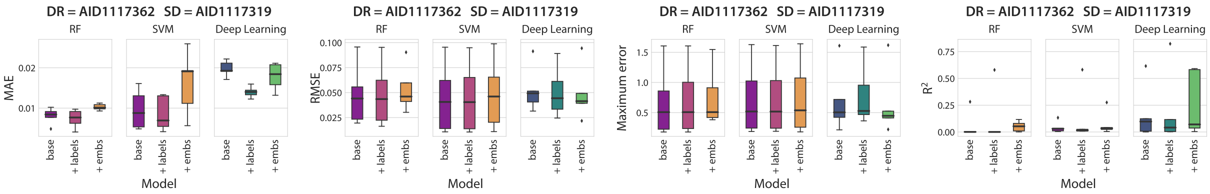

Figure S31. Test MAE, RMSE, maximum error, and R^2 for AID1117362 – AID1117319.

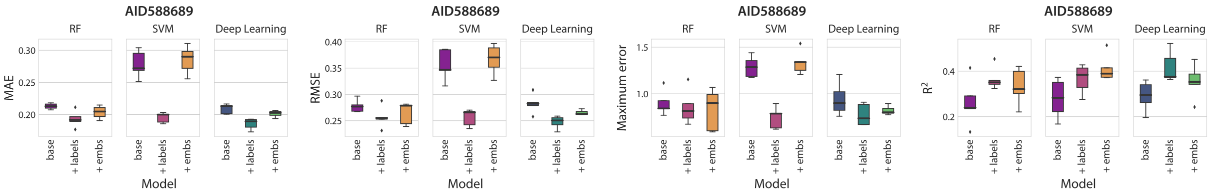

Figure S32. Test MAE, RMSE, maximum error, and R^2 for AID588689.

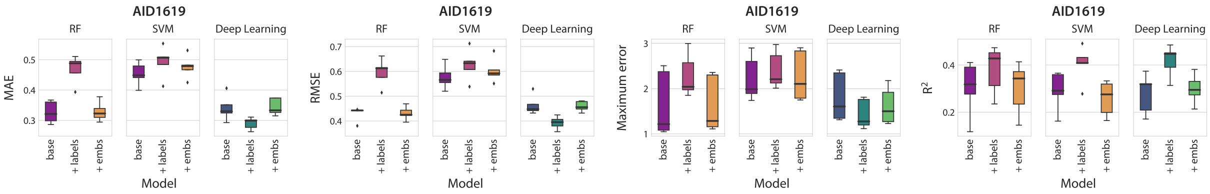

Figure S33. Test MAE, RMSE, maximum error, and R^2 for AID1619.

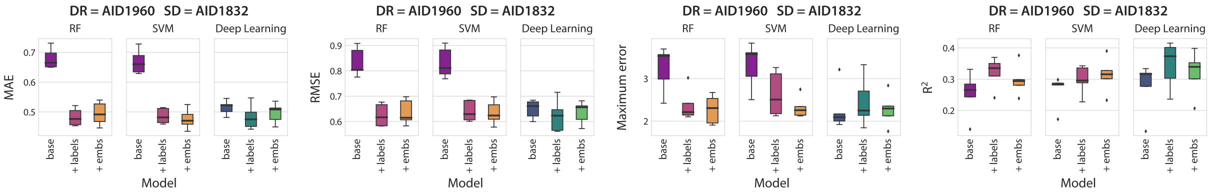

Figure S34. Test MAE, RMSE, maximum error, and R^2 for AID1960 – AID1832.

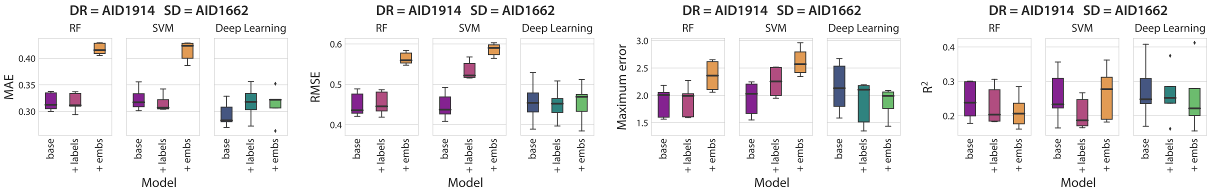

Figure S35. Test MAE, RMSE, maximum error, and R^2 for AID1914 – AID1662.

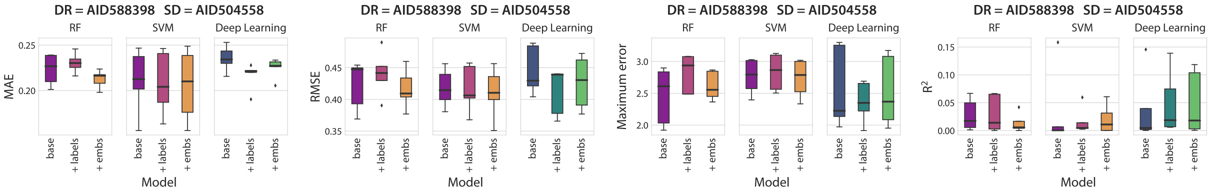

Figure S36. Test MAE, RMSE, maximum error, and R^2 for AID588398 – AID504558.

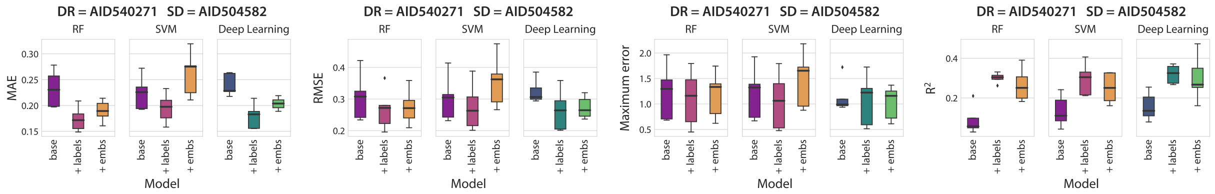

Figure S37. Test MAE, RMSE, maximum error, and R^2 for AID540271 – AID504582.

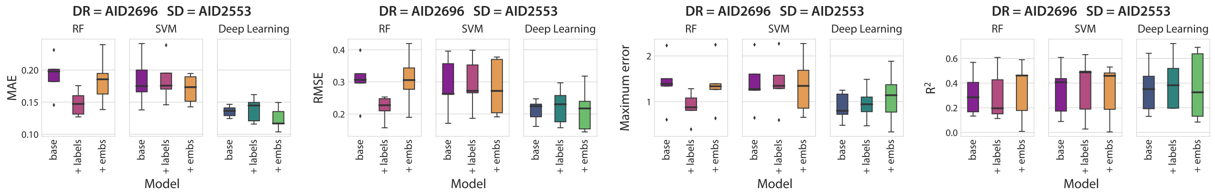

Figure S38. Test MAE, RMSE, maximum error, and R^2 for AID2696 – AID2553.

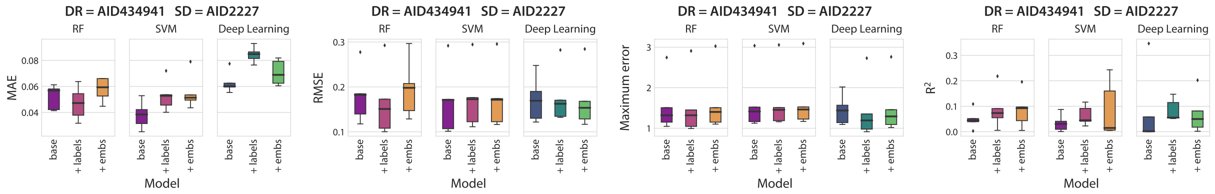

Figure S39. Test MAE, RMSE, maximum error, and R^2 for AID434941 – AID2227.

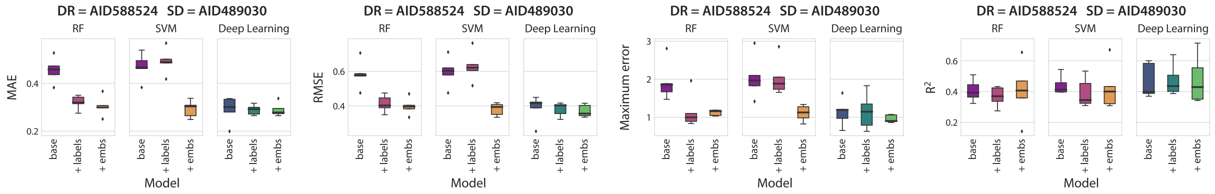

Figure S40. Test MAE, RMSE, maximum error, and R^2 for AID588524 – AID489030.

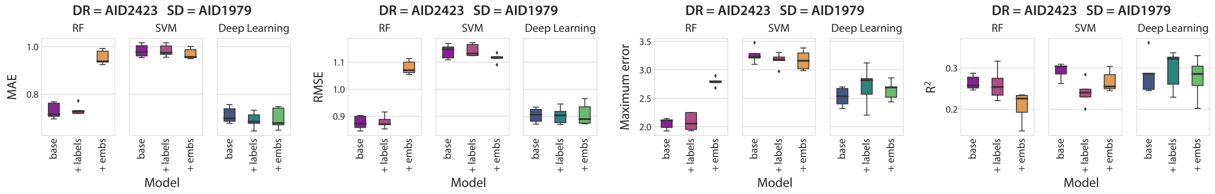

Figure S41. Test MAE, RMSE, maximum error, and R^2 for AID2423 – AID1979.

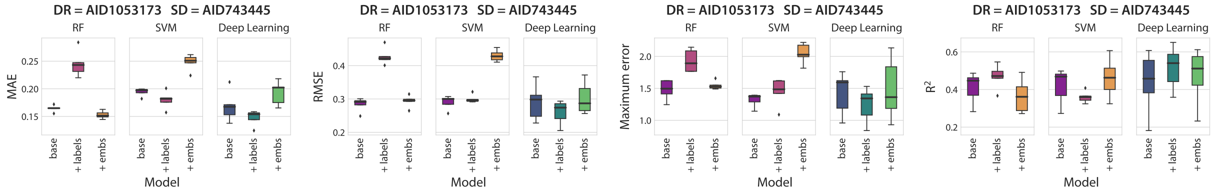

Figure S42. Test MAE, RMSE, maximum error, and R^2 for AID1053173 – AID743445.

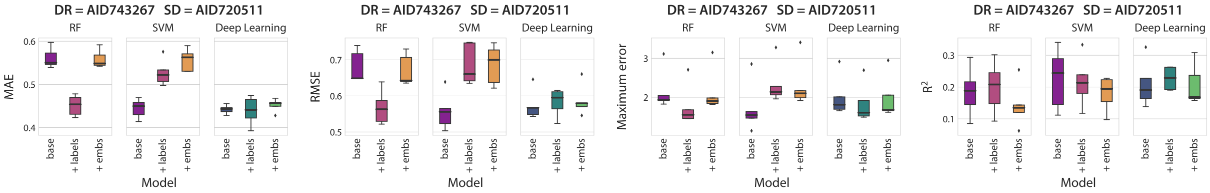

Figure S43. Test MAE, RMSE, maximum error, and R^2 for AID743267 – AID720511.

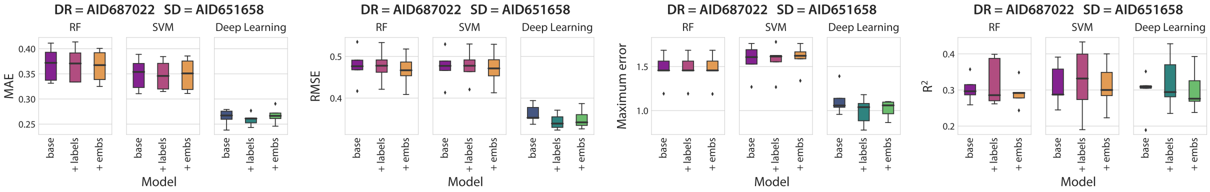

Figure S44. Test MAE, RMSE, maximum error, and R^2 for AID687022 – AID651658.

SI 3 $1 - \mathbf{R}^2$ ratio between augmented and baseline models

This alternative metric is listed in Tables S5 and S6 and is computed according to the formula $\frac{(1-\mathrm{R}^2_{\mathrm{aug}})}{(1-\mathrm{R}^2_{\mathrm{base}})}$, where 'aug' refers to an augmented model and 'base' to a non-augmented model.

Table S5. The ratio between '+ labels' augmented and baseline models is computed according to the formula $\frac{(1-R^2_{aug})}{(1-R^2_{base})}$, for all three categories of algorithms (deep learning, RF, SVM) on the full MF-PCBA collection.

Dataset	Deep Learning	RF	SVM
AID1053173-743445	0.87 ± 0.06	0.90 ± 0.06	1.12 ± 0.15
AID1117362-1117319	1.16 ± 0.84	0.92 ± 0.19	0.90 ± 0.23
AID1259350-1224905	0.91 ± 0.09	0.90 ± 0.17	0.85 ± 0.14
AID1259375-1259374	0.95 ± 0.12	1.06 ± 0.21	1.12 ± 0.20
AID1259418-1259416	0.94 ± 0.08	0.85 ± 0.07	0.91 ± 0.13
AID1259420-1259416	0.82 ± 0.26	1.05 ± 0.17	0.99 ± 0.29
AID1431-873	0.99 ± 0.09	0.97 ± 0.09	1.14 ± 0.29
AID1445	0.36 ± 0.08	0.37 ± 0.10	0.40 ± 0.15
AID1465	1.00 ± 0.05	1.01 ± 0.03	0.99 ± 0.00
AID1619	0.81 ± 0.05	0.89 ± 0.05	0.84 ± 0.06
AID1903	0.94 ± 0.06	0.93 ± 0.05	0.92 ± 0.21
AID1914-1662	1.02 ± 0.03	1.02 ± 0.03	1.07 ± 0.06
AID1949	1.00 ± 0.04	0.99 ± 0.06	1.00 ± 0.05
AID1960-1832	0.91 ± 0.09	0.91 ± 0.05	0.96 ± 0.04
AID1964-1832	0.91 ± 0.08	0.89 ± 0.13	0.86 ± 0.11
AID2382-2098	0.94 ± 0.08	0.99 ± 0.11	0.96 ± 0.04
AID2423-1979	0.99 ± 0.06	1.01 ± 0.06	1.08 ± 0.06
AID2696-2553	0.91 ± 0.08	1.01 ± 0.11	0.95 ± 0.08
AID434937-2237	0.52 ± 0.08	0.51 ± 0.06	0.59 ± 0.08
AID434941-2227	1.03 ± 0.24	0.96 ± 0.11	0.97 ± 0.07
AID434942-2247	0.64 ± 0.10	0.59 ± 0.13	0.61 ± 0.12
AID434954-2097	0.75 ± 0.14	0.70 ± 0.18	0.71 ± 0.16
AID435004-504408	0.95 ± 0.06	0.92 ± 0.06	0.95 ± 0.09
AID435010-2221	0.80 ± 0.11	0.77 ± 0.12	0.78 ± 0.11
AID435023-2629	1.00 ± 0.06	0.99 ± 0.01	1.03 ± 0.04
AID435026-2216	0.95 ± 0.11	0.99 ± 0.05	1.04 ± 0.06
AID449749-2221	0.96 ± 0.06	0.95 ± 0.03	0.97 ± 0.07
AID449750-2221	0.95 ± 0.05	0.95 ± 0.05	0.96 ± 0.05
AID449756-435005	0.93 ± 0.13	0.92 ± 0.12	0.97 ± 0.15
AID449762	0.96 ± 0.08	0.90 ± 0.06	0.92 ± 0.04
AID463203-2650	0.85 ± 0.21	0.86 ± 0.14	0.88 ± 0.15
AID488835-2099	1.02 ± 0.05	0.99 ± 0.06	1.14 ± 0.07
AID489005-449739	0.99 ± 0.02	1.00 ± 0.02	1.02 ± 0.06
AID493073-488899	0.42 ± 0.10	0.42 ± 0.21	0.45 ± 0.16
AID493155-485273	0.68 ± 0.06	0.72 ± 0.21	0.69 ± 0.12
AID493248-485317	0.98 ± 0.08	0.97 ± 0.04	0.96 ± 0.04
AID504313-2732	0.98 ± 0.05	1.01 ± 0.01	1.03 ± 0.03
AID504329	0.63 ± 0.14	0.52 ± 0.14	0.55 ± 0.13
AID504840-488975	0.95 ± 0.04	0.95 ± 0.05	0.97 ± 0.03
AID504941-488895	0.69 ± 0.22	0.71 ± 0.21	0.69 ± 0.24
AID540268-504621	1.00 ± 0.06	0.99 ± 0.02	0.98 ± 0.06
AID540271-504582	0.81 ± 0.11	0.77 ± 0.06	0.82 ± 0.13
AID540297-493091	0.90 ± 0.11	0.95 ± 0.05	1.00 ± 0.13
AID588343-504558	0.97 ± 0.10	0.98 ± 0.08	1.07 ± 0.05
AID588398-504558	0.99 ± 0.07	1.00 ± 0.01	1.02 ± 0.09
AID588524-489030	1.02 ± 0.28	1.08 ± 0.15	1.08 ± 0.06
AID588689	0.82 ± 0.10	0.88 ± 0.18	0.88 ± 0.09
AID602259-588489	0.97 ± 0.14	1.06 ± 0.23	0.99 ± 0.12
AID624273-588549	0.78 ± 0.18	0.77 ± 0.16	0.75 ± 0.07
AID624326-602261	0.65 ± 0.10	0.62 ± 0.11	0.66 ± 0.11
AID624330	0.69 ± 0.12	0.74 ± 0.06	0.71 ± 0.04
AID624474-624304	0.69 ± 0.13	0.74 ± 0.21	0.84 ± 0.36
AID652116-651710	0.85 ± 0.05	1.01 ± 0.10	0.91 ± 0.13
AID687022-651658	0.96 ± 0.09	0.97 ± 0.06	0.98 ± 0.06
AID687027-652154	0.94 ± 0.13	0.96 ± 0.13	1.07 ± 0.13
AID720512-652162	0.88 ± 0.56	0.57 ± 0.44	0.58 ± 0.43
AID720591-652115	1.10 ± 0.48	0.99 ± 0.08	0.98 ± 0.04
AID720597-652115	1.04 ± 0.07	1.01 ± 0.09	1.03 ± 0.25
AID720632-686996	0.84 ± 0.03	0.79 ± 0.15	0.78 ± 0.14
AID743267-720511	0.98 ± 0.07	0.98 ± 0.01	1.02 ± 0.07

Table S6. The ratio between '+ embs' augmented and baseline models is computed according to the formula $\frac{(1-R^2_{aug})}{(1-R^2_{base})}$, for all three categories of algorithms (deep learning, RF, SVM) on the full MF-PCBA collection.

Dataset	Deep Learning	RF	SVM
AID1053173-743445	0.95 ± 0.11	1.08 ± 0.10	0.92 ± 0.08
AID1117362-1117319	0.92 ± 0.28	1.02 ± 0.17	0.96 ± 0.07
AID1259350-1224905	0.98 ± 0.03	0.93 ± 0.14	0.91 ± 0.12
AID1259375-1259374	0.97 ± 0.10	1.22 ± 0.44	1.06 ± 0.08
AID1259418-1259416	1.05 ± 0.10	1.01 ± 0.04	1.03 ± 0.03
AID1259420-1259416	0.98 ± 0.26	1.10 ± 0.22	0.95 ± 0.09
AID1431-873	1.04 ± 0.14	1.11 ± 0.10	1.06 ± 0.11
AID1445	0.54 ± 0.05	0.63 ± 0.16	0.53 ± 0.12
AID1465	0.98 ± 0.02	1.02 ± 0.05	0.98 ± 0.02
AID1619	0.98 ± 0.06	1.01 ± 0.09	1.05 ± 0.04
AID1903	1.06 ± 0.10	0.98 ± 0.07	0.95 ± 0.05
AID1914-1662	1.03 ± 0.03	1.04 ± 0.03	0.99 ± 0.04
AID1949	1.01 ± 0.08	1.01 ± 0.04	1.04 ± 0.05
AID1960-1832	0.94 ± 0.07	0.94 ± 0.09	0.93 ± 0.05
AID1964-1832	0.96 ± 0.09	0.90 ± 0.08	0.89 ± 0.04
AID2382-2098	0.99 ± 0.04	0.99 ± 0.03	0.92 ± 0.05
AID2423-1979	1.01 ± 0.04	1.08 ± 0.05	1.04 ± 0.03
AID2696-2553	0.94 ± 0.17	0.97 ± 0.26	1.03 ± 0.22
AID434937-2237	0.69 ± 0.06	0.57 ± 0.05	0.72 ± 0.11
AID434941-2227	1.04 ± 0.24	0.96 ± 0.08	0.95 ± 0.13
AID434942-2247	0.67 ± 0.09	0.59 ± 0.11	0.62 ± 0.11
AID434954-2097	0.90 ± 0.12	0.89 ± 0.19	0.81 ± 0.18
AID435004-504408	0.99 ± 0.03	0.95 ± 0.04	0.94 ± 0.05
AID435010-2221	1.00 ± 0.05	1.03 ± 0.02	1.04 ± 0.03
AID435023-2629	1.02 ± 0.08	1.00 ± 0.03	1.06 ± 0.05
AID435026-2216	0.96 ± 0.15	0.98 ± 0.16	1.04 ± 0.05
AID449749-2221	0.98 ± 0.04	0.99 ± 0.05	0.99 ± 0.02
AID449750-2221	0.97 ± 0.06	1.03 ± 0.05	1.01 ± 0.09
AID449756-435005	1.00 ± 0.12	1.04 ± 0.08	1.00 ± 0.03
AID449762	1.02 ± 0.05	0.99 ± 0.03	1.00 ± 0.03
AID463203-2650	0.89 ± 0.09	0.90 ± 0.13	0.84 ± 0.06
AID488835-2099	1.02 ± 0.10	1.04 ± 0.05	1.20 ± 0.13
AID489005-449739	0.99 ± 0.06	1.00 ± 0.05	0.99 ± 0.05
AID493073-488899	0.94 ± 0.06	1.08 ± 0.07	1.14 ± 0.04
AID493155-485273	0.92 ± 0.07	1.04 ± 0.22	0.96 ± 0.12
AID493248-485317	0.99 ± 0.01	1.01 ± 0.02	0.99 ± 0.04
AID504313-2732	1.00 ± 0.05	1.02 ± 0.02	1.04 ± 0.03
AID504329	0.81 ± 0.08	0.82 ± 0.27	0.74 ± 0.04
AID504840-488975	0.97 ± 0.06	0.94 ± 0.07	0.99 ± 0.07
AID504941-488895	0.91 ± 0.15	0.85 ± 0.14	0.92 ± 0.18
AID540268-504621	1.02 ± 0.04	1.02 ± 0.05	1.01 ± 0.04
AID540271-504582	0.83 ± 0.14	0.81 ± 0.13	0.87 ± 0.13
AID540297-493091	0.96 ± 0.07	0.96 ± 0.07	0.96 ± 0.05
AID588343-504558	0.99 ± 0.08	1.03 ± 0.07	1.10 ± 0.07
AID588398-504558	0.99 ± 0.04	1.02 ± 0.02	1.03 ± 0.06
AID588524-489030	1.01 ± 0.39	0.98 ± 0.21	1.01 ± 0.18
AID588689	0.91 ± 0.06	0.92 ± 0.14	0.82 ± 0.06
AID602259-588489	1.02 ± 0.21	1.09 ± 0.25	1.04 ± 0.06
AID624273-588549	0.84 ± 0.21	0.78 ± 0.16	0.76 ± 0.15
AID624326-602261	0.90 ± 0.08	0.99 ± 0.10	0.92 ± 0.04
AID624330	0.84 ± 0.09	0.84 ± 0.05	0.77 ± 0.07
AID624474-624304	0.85 ± 0.08	0.81 ± 0.12	0.78 ± 0.16
AID652116-651710	0.98 ± 0.11	0.96 ± 0.11	1.10 ± 0.22
AID687022-651658	0.99 ± 0.08	1.02 ± 0.02	1.00 ± 0.02
AID687027-652154	1.03 ± 0.05	1.02 ± 0.08	0.98 ± 0.10
AID720512-652162	0.96 ± 0.54	0.73 ± 0.32	1.04 ± 0.37
AID720591-652115	0.98 ± 0.07	0.99 ± 0.05	1.00 ± 0.01
AID720597-652115	1.03 ± 0.06	1.27 ± 0.60	1.01 ± 0.07
AID720632-686996	0.93 ± 0.04	0.94 ± 0.03	0.89 ± 0.08
AID743267-720511	1.00 ± 0.02	1.05 ± 0.03	1.07 ± 0.07

SI 4 ΔR^2 versus SD/DR correlation for the embeddings augmentation

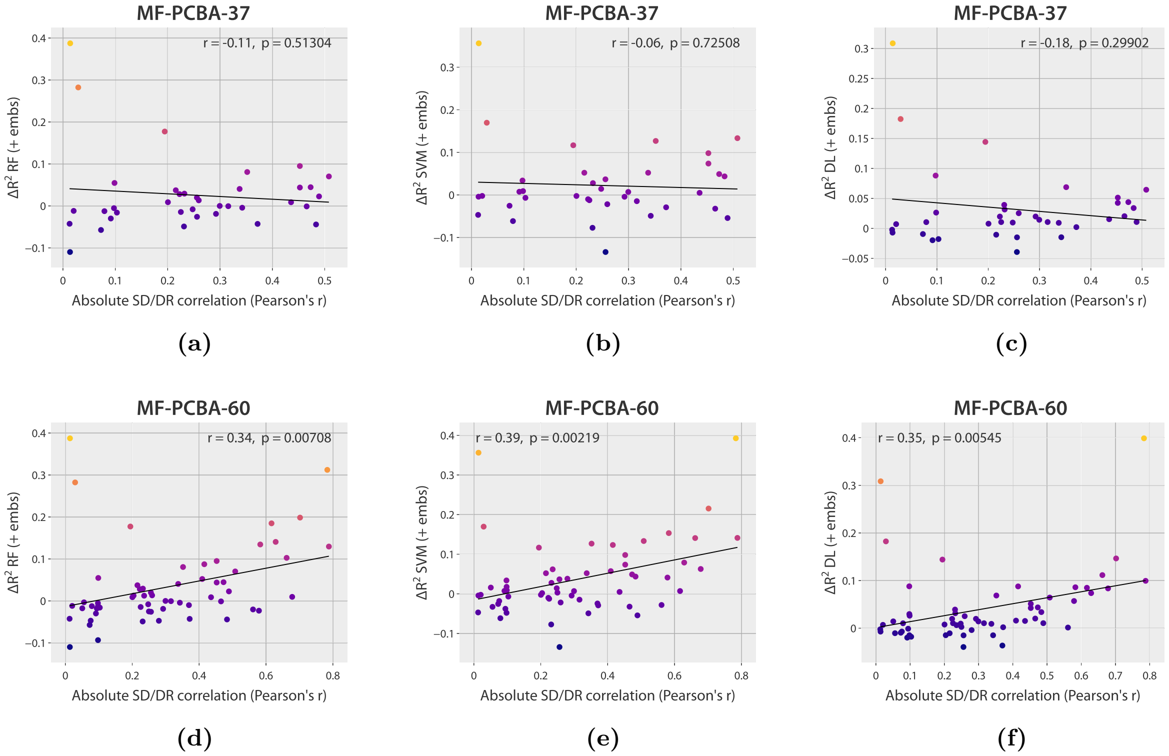

Figure S45. Plots of the Absolute SD/DR correlation (x-axis) against the ΔR^2 (y-axis) for RF, SVM, and deep learning models augmented with SD embeddings on MF-PCBA-37 and MF-PCBA-60, with the Pearson correlation coefficient (r). Brighter colours correspond to higher ΔR^2 values.

SI 5 R^2 versus ROGI

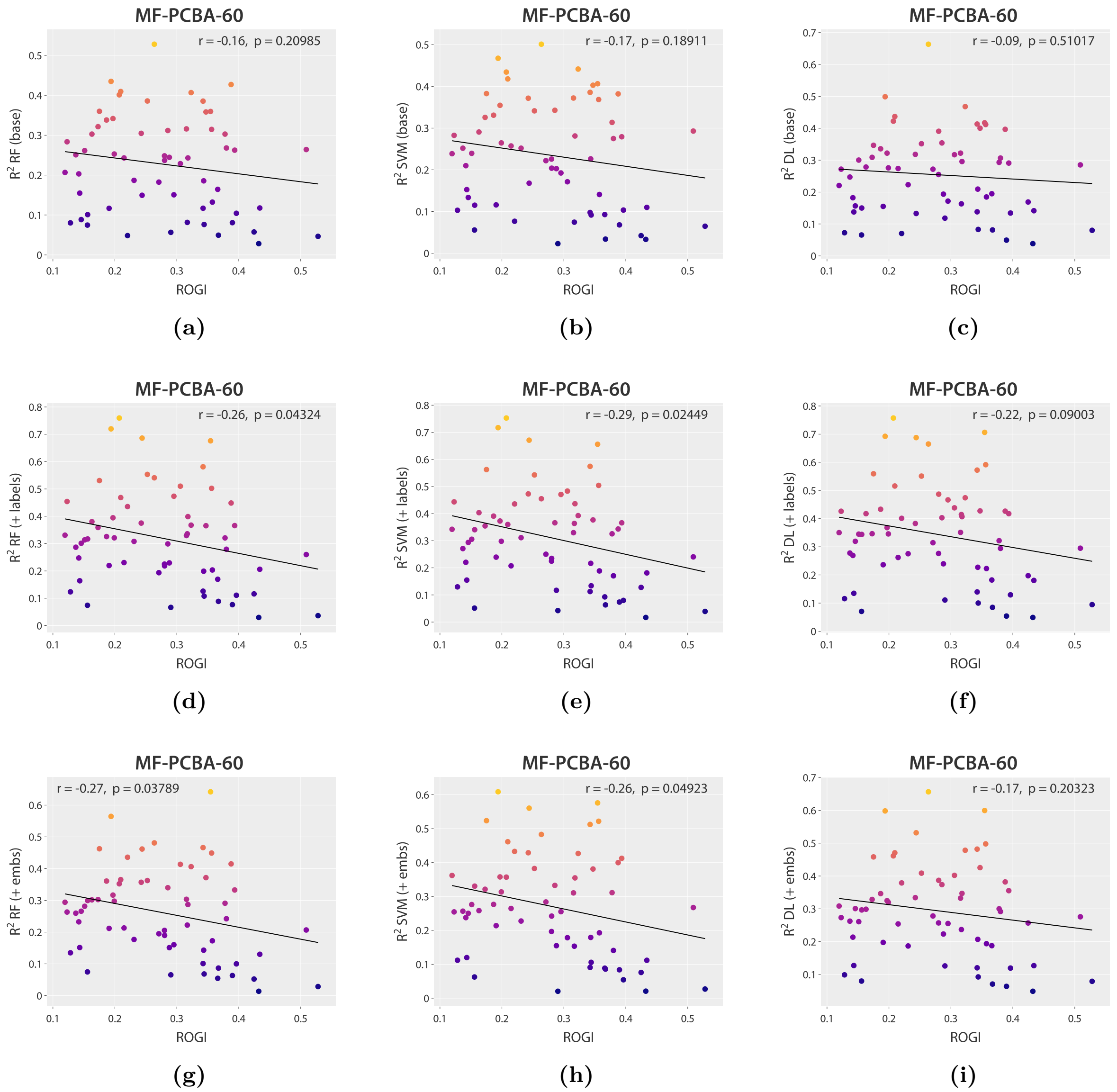

Figure S46. Plots of the ROGI (x-axis) against the R^2 (y-axis) for non-augmented (base), SD labels-augmented, and SD embeddings-augmented models (RF, SVM, and deep learning). All illustrations include the Pearson correlation coefficient (r). Brighter colours correspond to higher R^2 values.

SI 6 ΔR^2 versus ROGI

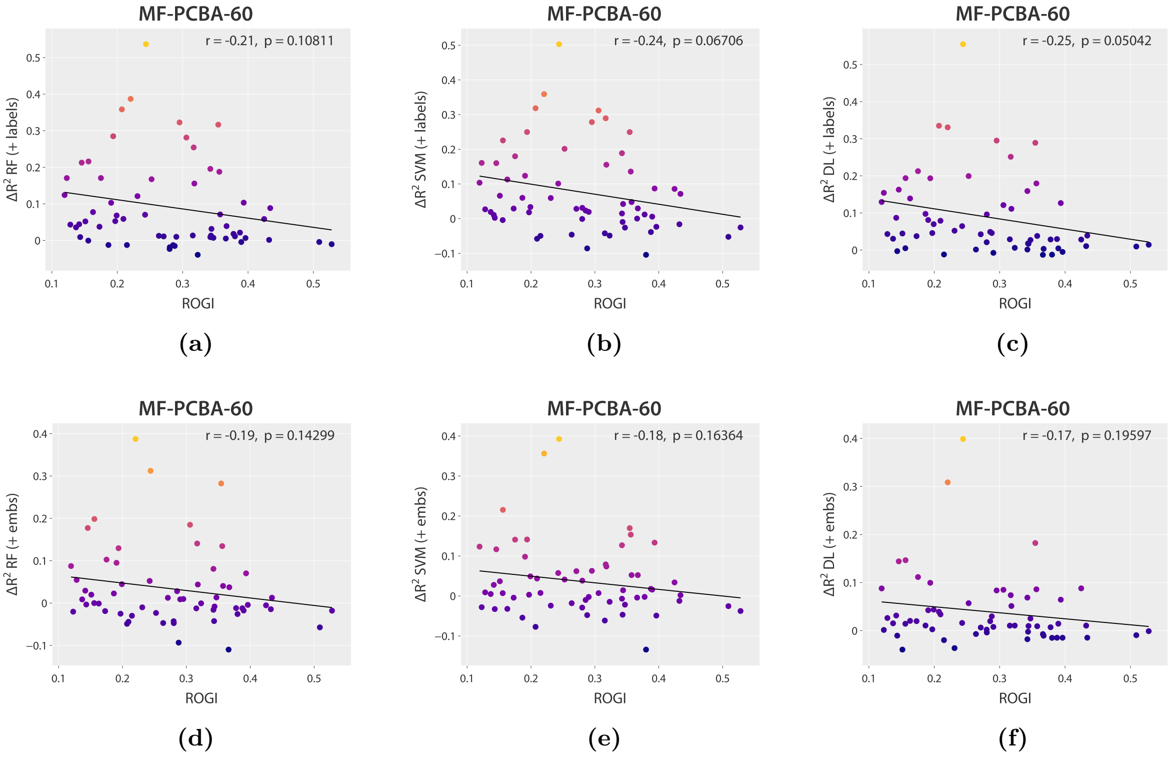

Figure S47. Plots of the ROGI (x-axis) against the ΔR^2 (y-axis) for RF, SVM, and deep learning models augmented with SD labels and SD embeddings. All illustrations include the Pearson correlation coefficient (r). Brighter colours correspond to higher ΔR^2 values.

SI 7 ROGI histogram

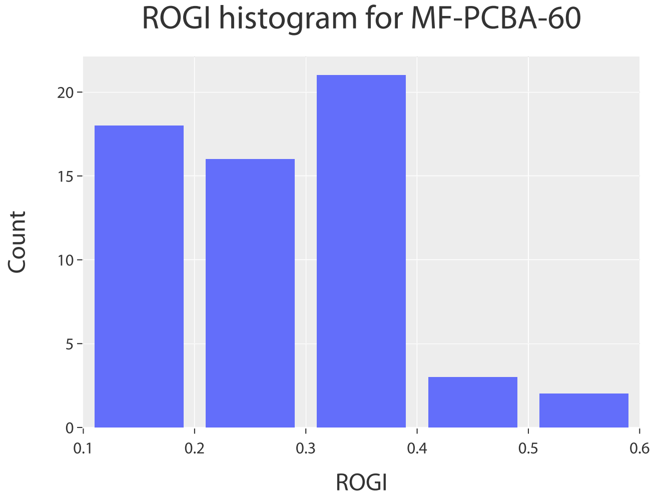

Figure S48. Histogram of the ROGI distribution for the 60 multi-fidelity datasets.

SI 8 Multiple linear regression with the ΔR^2 as the dependent variable

Table S7. Model summary for the RF model augmented with SD labels. Significance (**Sgn.**) codes: 0 '***' 0.001 '**' 0.01 '*' 0.05 '.' 0.1 ' ' 1.

Variable	Estimate	95% CI	*p* value	Sgn.
Intercept	-4.20×10^{-02}	[-1.72×10^{-01} , 8.76×10^{-02}]	5.19×10^{-01}	
Absolute SD/DR correlation	2.98×10^{-01}	[1.66×10^{-01} , 4.31×10^{-01}]	3.36×10^{-05}	***
# DR molecules	1.48×10^{-05}	[-2.69×10^{-05} , 5.66×10^{-05}]	4.80×10^{-01}	
# SD molecules	7.91×10^{-08}	[-2.84×10^{-07} , 4.42×10^{-07}]	6.64×10^{-01}	

Table S8. Model summary for the RF model augmented with SD embeddings. Significance (**Sgn.**) codes: 0 '***' 0.001 '**' 0.01 '*' 0.05 '.' 0.1 ' ' 1.

Variable	Estimate	95% CI	*p* value	Sgn.
Intercept	-3.33×10^{-02}	[-1.43×10^{-01} , 7.61×10^{-02}]	5.44×10^{-01}	
Absolute SD/DR correlation	1.54×10^{-01}	[4.27×10^{-02} , 2.66×10^{-01}]	7.64×10^{-03}	**
# DR molecules	9.04×10^{-06}	[-2.62×10^{-05} , 4.43×10^{-05}]	6.09×10^{-01}	
# SD molecules	2.57×10^{-08}	[-2.81×10^{-07} , 3.32×10^{-07}]	8.67×10^{-01}	

Table S9. Model summary for the SVM model augmented with SD labels. Significance (**Sgn.**) codes: 0 '***' 0.001 '**' 0.01 '*' 0.05 '.' 0.1 ' ' 1.

Variable	Estimate	95% CI	*p* value	Sgn.
Intercept	-1.66×10^{-02}	[-1.44×10^{-01} , 1.10×10^{-01}]	7.94×10^{-01}	
Absolute SD/DR correlation	3.31×10^{-01}	[2.01×10^{-01} , 4.61×10^{-01}]	4.02×10^{-06}	***
# DR molecules	5.92×10^{-06}	[-3.50×10^{-05} , 4.69×10^{-05}]	7.73×10^{-01}	
# SD molecules	-5.98×10^{-08}	[-4.16×10^{-07} , 2.96×10^{-07}]	7.37×10^{-01}	

Table S10. Model summary for the SVM model augmented with SD embeddings. Significance (**Sgn.**) codes: 0 '***' 0.001 '**' 0.01 '*' 0.05 '.' 0.1 ' ' 1.

Variable	Estimate	95% CI	*p* value	Sgn.
Intercept	-3.96×10^{-02}	[-1.45×10^{-01} , 6.54×10^{-02}]	4.53×10^{-01}	
Absolute SD/DR correlation	1.73×10^{-01}	[6.55×10^{-02} , 2.80×10^{-01}]	2.10×10^{-03}	**
# DR molecules	1.39×10^{-05}	[-1.99×10^{-05} , 4.77×10^{-05}]	4.14×10^{-01}	
# SD molecules	1.53×10^{-08}	[-2.79×10^{-07} , 3.09×10^{-07}]	9.18×10^{-01}	

Table S11. Model summary for the deep learning model augmented with SD labels. Significance (**Sgn.**) codes: 0 '***' 0.001 '**' 0.01 '*' 0.05 '.' 0.1 ' ' 1.

**Variable**	**Estimate**	**95% CI**	***p* value**	**Sgn.**
Intercept	-9.64×10^{-03}	$[-1.24 \times 10^{-01}, 1.05 \times 10^{-01}]$	8.67×10^{-01}	
Absolute SD/DR correlation	2.72×10^{-01}	$[1.55 \times 10^{-01}, 3.89 \times 10^{-01}]$	1.98×10^{-05}	***
# DR molecules	-5.32×10^{-06}	$[-4.22 \times 10^{-05}, 3.15 \times 10^{-05}]$	7.73×10^{-01}	
# SD molecules	8.21×10^{-08}	$[-2.38 \times 10^{-07}, 4.03 \times 10^{-07}]$	6.10×10^{-01}	

Table S12. Model summary for the deep learning model augmented with SD embeddings. Significance (**Sgn.**) codes: 0 '***' 0.001 '**' 0.01 '*' 0.05 '.' 0.1 ' ' 1.

**Variable**	**Estimate**	**95% CI**	***p* value**	**Sgn.**
Intercept	-2.04×10^{-02}	$[-1.08 \times 10^{-01}, 6.67 \times 10^{-02}]$	6.40×10^{-01}	
Absolute SD/DR correlation	1.23×10^{-01}	$[3.36 \times 10^{-02}, 2.12 \times 10^{-01}]$	7.82×10^{-03}	**
# DR molecules	-1.23×10^{-08}	$[-2.81 \times 10^{-05}, 2.81 \times 10^{-05}]$	9.99×10^{-01}	
# SD molecules	8.11×10^{-08}	$[-1.63 \times 10^{-07}, 3.25 \times 10^{-07}]$	5.09×10^{-01}	

SI 9 Multiple linear regression including the ROGI

Table S13. Model summary for the RF model augmented with SD labels. Significance (**Sgn.**) codes: 0 '***' 0.001 '**' 0.01 '*' 0.05 '.' 0.1 ' ' 1.

**Variable**	**Estimate**	**95% CI**	***p* value**	**Sgn.**
Intercept	-2.82×10^{-02}	$[-1.89 \times 10^{-01}, 1.33 \times 10^{-01}]$	7.27×10^{-01}	
Absolute SD/DR correlation	2.91×10^{-01}	$[1.48 \times 10^{-01}, 4.34 \times 10^{-01}]$	1.52×10^{-04}	***
# DR molecules	1.42×10^{-05}	$[-2.81 \times 10^{-05}, 5.65 \times 10^{-05}]$	5.04×10^{-01}	
# SD molecules	8.49×10^{-08}	$[-2.83 \times 10^{-07}, 4.53 \times 10^{-07}]$	6.46×10^{-01}	
ROGI	-4.43×10^{-02}	$[-3.42 \times 10^{-01}, 2.53 \times 10^{-01}]$	7.67×10^{-01}	

Table S14. Model summary for the RF model augmented with SD embeddings. Significance (**Sgn.**) codes: 0 '***' 0.001 '**' 0.01 '*' 0.05 '.' 0.1 ' ' 1.

**Variable**	**Estimate**	**95% CI**	***p* value**	**Sgn.**
Intercept	-9.85×10^{-03}	$[-1.45 \times 10^{-01}, 1.25 \times 10^{-01}]$	8.85×10^{-01}	
Absolute SD/DR correlation	1.42×10^{-01}	$[2.11 \times 10^{-02}, 2.62 \times 10^{-01}]$	2.21×10^{-02}	*
# DR molecules	8.00×10^{-06}	$[-2.76 \times 10^{-05}, 4.36 \times 10^{-05}]$	6.55×10^{-01}	
# SD molecules	3.55×10^{-08}	$[-2.75 \times 10^{-07}, 3.46 \times 10^{-07}]$	8.19×10^{-01}	
ROGI	-7.48×10^{-02}	$[-3.25 \times 10^{-01}, 1.76 \times 10^{-01}]$	5.52×10^{-01}	

Table S15. Model summary for the SVM model augmented with SD labels. Significance (**Sgn.**) codes: 0 '***' 0.001 '**' 0.01 '*' 0.05 '.' 0.1 ' ' 1.

**Variable**	**Estimate**	**95% CI**	***p* value**	**Sgn.**
Intercept	8.51×10^{-04}	[-1.57×10^{-01} , 1.58×10^{-01}]	9.91×10^{-01}	
Absolute SD/DR correlation	3.22×10^{-01}	[1.82×10^{-01} , 4.62×10^{-01}]	2.54×10^{-05}	***
# DR molecules	5.14×10^{-06}	[-3.63×10^{-05} , 4.66×10^{-05}]	8.05×10^{-01}	
# SD molecules	-5.26×10^{-08}	[-4.13×10^{-07} , 3.08×10^{-07}]	7.71×10^{-01}	
ROGI	-5.56×10^{-02}	[-3.47×10^{-01} , 2.36×10^{-01}]	7.03×10^{-01}	

Table S16. Model summary for the SVM model augmented with SD embeddings. Significance (**Sgn.**) codes: 0 '***' 0.001 '**' 0.01 '*' 0.05 '.' 0.1 ' ' 1.

**Variable**	**Estimate**	**95% CI**	***p* value**	**Sgn.**
Intercept	-2.61×10^{-02}	[-1.56×10^{-01} , 1.04×10^{-01}]	6.89×10^{-01}	
Absolute SD/DR correlation	1.65×10^{-01}	[4.95×10^{-02} , 2.81×10^{-01}]	5.99×10^{-03}	**
# DR molecules	1.33×10^{-05}	[-2.10×10^{-05} , 4.76×10^{-05}]	4.41×10^{-01}	
# SD molecules	2.09×10^{-08}	[-2.77×10^{-07} , 3.19×10^{-07}]	8.89×10^{-01}	
ROGI	-4.32×10^{-02}	[-2.84×10^{-01} , 1.98×10^{-01}]	7.21×10^{-01}	

Table S17. Model summary for the deep learning model augmented with SD labels. Significance (**Sgn.**) codes: 0 '***' 0.001 '**' 0.01 '*' 0.05 '.' 0.1 ' ' 1.

**Variable**	**Estimate**	**95% CI**	***p* value**	**Sgn.**
Intercept	2.19×10^{-02}	[-1.19×10^{-01} , 1.63×10^{-01}]	7.57×10^{-01}	
Absolute SD/DR correlation	2.55×10^{-01}	[1.29×10^{-01} , 3.81×10^{-01}]	1.56×10^{-04}	***
# DR molecules	-6.73×10^{-06}	[-4.39×10^{-05} , 3.05×10^{-05}]	7.18×10^{-01}	
# SD molecules	9.53×10^{-08}	[-2.28×10^{-07} , 4.19×10^{-07}]	5.57×10^{-01}	
ROGI	-1.00×10^{-01}	[-3.62×10^{-01} , 1.61×10^{-01}]	4.44×10^{-01}	

Table S18. Model summary for the deep learning model augmented with SD embeddings. Significance (**Sgn.**) codes: 0 '***' 0.001 '**' 0.01 '*' 0.05 '.' 0.1 ' ' 1.

**Variable**	**Estimate**	**95% CI**	***p* value**	**Sgn.**
Intercept	-4.87×10^{-03}	[-1.13×10^{-01} , 1.03×10^{-01}]	9.28×10^{-01}	
Absolute SD/DR correlation	1.14×10^{-01}	[1.81×10^{-02} , 2.10×10^{-01}]	2.08×10^{-02}	*
# DR molecules	-7.06×10^{-07}	[-2.91×10^{-05} , 2.77×10^{-05}]	9.60×10^{-01}	
# SD molecules	8.76×10^{-08}	[-1.60×10^{-07} , 3.35×10^{-07}]	4.81×10^{-01}	
ROGI	-4.96×10^{-02}	[-2.49×10^{-01} , 1.50×10^{-01}]	6.21×10^{-01}	

SI 10 Example PubChem search

The PubChem database was searched through the website https://www.ncbi.nlm.nih.gov/pcassay/limits. The search used a count of tested substances from 500 to 20,000, with a Substance Type of 'Chemical', Screening Stage of 'Confirmatory, Dose-Response', Target of 'Single', and Target Type of 'Protein Target' returns 407 results. This example search was performed on 17 January 2023.

SI 11 Augmenting with SD fingerprints

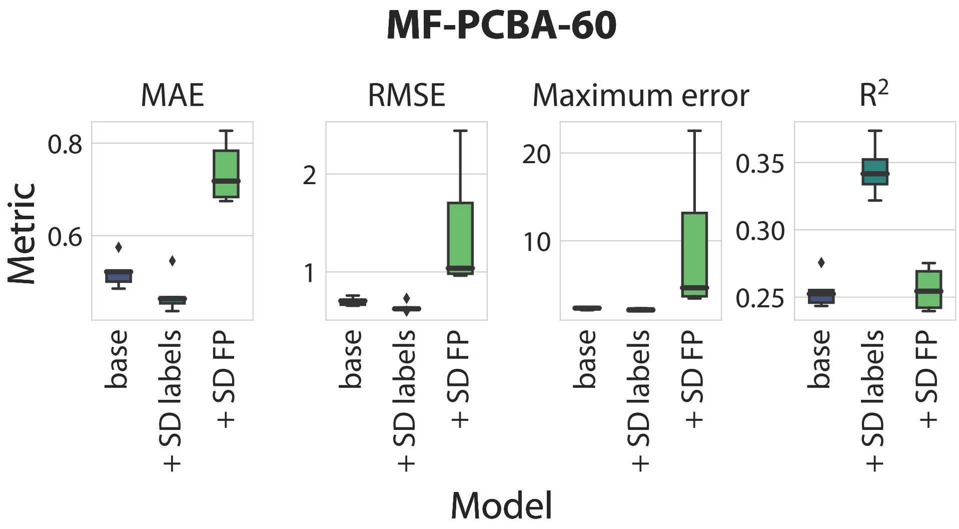

Figure S49. Test MAE on MF-PCBA-60.

Figure S50. The predictive performance on MF-PCBA-60 for deep learning models. The SD fingerprints ('+SD FP)' augmentation is illustrated alongside the already presented metrics for the baseline and SD labels-augmented deep learning models.

SI 12 Augmenting with SD fingerprints – Individual plots

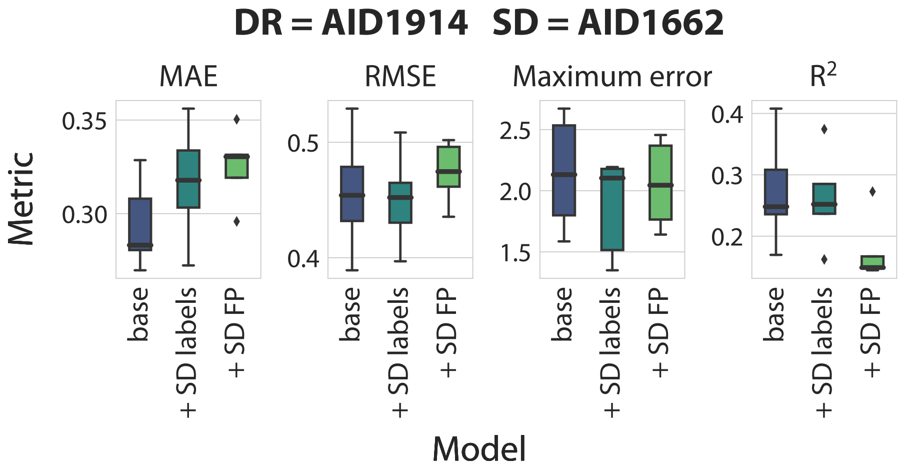

Figure S51. Test MAE, RMSE, maximum error, and R^2 for AID1914 – AID1662 for deep learning.

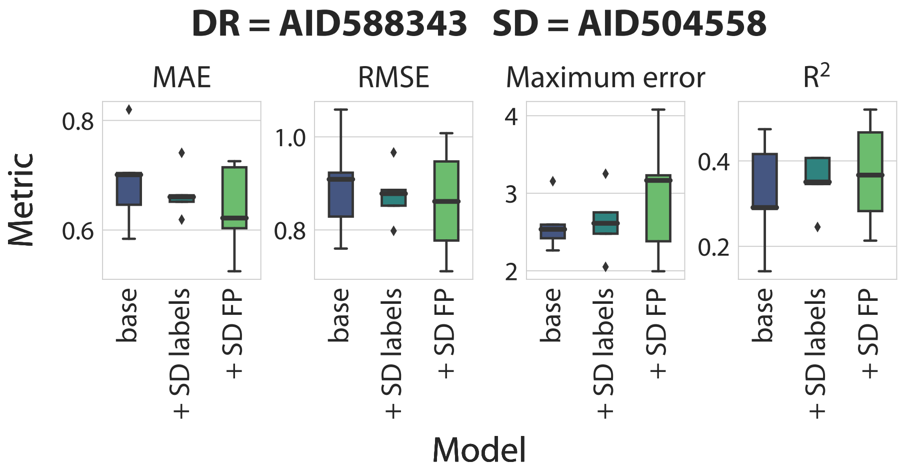

Figure S52. Test MAE, RMSE, maximum error, and R^2 for AID588343 – AID504558 for deep learning.

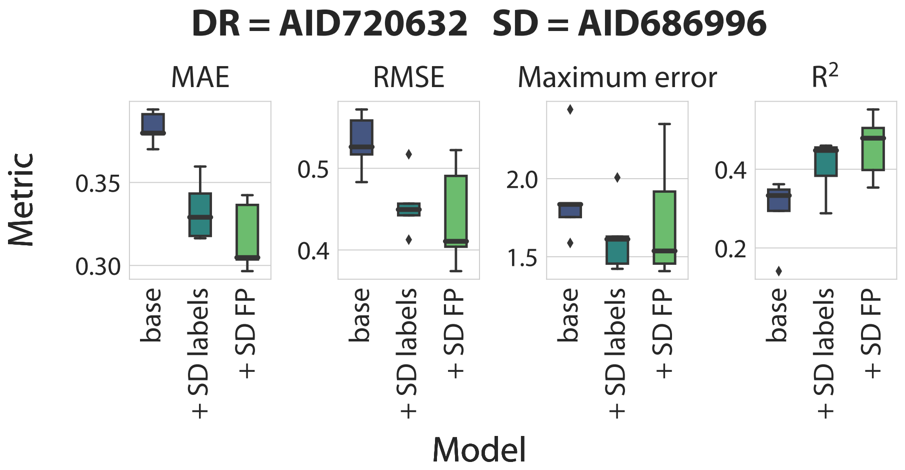

Figure S53. Test MAE, RMSE, maximum error, and R^2 for AID720632 – AID686996 for deep learning.

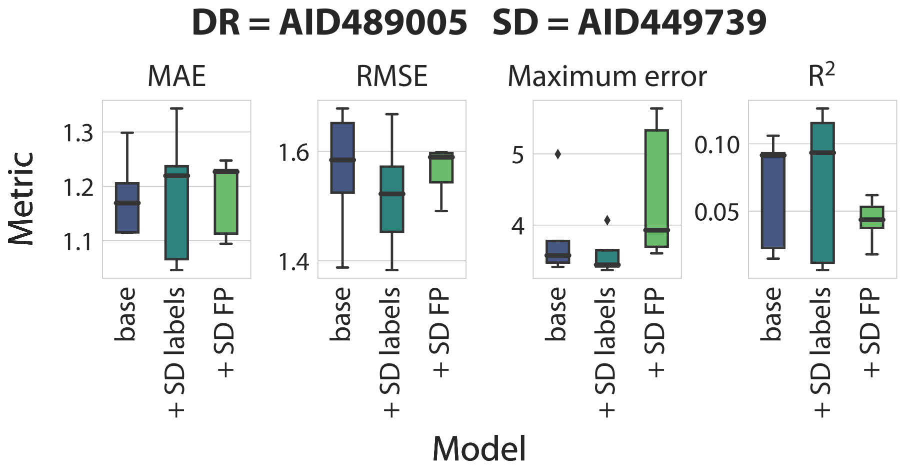

Figure S54. Test MAE, RMSE, maximum error, and R^2 for AID489005 – AID449739 for deep learning.

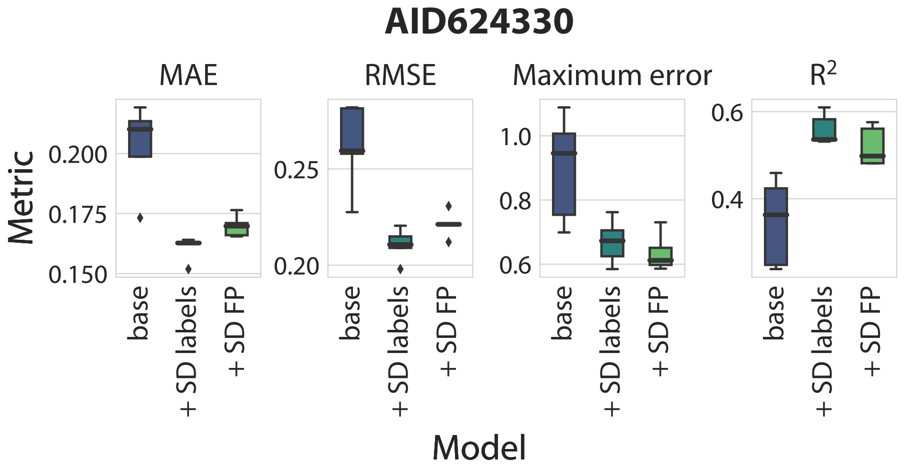

Figure S55. Test MAE, RMSE, maximum error, and R^2 for AID624330 – AID624330 for deep learning.

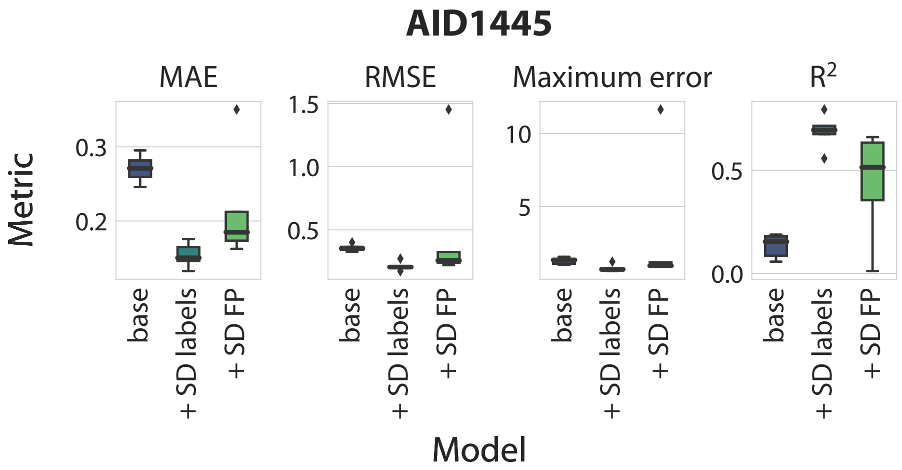

Figure S56. Test MAE, RMSE, maximum error, and R^2 for AID1445 – AID1445 for deep learning.

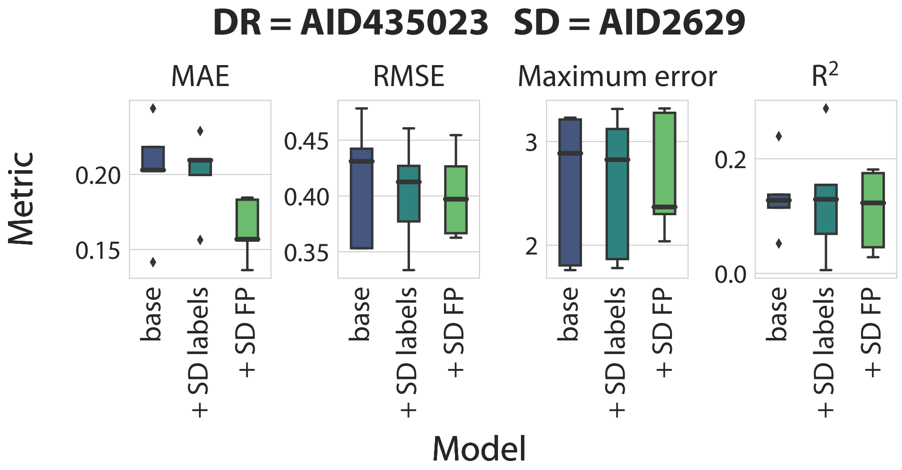

Figure S57. Test MAE, RMSE, maximum error, and R^2 for AID435023 – AID2629 for deep learning.

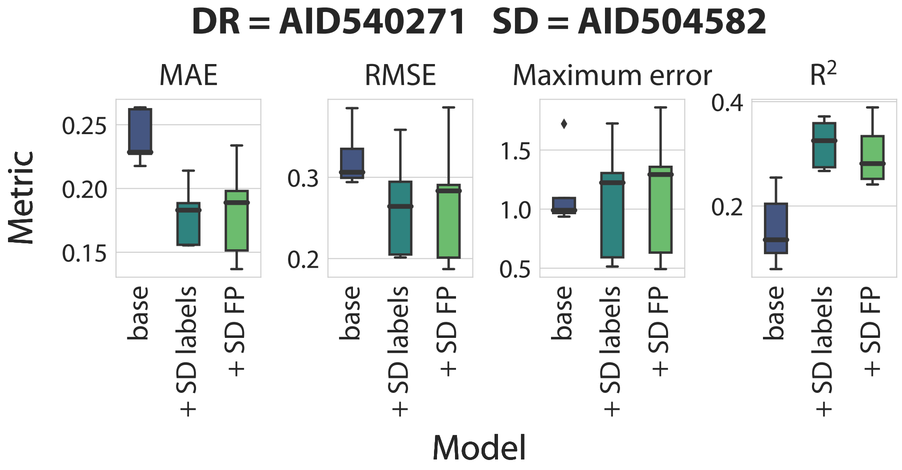

Figure S58. Test MAE, RMSE, maximum error, and R^2 for AID540271 – AID504582 for deep learning.

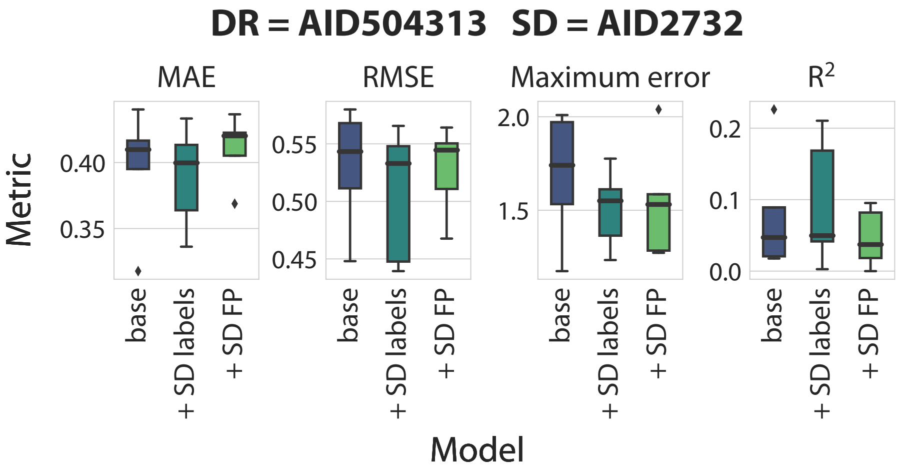

Figure S59. Test MAE, RMSE, maximum error, and R^2 for AID504313 – AID2732 for deep learning.

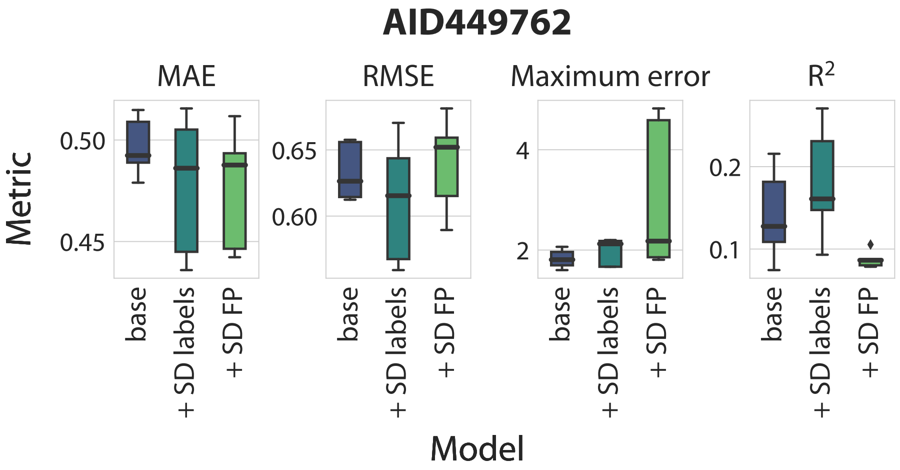

Figure S60. Test MAE, RMSE, maximum error, and R^2 for AID449762 – AID449762 for deep learning.

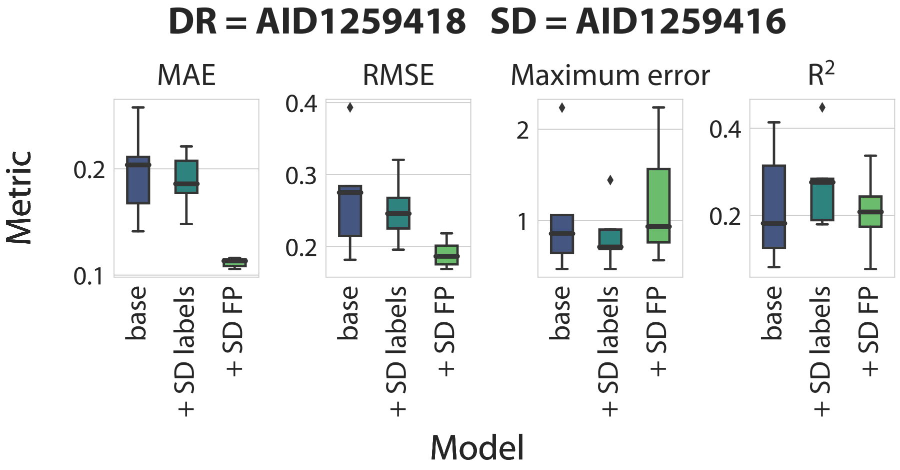

Figure S61. Test MAE, RMSE, maximum error, and R^2 for AID1259418 – AID1259416 for deep learning.

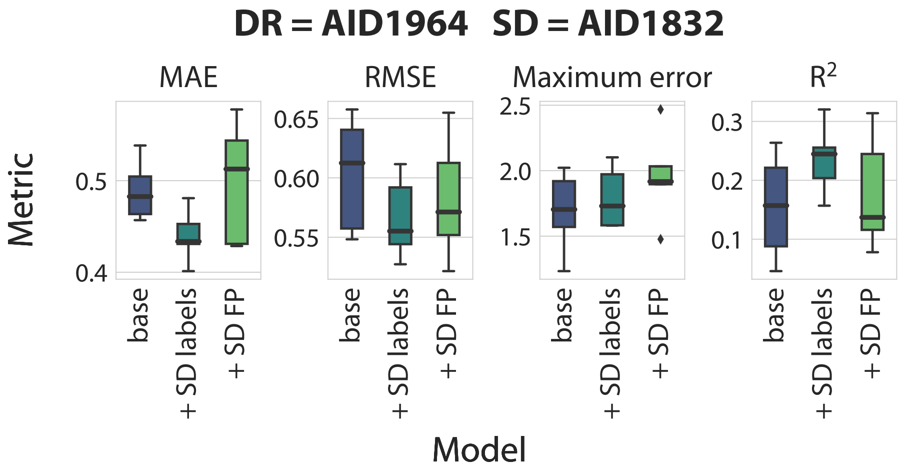

Figure S62. Test MAE, RMSE, maximum error, and R^2 for AID1964 – AID1832 for deep learning.

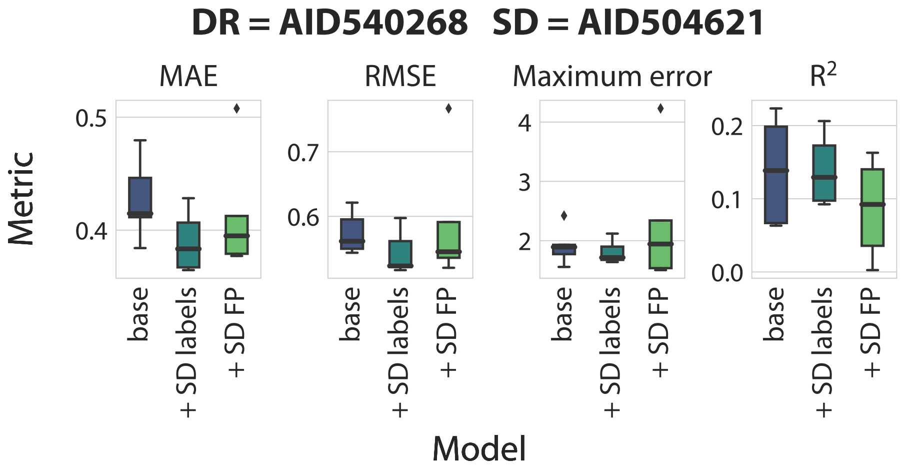

Figure S63. Test MAE, RMSE, maximum error, and R^2 for AID540268 – AID504621 for deep learning.

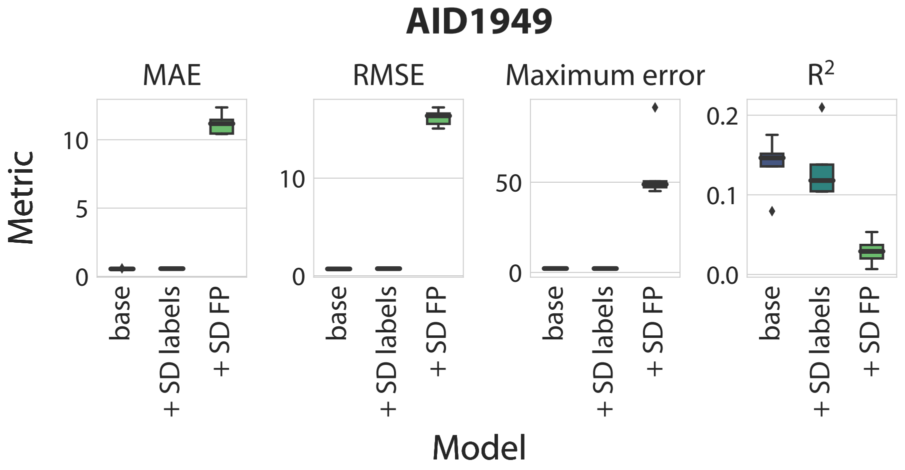

Figure S64. Test MAE, RMSE, maximum error, and R^2 for AID1949 – AID1949 for deep learning.

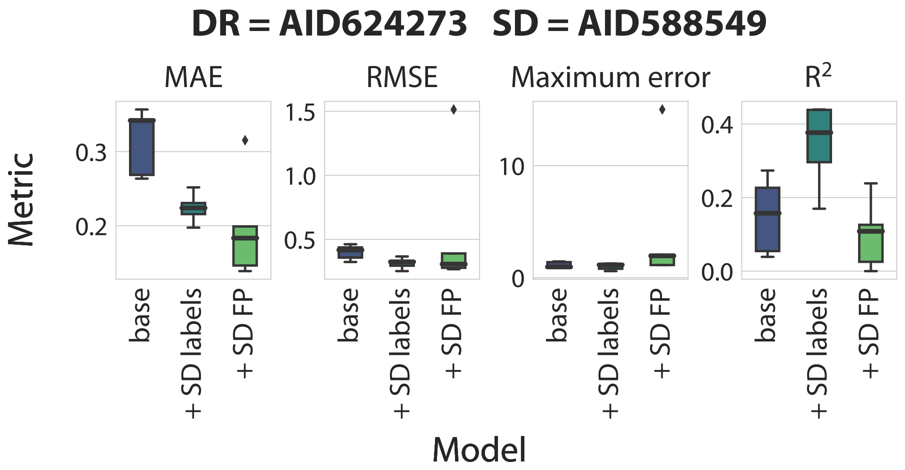

Figure S65. Test MAE, RMSE, maximum error, and R^2 for AID624273 – AID588549 for deep learning.

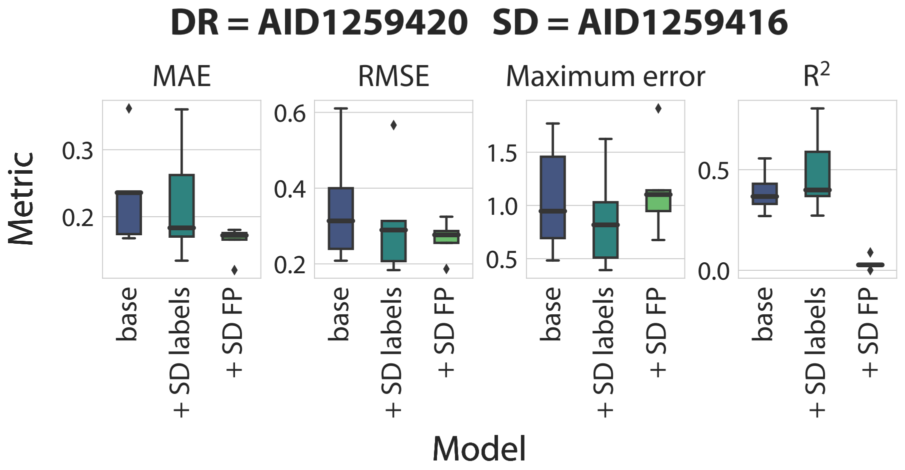

Figure S66. Test MAE, RMSE, maximum error, and R^2 for AID1259420 – AID1259416 for deep learning.

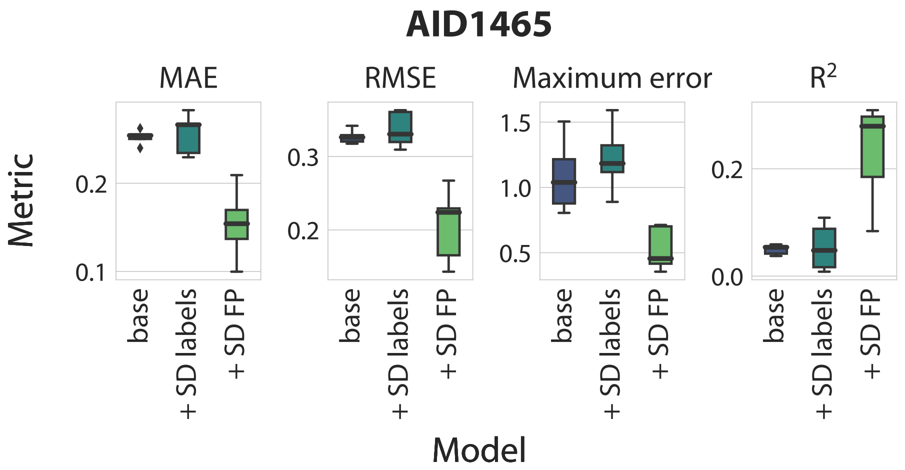

Figure S67. Test MAE, RMSE, maximum error, and R^2 for AID1465 – AID1465 for deep learning.

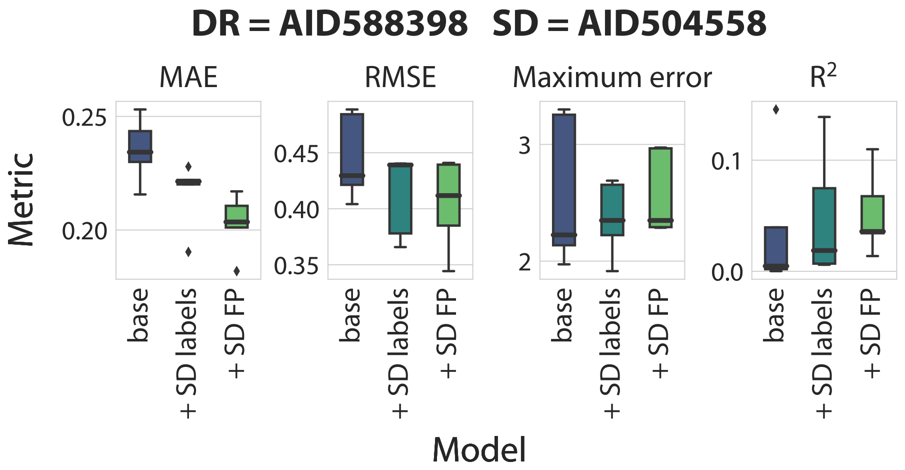

Figure S68. Test MAE, RMSE, maximum error, and R^2 for AID588398 – AID504558 for deep learning.

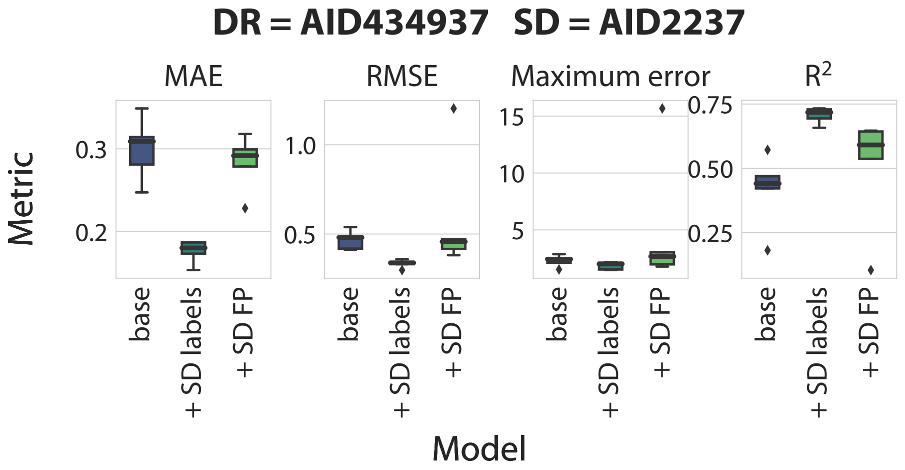

Figure S69. Test MAE, RMSE, maximum error, and R^2 for AID434937 – AID2237 for deep learning.

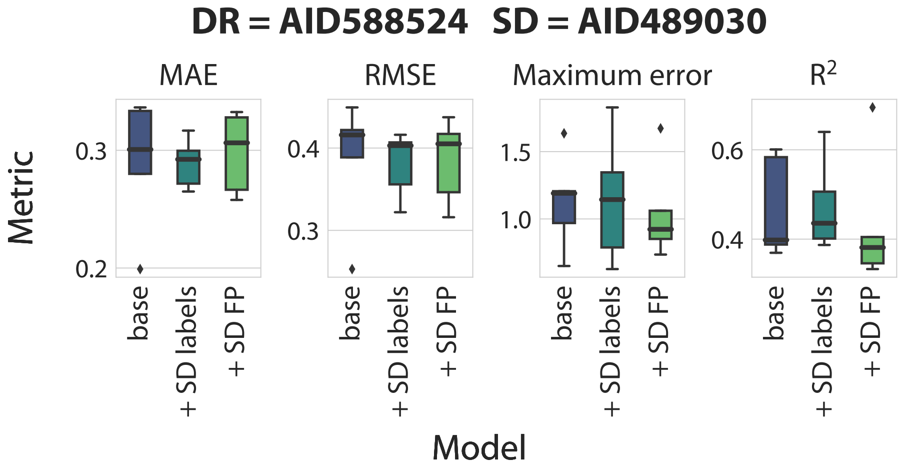

Figure S70. Test MAE, RMSE, maximum error, and R^2 for AID588524 – AID489030 for deep learning.

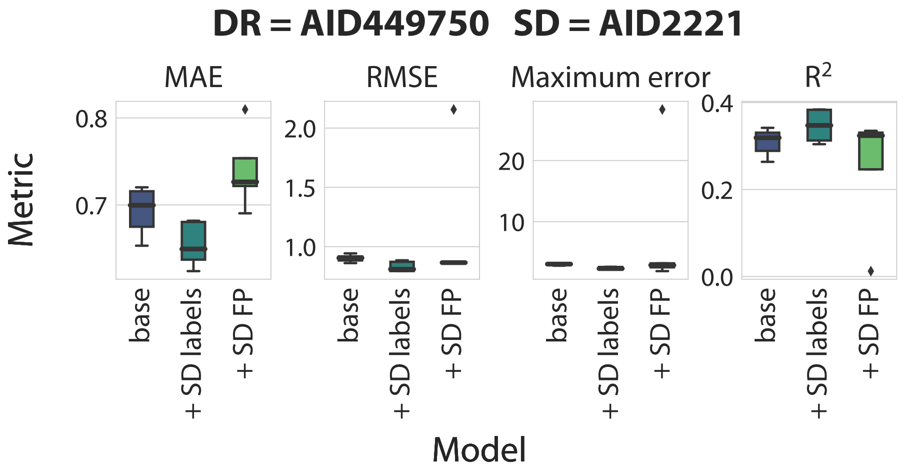

Figure S71. Test MAE, RMSE, maximum error, and R^2 for AID449750 – AID2221 for deep learning.

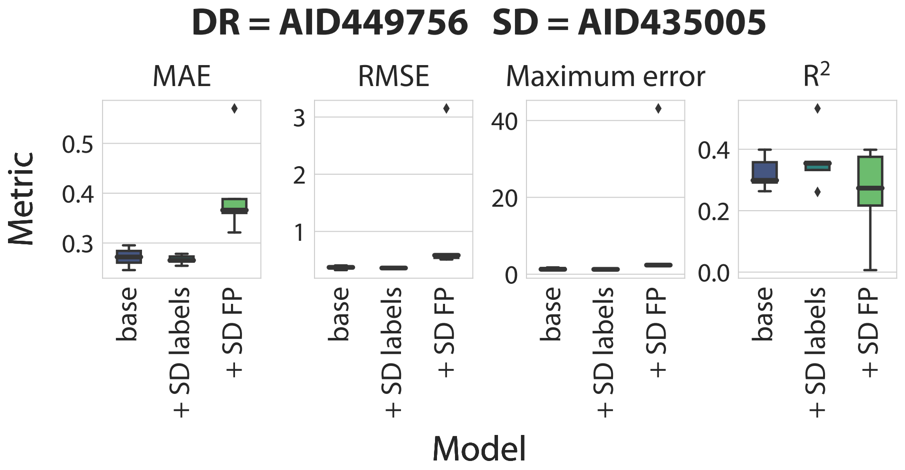

Figure S72. Test MAE, RMSE, maximum error, and R^2 for AID449756 – AID435005 for deep learning.

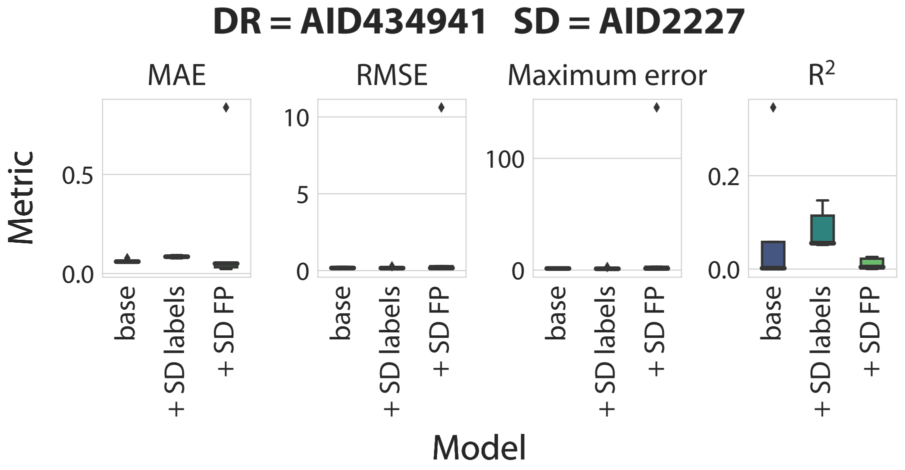

Figure S73. Test MAE, RMSE, maximum error, and R^2 for AID434941 – AID2227 for deep learning.

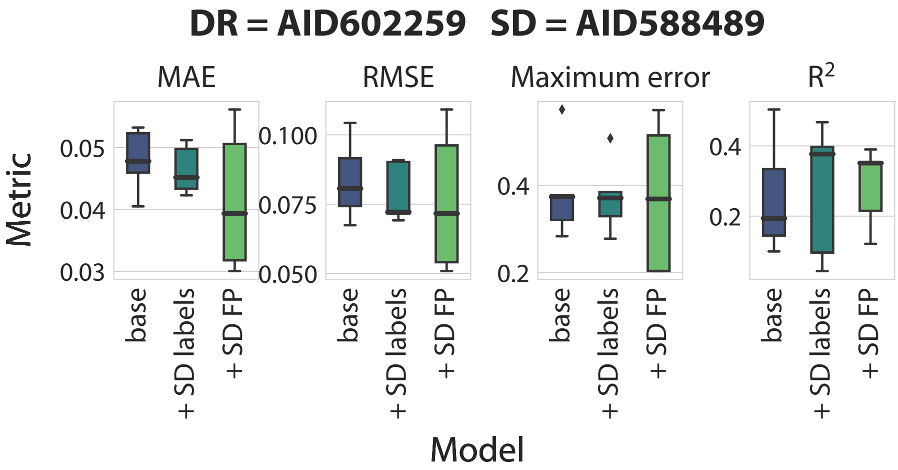

Figure S74. Test MAE, RMSE, maximum error, and R^2 for AID602259 – AID588489 for deep learning.

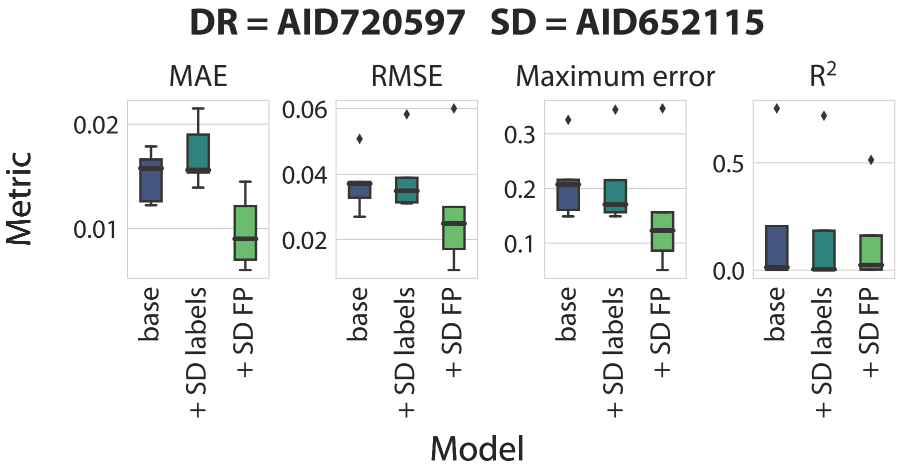

Figure S75. Test MAE, RMSE, maximum error, and R^2 for AID720597 – AID652115 for deep learning.

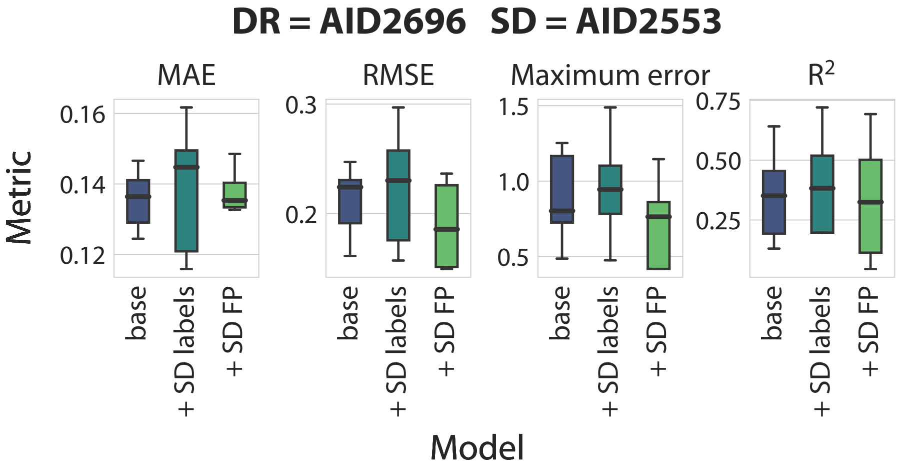

Figure S76. Test MAE, RMSE, maximum error, and R^2 for AID2696 – AID2553 for deep learning.

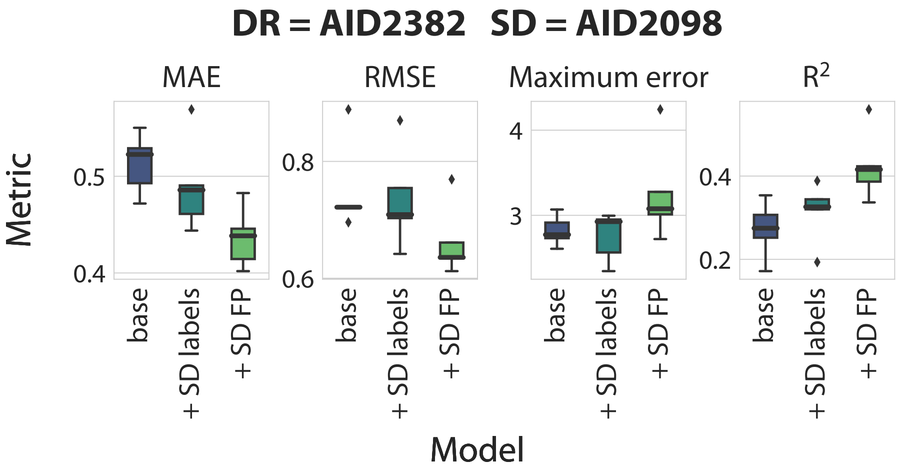

Figure S77. Test MAE, RMSE, maximum error, and R^2 for AID2382 – AID2098 for deep learning.

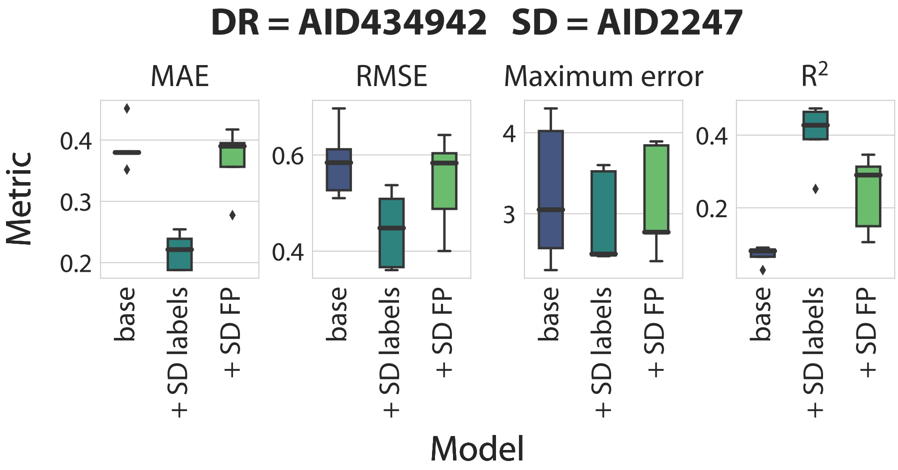

Figure S78. Test MAE, RMSE, maximum error, and R^2 for AID434942 – AID2247 for deep learning.

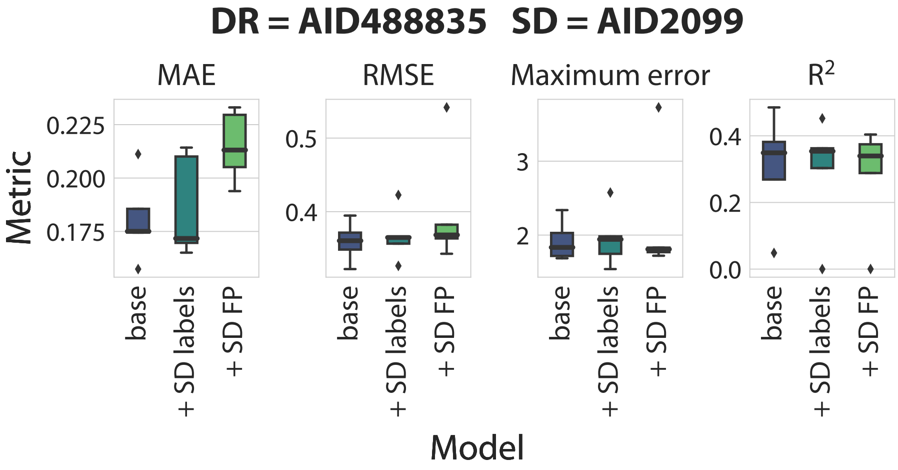

Figure S79. Test MAE, RMSE, maximum error, and R^2 for AID488835 – AID2099 for deep learning.

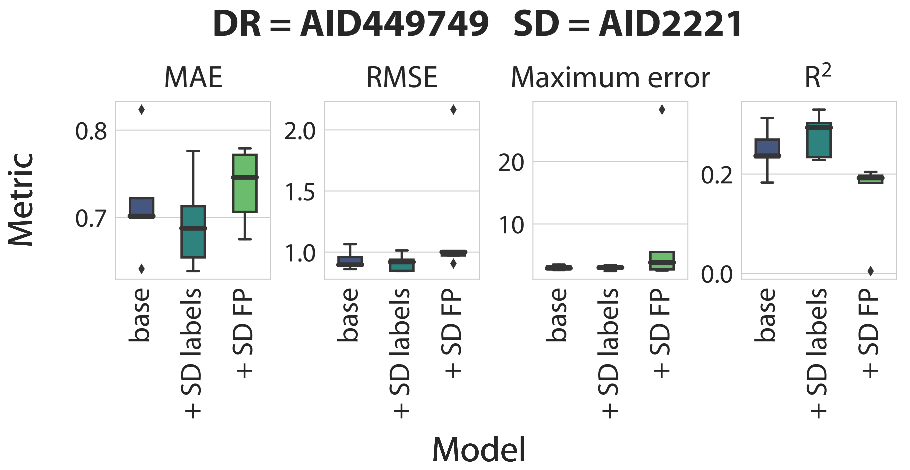

Figure S80. Test MAE, RMSE, maximum error, and R^2 for AID449749 – AID2221 for deep learning.

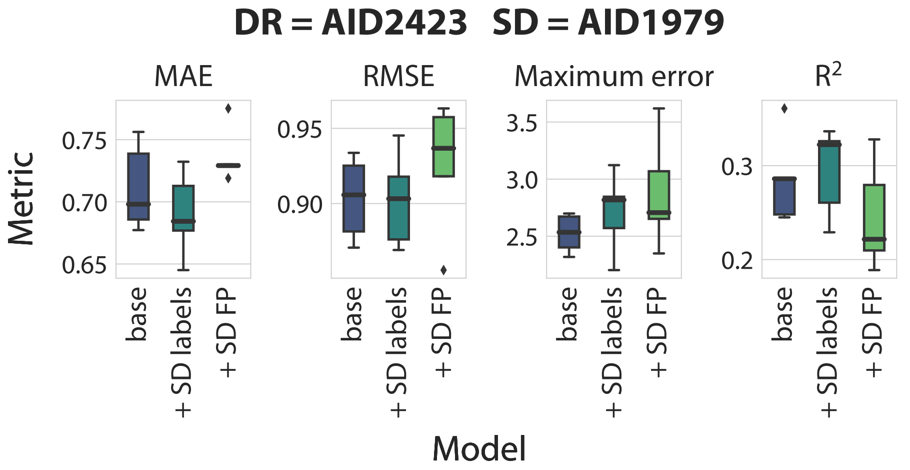

Figure S81. Test MAE, RMSE, maximum error, and R^2 for AID2423 – AID1979 for deep learning.

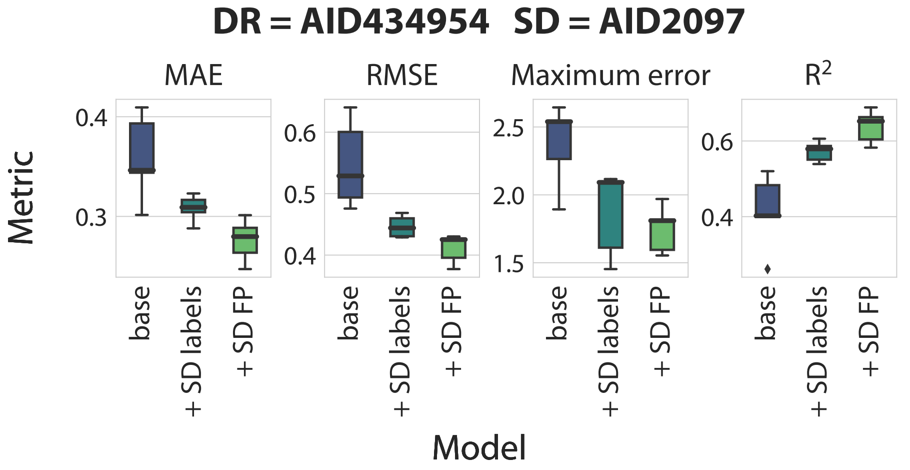

Figure S82. Test MAE, RMSE, maximum error, and R^2 for AID434954 – AID2097 for deep learning.

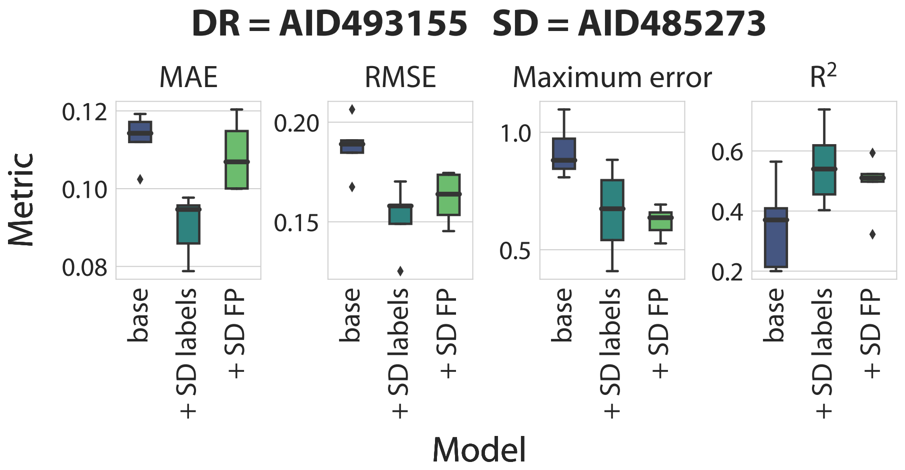

Figure S83. Test MAE, RMSE, maximum error, and R^2 for AID493155 – AID485273 for deep learning.

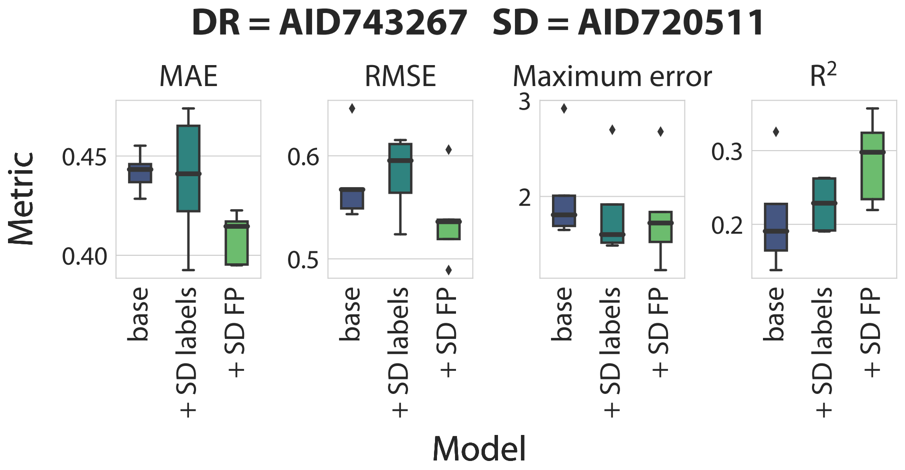

Figure S84. Test MAE, RMSE, maximum error, and R^2 for AID743267 – AID720511 for deep learning.

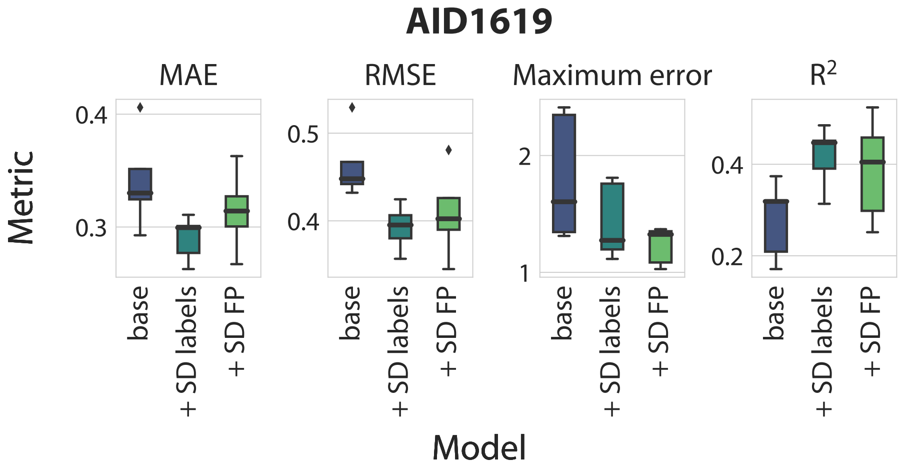

Figure S85. Test MAE, RMSE, maximum error, and R^2 for AID1619 – AID1619 for deep learning.

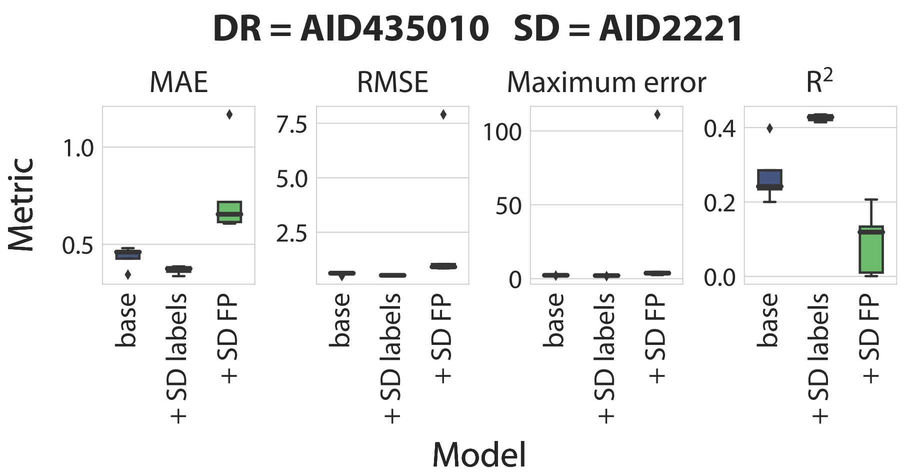

Figure S86. Test MAE, RMSE, maximum error, and R^2 for AID435010 – AID2221 for deep learning.

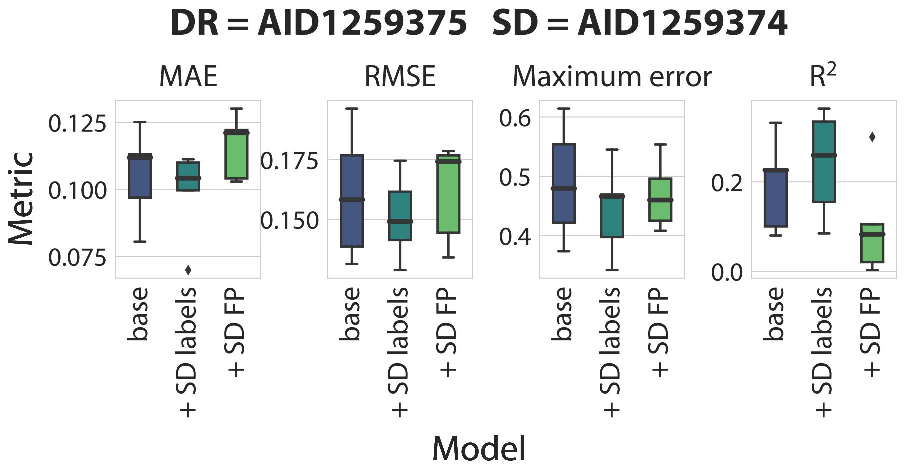

Figure S87. Test MAE, RMSE, maximum error, and R^2 for AID1259375 – AID1259374 for deep learning.

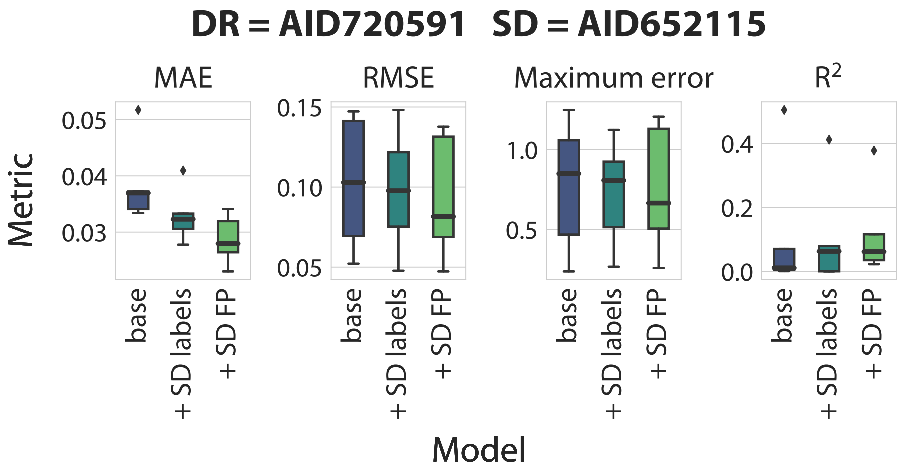

Figure S88. Test MAE, RMSE, maximum error, and R^2 for AID720591 – AID652115 for deep learning.

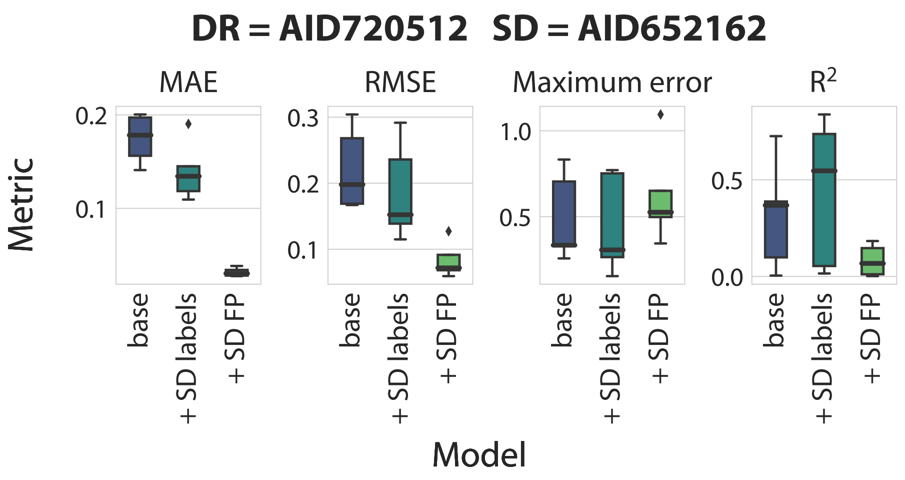

Figure S89. Test MAE, RMSE, maximum error, and R^2 for AID720512 – AID652162 for deep learning.

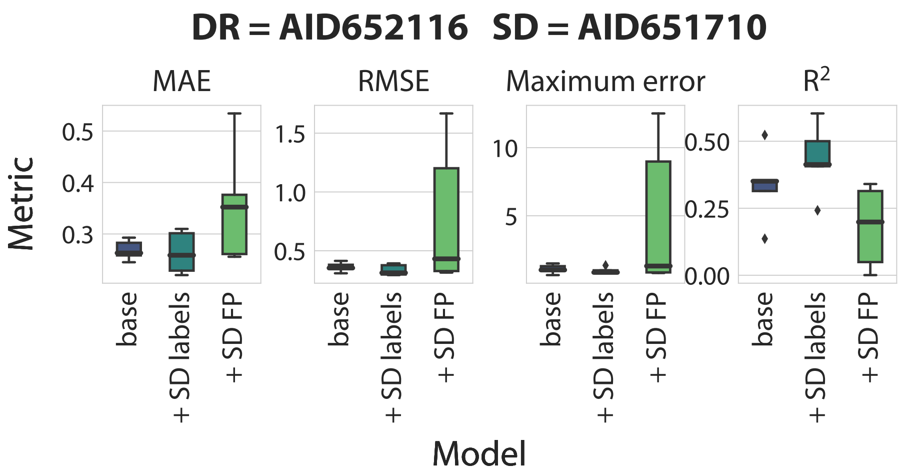

Figure S90. Test MAE, RMSE, maximum error, and R^2 for AID652116 – AID651710 for deep learning.

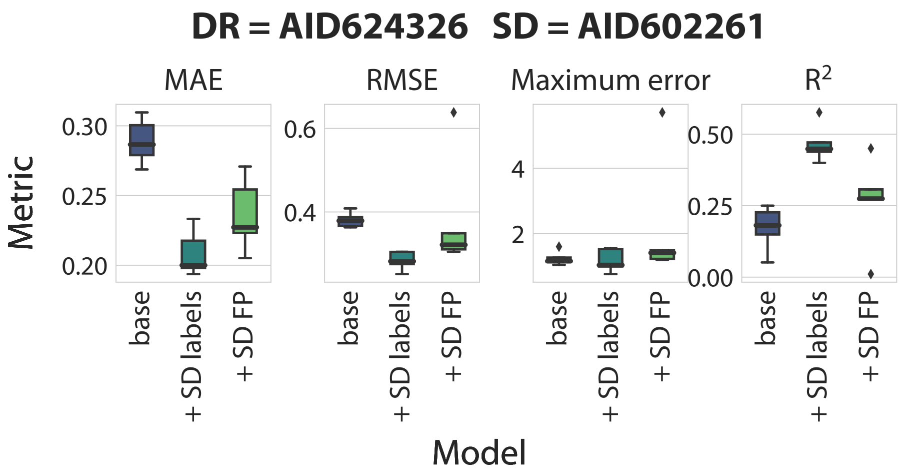

Figure S91. Test MAE, RMSE, maximum error, and R^2 for AID624326 – AID602261 for deep learning.

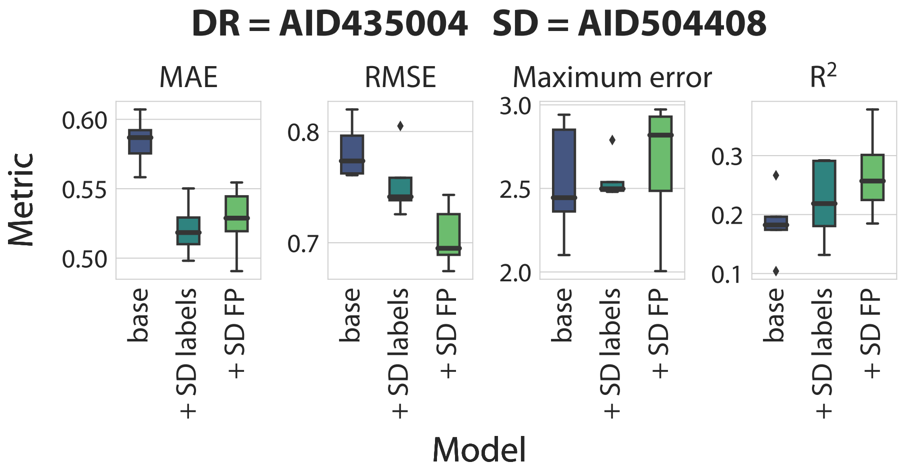

Figure S92. Test MAE, RMSE, maximum error, and R^2 for AID435004 – AID504408 for deep learning.

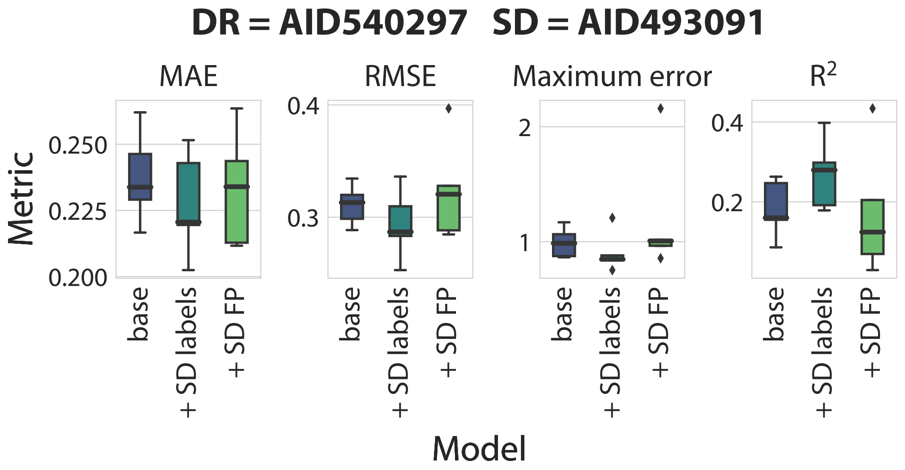

Figure S93. Test MAE, RMSE, maximum error, and R^2 for AID540297 – AID493091 for deep learning.

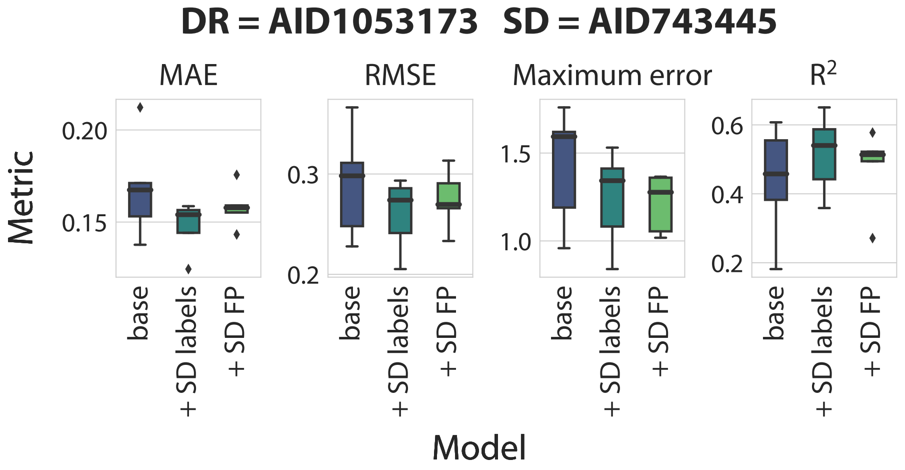

Figure S94. Test MAE, RMSE, maximum error, and R^2 for AID1053173 – AID743445 for deep learning.

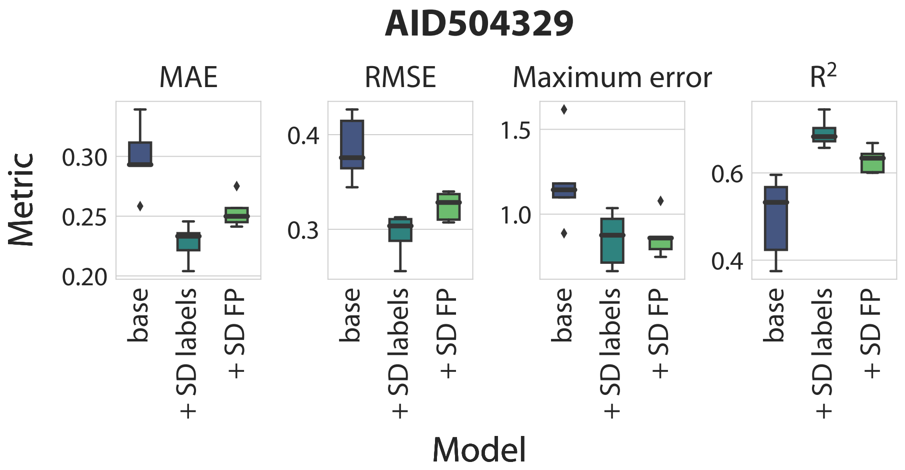

Figure S95. Test MAE, RMSE, maximum error, and R^2 for AID504329 – AID504329 for deep learning.

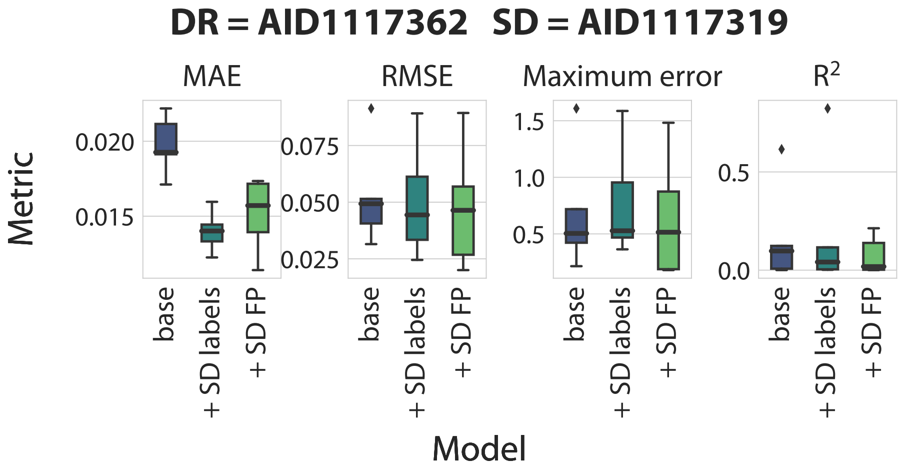

Figure S96. Test MAE, RMSE, maximum error, and R^2 for AID1117362 – AID1117319 for deep learning.

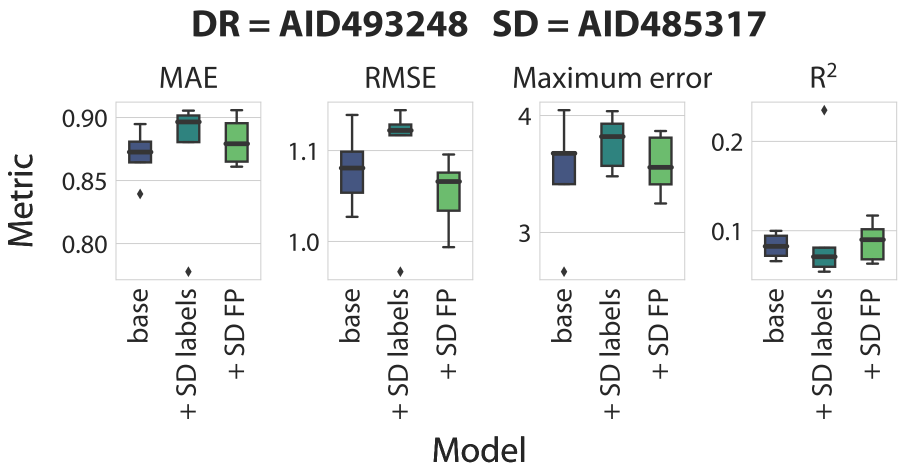

Figure S97. Test MAE, RMSE, maximum error, and R^2 for AID493248 – AID485317 for deep learning.

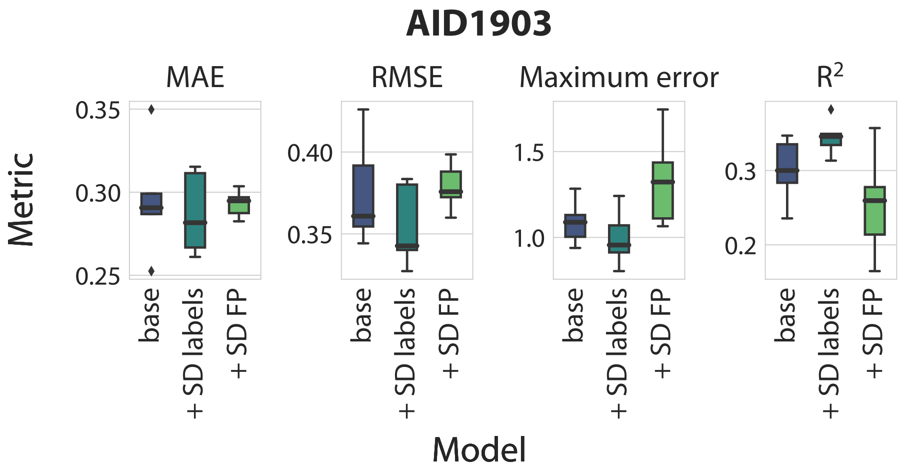

Figure S98. Test MAE, RMSE, maximum error, and R^2 for AID1903 – AID1903 for deep learning.

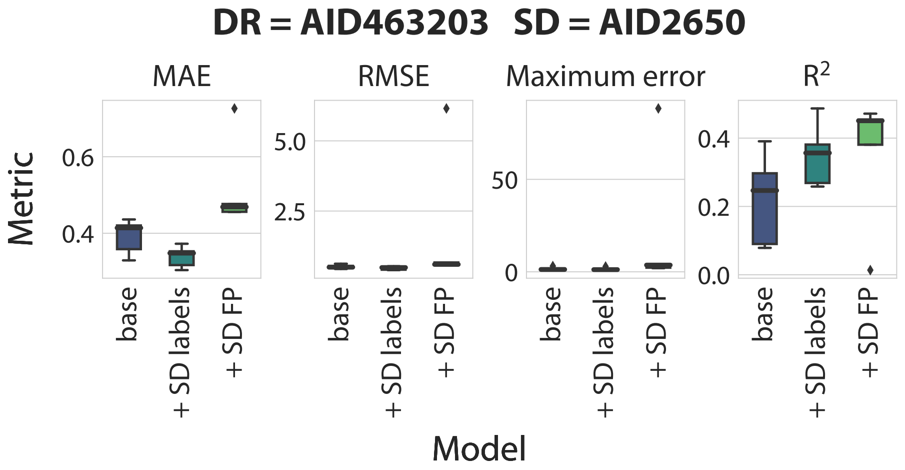

Figure S99. Test MAE, RMSE, maximum error, and R^2 for AID463203 – AID2650 for deep learning.

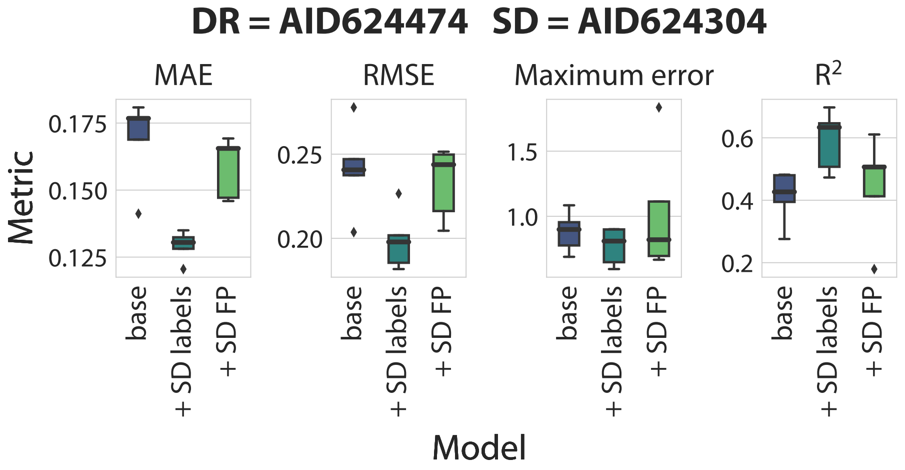

Figure S100. Test MAE, RMSE, maximum error, and R^2 for AID624474 – AID624304 for deep learning.

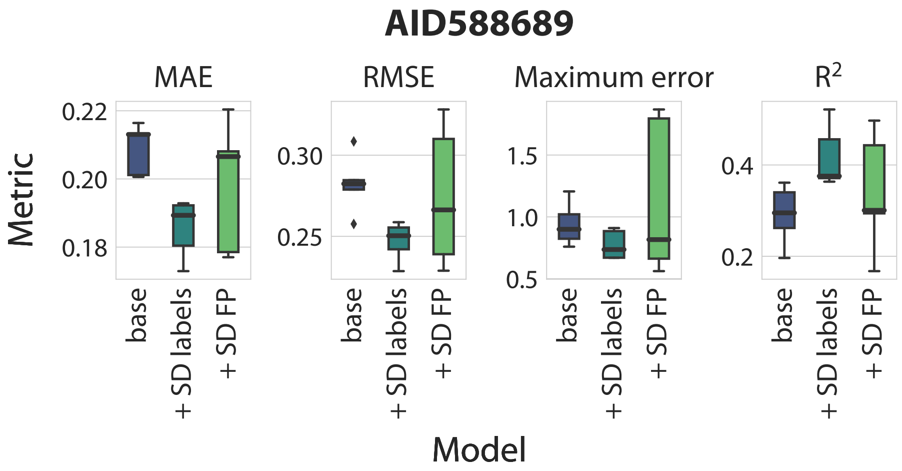

Figure S101. Test MAE, RMSE, maximum error, and R^2 for AID588689 – AID588689 for deep learning.

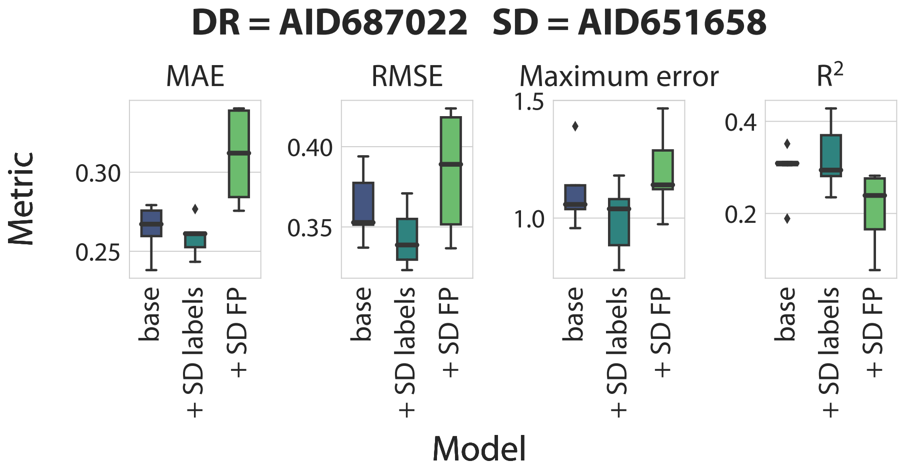

Figure S102. Test MAE, RMSE, maximum error, and R^2 for AID687022 – AID651658 for deep learning.

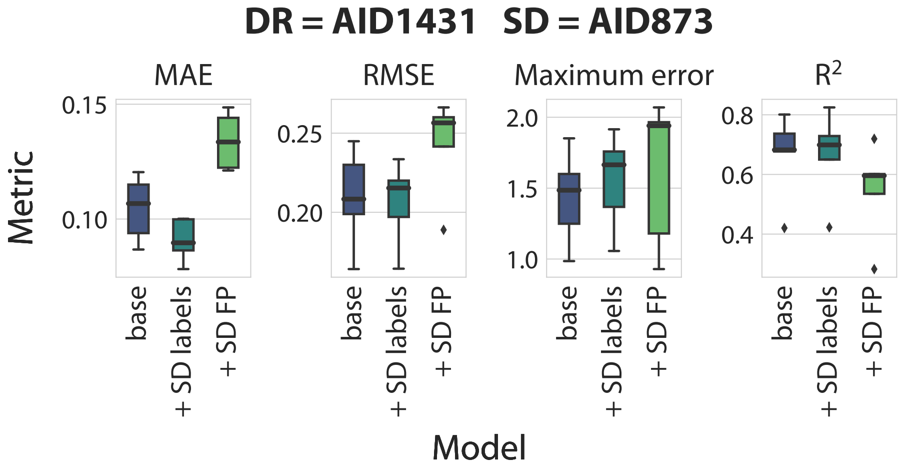

Figure S103. Test MAE, RMSE, maximum error, and R^2 for AID1431 – AID873 for deep learning.

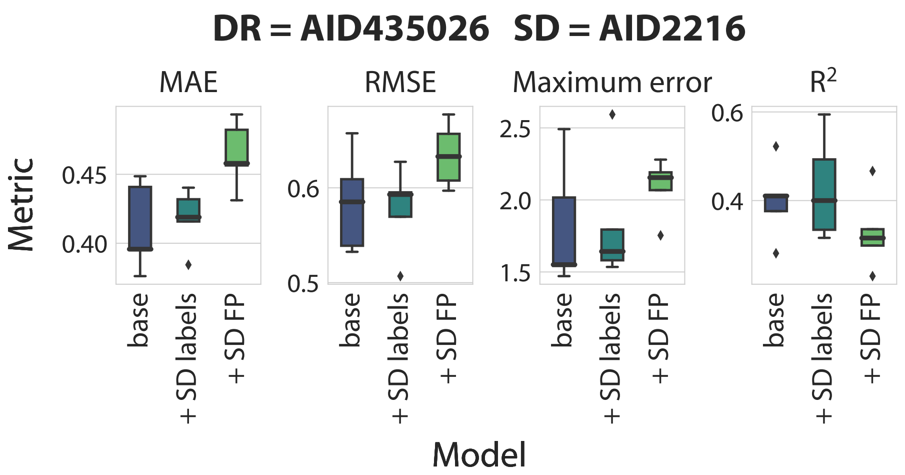

Figure S104. Test MAE, RMSE, maximum error, and R^2 for AID435026 – AID2216 for deep learning.

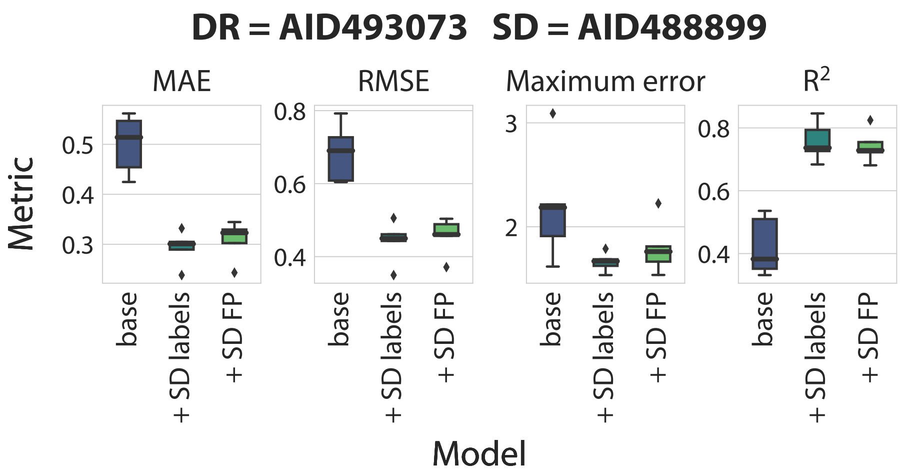

Figure S105. Test MAE, RMSE, maximum error, and R^2 for AID493073 – AID488899 for deep learning.

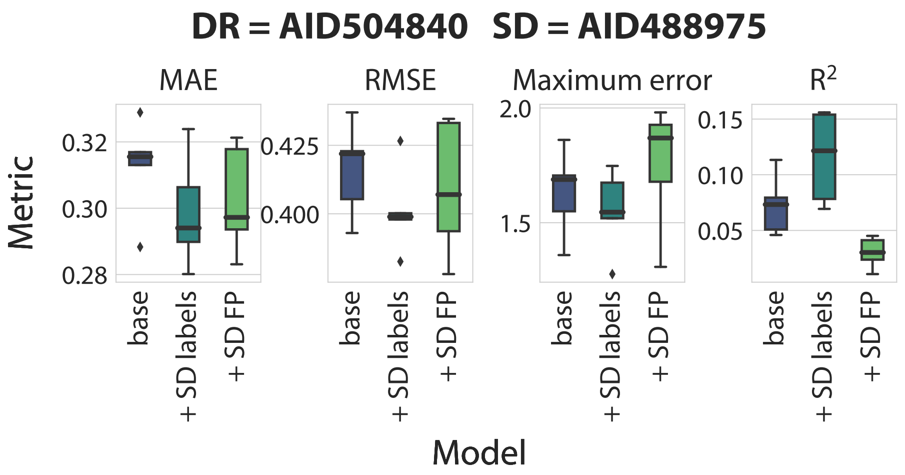

Figure S106. Test MAE, RMSE, maximum error, and R^2 for AID504840 – AID488975 for deep learning.

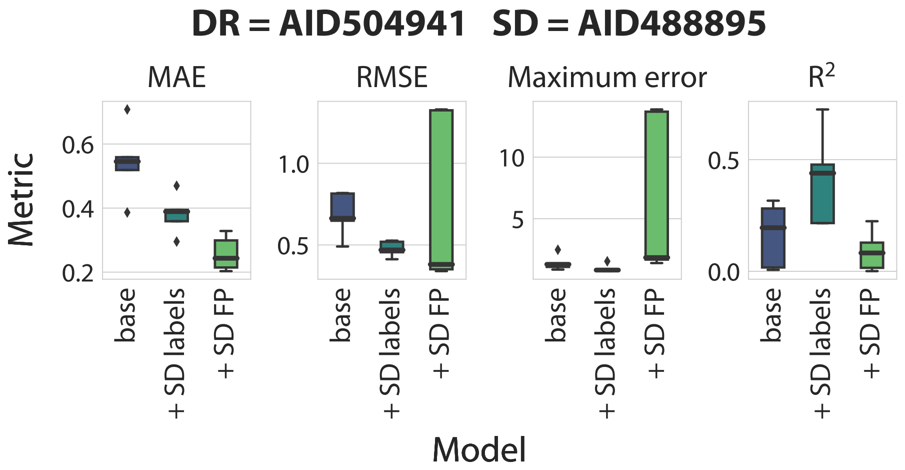

Figure S107. Test MAE, RMSE, maximum error, and R^2 for AID504941 – AID488895 for deep learning.

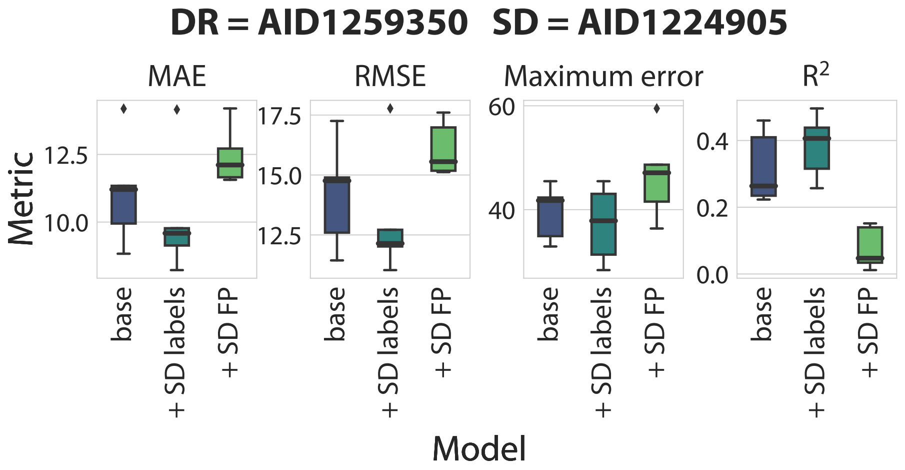

Figure S108. Test MAE, RMSE, maximum error, and R^2 for AID1259350 – AID1224905 for deep learning.

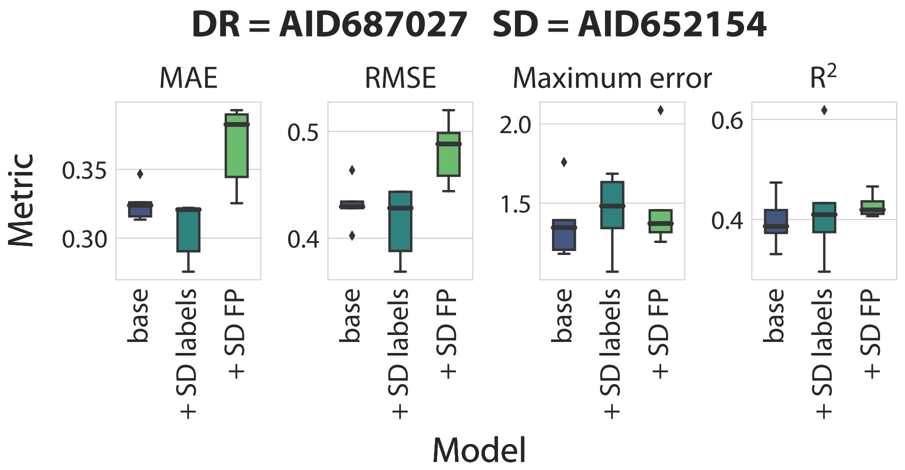

Figure S109. Test MAE, RMSE, maximum error, and R^2 for AID687027 – AID652154 for deep learning.

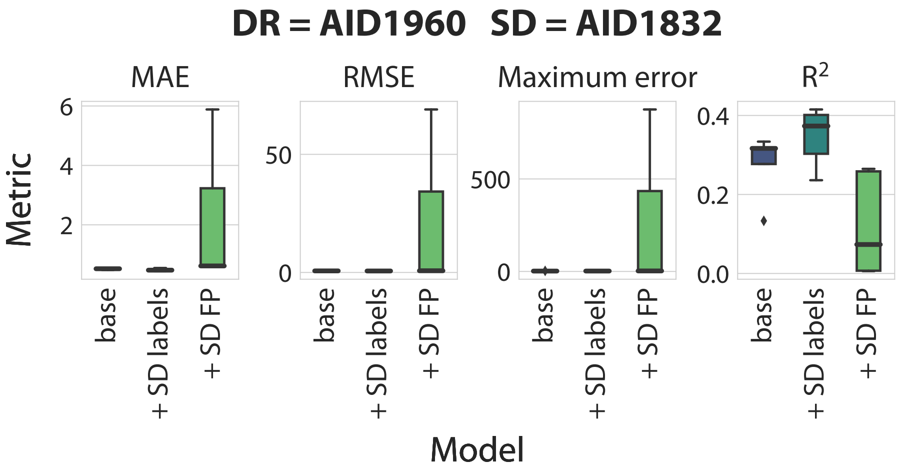

Figure S110. Test MAE, RMSE, maximum error, and R^2 for AID1960 – AID1832 for deep learning.

SI 13 Versions, revisions, and dates for all PubChem assays

Table S19. The versions, revisions, and last modification dates for all the reported PubChem assays.

AID	Version	Revision	Year	Month	Day
651658	1	1	2012	10	19
449756	1	1	2010	7	19
687027	1	1	2013	6	15
493155	1	1	2011	2	3
588489	1	1	2011	10	11
1117319	1	2	2015	3	23
1445	1	1	2008	12	8
435010	1	3	2011	10	18
489030	2	1	2011	4	14
488975	1	2	2010	11	17
720511	1	1	2013	7	16
2216	1	3	2009	12	23
720512	2	1	2013	9	26
2629	1	1	2010	3	22
1949	1	3	2009	10	13
488899	1	1	2010	10	25
1259375	1	1	2018	12	31
743445	1	1	2014	4	10
2097	1	2	2010	5	26
1662	1	2	2009	4	9
2227	1	2	2010	3	30
624273	1	1	2012	6	6
463203	1	1	2010	9	7
2221	1	2	2010	6	8
504582	2	1	2011	3	31
1431	1	3	2008	12	3
686996	1	1	2013	5	23
493091	1	1	2011	1	26
2247	1	2	2010	3	30
2099	1	1	2009	11	5
504313	1	1	2011	2	18
504558	1	1	2011	3	24
504941	1	1	2011	7	11
624326	1	1	2012	6	22
588689	1	1	2011	10	26
1979	1	1	2009	10	14
652115	1	1	2013	3	15
624474	1	4	2013	3	15
1259350	1	1	2018	12	18
1832	2	1	2009	9	29
504621	1	1	2011	3	30
1465	1	1	2008	12	30
1903	2	3	2011	3	4
1619	1	2	2009	3	27
2382	1	1	2010	2	19
504329	1	1	2011	2	22
624330	1	2	2012	7	3
2553	1	2	2010	3	21
485317	1	2	2010	10	18
1259418	1	1	2018	7	27
1259420	1	1	2018	7	27
2237	1	2	2010	3	30
449739	1	2	2011	3	9
449762	1	2	2010	7	23

Table S19. (continued from the previous page)

AID	Version	Revision	Year	Month	Day
651710	1	1	2012	10	26
504408	2	1	2011	3	7
687022	1	1	2013	6	6
435005	1	1	2010	6	18
652154	1	1	2013	3	21
485273	2	1	2010	10	4
602259	1	1	2012	2	14
1117362	1	2	2015	3	30
588524	2	1	2011	10	25
504840	1	2	2011	8	10
743267	1	1	2014	1	30
435026	1	1	2010	6	30
652162	2	1	2013	3	27
435023	1	1	2010	6	29
493073	1	1	2011	1	24
1259374	1	1	2018	12	31
1053173	1	1	2014	6	23
434954	1	1	2010	5	28
1914	1	2	2009	9	3
434941	1	2	2010	12	2
588549	1	1	2011	10	18
2650	2	1	2010	3	31
449749	1	2	2010	9	15
540271	1	1	2011	7	18
873	1	13	2008	11	19
720632	1	1	2013	9	7
540297	1	1	2011	7	23
434942	1	2	2010	12	2
488835	1	1	2010	10	20
2732	1	1	2010	3	31
588343	1	1	2011	9	7
588398	1	1	2011	9	22
488895	1	2	2010	11	17
602261	1	1	2012	2	16
2423	1	3	2011	10	11
720591	1	1	2013	8	27
624304	1	2	2013	3	15
1224905	2	1	2017	12	18
449750	1	2	2010	9	15
1960	1	1	2009	9	29
540268	1	1	2011	7	18
2098	1	1	2009	11	4
2696	1	1	2010	3	26
493248	1	1	2011	2	17
1259416	1	2	2018	7	27
1964	1	1	2009	10	1
434937	1	2	2010	12	2
720597	1	1	2013	9	3
489005	1	2	2011	3	9
652116	1	1	2013	3	15
435004	1	2	2011	1	27
